# High Level of Nonsynonymous Changes in Common Bean Suggests That Selection under Domestication Increased Functional Diversity at Target Traits

**DOI:** 10.3389/fpls.2016.02005

**Published:** 2017-01-06

**Authors:** Elena Bitocchi, Domenico Rau, Andrea Benazzo, Elisa Bellucci, Daniela Goretti, Eleonora Biagetti, Alex Panziera, Giovanni Laidò, Monica Rodriguez, Tania Gioia, Giovanna Attene, Phillip McClean, Rian K. Lee, Scott A. Jackson, Giorgio Bertorelle, Roberto Papa

**Affiliations:** ^1^Department of Agricultural, Food and Environmental Sciences, Università Politecnica delle MarcheAncona, Italy; ^2^Department of Agriculture, Università degli Studi di SassariSassari, Italy; ^3^Department of Life Sciences and Biotechnology, Università degli Studi di FerraraFerrara, Italy; ^4^Department of Plant Physiology, Umeå Plant Science Centre, Umeå UniversityUmeå, Sweden; ^5^Consiglio per la Ricerca in Agricoltura e l'Analisi dell'Economia Agraria, Centro di Ricerca per la CerealicolturaFoggia, Italy; ^6^Scuola di Scienze Agrarie, Forestali, Alimentari ed Ambientali, Università degli Studi della BasilicataPotenza, Italy; ^7^Department of Plant Sciences, North Dakota State UniversityFargo, ND, USA; ^8^Center for Applied Genetic Technologies, University of GeorgiaAthens, GA, USA

**Keywords:** cost of domestication, deleterious mutations, adaptation, recombination rate, selection

## Abstract

Crop species have been deeply affected by the domestication process, and there have been many efforts to identify selection signatures at the genome level. This knowledge will help geneticists to better understand the evolution of organisms, and at the same time, help breeders to implement successful breeding strategies. Here, we focused on domestication in the Mesoamerican gene pool of *Phaseolus vulgaris* by sequencing 49 gene fragments from a sample of 45 *P. vulgaris* wild and domesticated accessions, and as controls, two accessions each of the closely related species *Phaseolus coccineus* and *Phaseolus dumosus*. An excess of nonsynonymous mutations within the domesticated germplasm was found. Our data suggest that the cost of domestication alone cannot explain fully this finding. Indeed, the significantly higher frequency of polymorphisms in the coding regions observed only in the domesticated plants (compared to noncoding regions), the fact that these mutations were mostly nonsynonymous and appear to be recently derived mutations, and the investigations into the functions of their relative genes (responses to biotic and abiotic stresses), support a scenario that involves new functional mutations selected for adaptation during domestication. Moreover, consistent with this hypothesis, selection analysis and the possibility to compare data obtained for the same genes in different studies of varying sizes, data types, and methodologies allowed us to identify four genes that were strongly selected during domestication. Each selection candidate is involved in plant resistance/tolerance to abiotic stresses, such as heat, drought, and salinity. Overall, our study suggests that domestication acted to increase functional diversity at target loci, which probably controlled traits related to expansion and adaptation to new agro-ecological growing conditions.

## Introduction

Understanding how domestication has affected the level and organization of genetic diversity of crop germplasm, and identifying the genes/genomic regions that are responsible for the phenotypic variations of traits that distinguish wild forms from domesticated forms, are major goals not only for evolutionary biologists, but also for breeders who exploit this knowledge as a tool to modify breeding strategies and to more easily manipulate traits of interest (McCouch, 2004). One of the major effects of domestication is generally a reduction in the genetic diversity of the domesticated compared to the wild forms, as a consequence of both drift and selection at target loci (Glémin and Bataillon, 2009). The reduction in genetic diversity for domesticated forms is seen for numerous crops (Glémin and Bataillon, 2009; Bitocchi et al., 2013).

The recent literature describes the accumulation of deleterious mutations in domesticated germplasm as the so-called “cost of domestication” (Lu et al., 2006). This was seen in rice (Lu et al., 2006; Nabholz et al., 2014), tomato (Koenig et al., 2013), maize (Mezmouk and Ross-Ibarra, 2014), sunflower (Renaut and Rieseberg, 2015), barley and soybean (Kono et al., 2016), as well as for dogs (Cruz et al., 2008) and horses (Schubert et al., 2014). In each case, an excess of nonsynonymous substitutions was observed in the domesticated germplasm. The evolutionary forces that govern this phenomenon are essentially genetic drift and recombination rates. Domestication bottlenecks have strongly reduced the effective population sizes, and consequently, selection has been less effective in contrasting random genetic shifts of allele frequencies toward the fixing of deleterious mutations (Hill and Robertson, 1966). Genetic hitchhiking (Maynard Smith and Haigh, 1974) and background selection (Charlesworth et al., 1993, 1995) also reduce the overall efficacy of selection by reducing the locus-specific effective population size (Hill and Robertson, 1966). These processes are more pronounced in selfing species, for which recombination rates are lower compared to outcrossing species (Carvalho, 2003).

However, it has to be considered that the accumulation of nonsynonymous mutations might have resulted from the relaxation of selective constraints in a domesticated environment. This is the case for loci that are strongly influenced by natural selection in the wild forms, but not in the domesticated forms, and for loci that have lost their functionality (i.e., pseudogenes). It is also possible that selection during domestication favored beneficial nonsynonymous mutations (as novel or from standing variations) that enable domesticated forms to successfully compete (de Alencar Figueiredo et al., 2008; Bellucci et al., 2014a).

There are many examples in the literature where the genetic control of adaptation to domestication has been analyzed through identification of “selective sweeps” (i.e., detection of single loci that show changes in allelic frequencies due to selection). There are numerous bottom-up methods that use genotypic data without any prior information about phenotypes for the detection of sweep signals (for reviews see Luikart et al., 2003; Storz, 2005; Vitti et al., 2013). These require validation, because the procedures have high rates of false positives that are primarily due to the complex demographic histories and population structures that are not considered by the various models (Excoffier et al., 2009; De Mita et al., 2013; Lotterhos and Whitlock, 2014). Moreover, the role of hitchhiking has to be taken into account (Maynard Smith and Haigh, 1974); i.e., the “outlier” behavior of neutral loci that are physically linked to the “true” selected locus. False negatives, as outliers that are not detected by the neutrality tests under selection, have to be considered too. Indeed, false negatives can arise when the selection derived from standing variation rather than from a new mutation leads to a “soft sweep” (Hermisson and Pennings, 2005), or when the selection is too recent for fixation to have occurred (Hohenlohe et al., 2010). In both cases, weak signals will be obtained in selection tests.

*Phaseolus vulgaris* originated in Mesoamerica, followed by migration and adaptation into South America (Bitocchi et al., 2012). As a consequence, two geographically distinct and partially isolated gene pools were established, for Mesoamerica and the Andes, where at least two independent domestication events occurred, one for each gene pool (for review see Bellucci et al., 2014b). Recently, Bellucci et al. (2014a) compared the transcriptomes of a set of representative wild and domesticated *P. vulgaris* accessions from Mesoamerica, and showed that domestication affected not only genetic diversity, but also gene-expression patterns. Schmutz et al. (2014) analyzed whole genome sequencing data of DNA pools of wild and domesticated accessions from both of these two gene pools, and identified a set of candidate genes in the Mesoamerican and Andean gene pools that are putatively implicated in flowering time and seed size. They also provided the first hypotheses on convergent evolution of different populations within the same species.

To investigate the major effects of domestication on domesticated germplasm from Mesoamerica, we analyzed nucleotide sequences from a set of 49 gene fragments from a sample of 39 wild and domesticated accessions. We performed separate diversity and selection analyses for coding and noncoding regions, and compared the data relative to the same loci to those of different studies, to obtain evidence of selection of candidate genes during domestication. The putative functions of the candidate genes were determined, to reveal associations to domestication in other species, using either direct experimentation or because their function was previously known.

## Materials and methods

### Plant materials

A set of 45 *P. vulgaris* accessions was used. Each accession was represented by a highly homozygous inbred line that was obtained by two cycles of selfing through single seed descent. Of these, 39 accessions were from Mesoamerica (19 wild, 20 domesticated). Four Andean accessions (two wild, two domesticated) and two wild accessions characterized by phaseolin type I (Debouck et al., 1993; Kami et al., 1995) from northern Peru and Ecuador were also included. The accessions were selected on the basis of well-detailed molecular characterization of a wider sample of *P. vulgaris* that is representative of the different gene pools that characterize this species (Rossi et al., 2009; Nanni et al., 2011; Bitocchi et al., 2012, 2013; Desiderio et al., 2013), to maximize the genetic diversity of the initial complete sample. Moreover, two accessions each of *Phaseolus coccineus* and *Phaseolus dumosus* were included as controls, as these represent the most closely related legume species to *P. vulgaris*. A complete list of the accessions studied, along with their “passport” information, is available in Table S1. Genomic DNA was extracted from each accession from young leaves of a single, greenhouse-grown, plant using the miniprep extraction method (Doyle and Doyle, 1987).

### PCR and sequencing

A total of 49 gene regions (from ~150 to 900 bp in size) across the common bean genome were used, 48 of which were from the literature (Bellucci, 2006; Hougaard et al., 2008; McConnell et al., 2010; Nanni et al., 2011; Goretti et al., 2014), while locus AN-Pv26.1 was developed in this study. The complete list of the loci is given in Table S2, along with the gene functions, references for information about primer sequences, PCR and sequencing procedures, and locations on the reference genome (Schmutz et al., 2014), identified through BLASTN (Altschul et al., 1997; www.phytozome.net).

The sequences of the 45 *P. vulgaris* accessions for the Leg044, Leg100, Leg133, Leg223, and PvSHP1 loci were available from Nanni et al. (2011) and Bitocchi et al. (2012, 2013). The sequences of 44 loci for 22 *P. vulgaris* accessions were obtained from the study of Goretti et al. (2014). For the remaining 23 *P. vulgaris* accessions, the sequences were determined as part of the present study. The sequences of the new gene fragment developed in this study (AN-Pv26.1) for all of the 45 accessions were obtained in the present study (see Table S2), as well as those for the *P. coccineus* and *P. coccineus* accessions for 48 loci (sequences for PvSHP1 locus were from Nanni et al., 2011). The GeneBank accession numbers of the sequences developed in this study are KY194860-KY195914 (see Supplementary File S1 for complete alignments of the AN-Pv41 and AN-Pv42 loci).

For 45 of the 49 loci, the structures (exons, introns, 3′-untranslated regions [UTRs], 5′-UTRs) were available from Bitocchi et al. (2012) and Goretti et al. (2014). The four fragments with unknown structures were AN-Pv48, Leg443, gssE19, and gssE28 loci. Forty-two loci included exon regions, while 47 included noncoding regions (introns and/or 5′-UTRs, 3′-UTRs).

### Diversity analysis

Sequence alignment and editing were performed using MUSCLE, version 3.7 (Edgar, 2004) and BIOEDIT, version 7.0.9.0 (Hall, 1999). Insertions/deletions (indels) were not included in the analysis. Diversity analysis was carried out considering different partitions of the accessions: the *P. vulgaris* sample; the *P. vulgaris* Mesoamerican sample; and the Mesoamerican wild (MW) and domesticated (MD) populations.

The following diversity estimates were computed: *V* (number of variable sites); η (number of mutations); *Pi* (parsimony informative sites); *S* (singleton variable sites); *Syn* (number of synonymous changes); *Nonsyn* (number of nonsynonymous changes); *H* (number of haplotypes); *Hd* (haplotype diversity; Nei, 1987); π (Tajima, 1983); and θ (Watterson, 1975). Separate estimates were made for the whole sequences and the coding (exons) and noncoding (introns, 3′UTR, 5′UTR) regions. The divergence between the MW and MD populations was investigated by computing the number of shared and unique mutations between the wild and domesticated populations within each gene pool, and the F_ST_ statistic (Hudson et al., 1992) with permutation tests (Hudson, 2000; 1000 replicates). All of these calculations were carried out using DnaSP version 5.00 (Librado and Rozas, 2009). Analysis of molecular variance (AMOVA) was also carried out to investigate the distribution of the genetic differentiation between the MW and MD populations, using the program GenAlEx (ver. 6.5) (Peakall and Smouse, 2012).

As proposed by Vigouroux et al. (2002), to measure the loss of nucleotide diversity in MW vs. MD we used the statistic *L*π = 1 − (π_MD_/π_MW_), where π_MW_ and π_MD_ are the nucleotide diversities in the MW and MD forms, respectively. The loss of nucleotide diversity was computed from both the mean estimates of π and θ (*L*π and *L*_θ_, respectively), and averaging the *L*π and *L*_θ_ of each locus (*L*π^1^ and L_θ_^1^) for the MW/ MD comparisons.

Wilcoxon–Kruskal–Wallis nonparametric test (Sokal and Rohlf, 1995) was used to test the differences among the MW and MD accessions of *P. vulgaris* for the genetic diversity estimates (π and θ), considering the whole sequence, and the coding and noncoding regions.

The average rate of synonymous (*dS*) and nonsynonymous (*dN*) substitutions per site were computed in MEGA 7 (Kumar et al., 2016) using the modified Nei-Gojobori method (Nei and Gojobori, 1986), with the Jukes-Cantor correction (Jukes and Cantor, 1969) for multiple substitutions. Standard errors were estimated after 1000 bootstrap replicates. This computation was carried out for each locus for both the MW and MD populations. Nonparametric Wilcoxon signed-rank test for two groups, i.e., pairs of estimates for each locus (Sokal and Rohlf, 1995), was used to test whether the differences between the *dN*/*dS* estimates computed for each locus were significantly different between MW and MD.

### Divergence and positive selection

Regions showing evidence of putative selection were detected following the same approach used in a previous transcriptome scan (Bellucci et al., 2014a). A selection index was calculated separately for the exonic and intronic fragments, and significance was tested using coalescent simulations that take into account the demographic history of the common bean. Monomorphic genes (4 out of 43) were excluded from this analysis.

Briefly, the selection index combines three normalized statistics: molecular F_ST_ (Excoffier et al., 1992), the Shriver et al. (2004) branch-length statistic, and a standardized difference between genetic variation in the wild and the domesticated forms. The first and the second statistics aim to capture genes highly differentiated between the wild and domesticated pools, while the third is based on the likely change of diversity occurring during selection.

**Figure 1:**
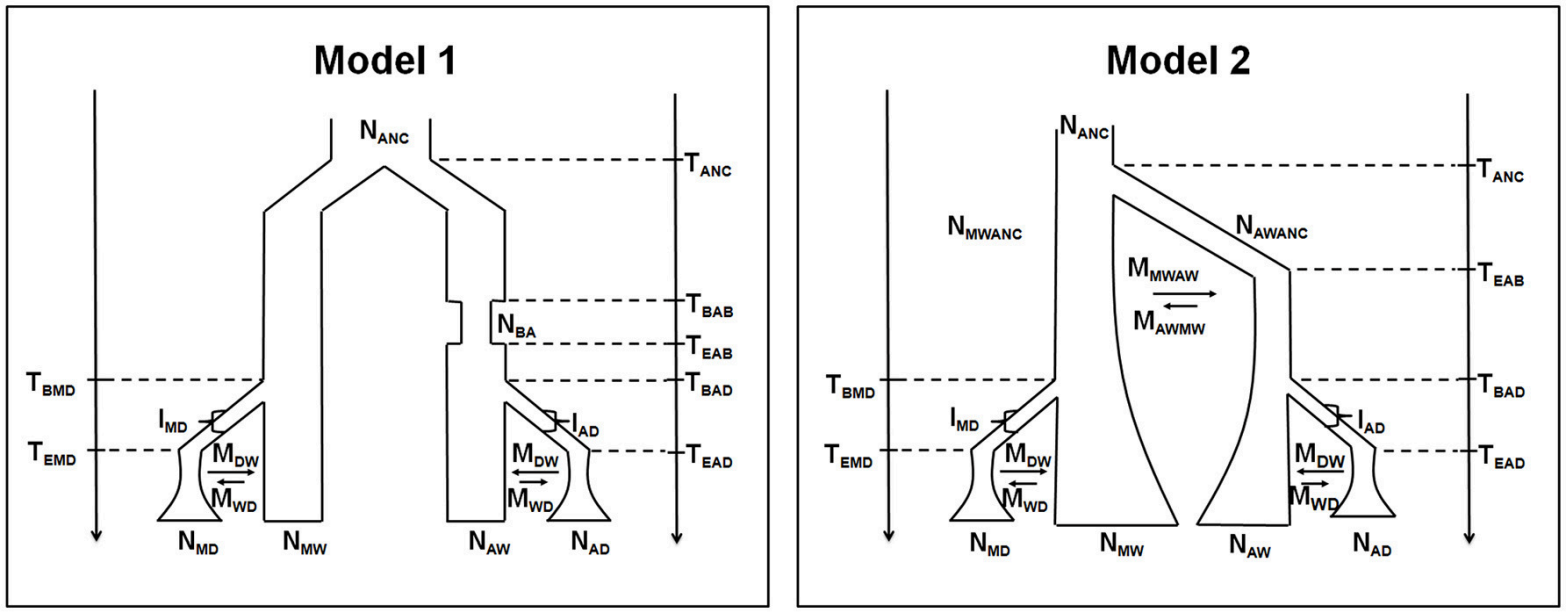
**Demographic scenarios for the Mesoamerican and Andean populations**. Parameters: *T*_ANC_, divergence time between Andean and Mesoamerican gene pools; *T*_BAB_, time of the beginning of the Andean founder bottleneck; *T*_EAB_, time of the ending of the Andean founder bottleneck; *T*_BMD_, time of the beginning of the Mesoamerican domestication; *T*_BAD_, time of the beginning of the Andean domestication; *T*_EMD_, time of the ending of the Mesoamerican domestication; *T*_EAD_, time of the ending of the Andean domestication; *N*_ANC_, ancestral effective population size; *N*_BA_, Andean effective population size during the founder bottleneck; *N*_MWANC_, ancestral wild Mesoamerican effective population size; *N*_AWANC_, ancestral wild Andean effective population size; *N*_MD_, effective population size of the domesticated Mesoamerican population; *N*_MW_, effective population size of the wild Mesoamerican population; *N*_AW_, effective population size of the wild Andean population; *N*_AD_, effective population size of the domesticated Andean population; *I*_*MD*_, intensity of the domestication bottleneck in Mesoamerica (in percentage); *I*_AD_, intensity of the domestication bottleneck in the Andes (in percentage); *M*_WD_, migration rate from wild to domesticated population; *M*_DW_, migration rate from domesticated to wild population; *M*_MWAW_, migration rate from wild mesoamerican to andean wild population; *M*_AWMW_, migration rate from wild Andean to Mesoamerican wild population.

The distribution of the selection index under neutrality was obtained by simulation, using the ABCtoolbox package (Wegmann et al., 2010). Two different models were used, based on previous reconstructions of the demographic history of the common bean (Mamidi et al., 2011, 2013; Schmutz et al., 2014; see also Figure 1). In both models, the Mesoamerican and the Andean populations derive from a common ancestral pool, and two independent domestication events occurred in each population after divergence. The two models differ in the timing of the bottleneck event associated with the colonization of the Andes. Model 1 assumes that this event was recent, whereas Model 2 implies a bottleneck just after the separation from the Mesoamerican gene pool. Models 1 and 2 also assume a different population size dynamic of the wild pools in recent times, as constant or exponentially growing, respectively. Prior distributions of the demographic parameters were used to take into account the uncertainty around estimates (see Table S3 for complete list of the parameters used in the simulations, and their prior distributions).

For each exonic and intronic fragment, regions with gaps or missing data were removed from the alignment, and only polymorphic fragments were analyzed. One hundred thousand simulations were performed randomly by sampling model parameters from their prior distributions. The length of the simulated fragments was not fixed, but varied according to the distribution frequency observed in the real data. The prior distribution of the mutation rate had different means for the intronic and exonic fragments. A mean mutation rate of 1.0 × 10^−8^ per site per generation was applied to introns, and a 10-fold lower rate was used for the exons (Bellucci et al., 2014a). The *P*-values for each fragment were calculated as the fraction of simulated indices larger than the observed value. *P*-values were then corrected using the Benjamini Hochberg approach (Benjamini and Hochberg, 1995, implemented in the R function p.adjust), and gene portions with false discovery rate <5% were considered as putatively under selection.

We searched for independent evidence of selection signatures for loci detected as outliers. Thus, we compared our data with those obtained for the same loci in other studies (Bellucci et al., 2014a; Schmutz et al., 2014). Moreover, we used the single nucleotide polymorphism (SNP) data given in Rodriguez et al. (2016) in a wider sample of wild and domesticated *P. vulgaris* accessions from both the Andean and Mesoamerican gene pools, to perform a selection test to investigate whether the SNPs derived from loci that we detected as outliers show signatures of selection during domestication, with these different data and samples.

We mapped the physical positions of all 42 loci using the coding sequence derived from the reference transcriptome (27,243 contigs) developed by Bellucci et al. (2014a). Each locus was mapped to the reference sequence using Blast Like Alignment Tool (BLAT; Kent, 2002), version 34, implemented in the iPlant Collaborative web portal (Oliver et al., 2013), with default parameters. The data from BLAT were subsequently filtered to retain the best alignments by exploiting the Best Hit for Blat Output tool, version 34, with matches obtained for 25 loci, with an *E*-value < 10E^−24^. The results were validated by a BLASTN analysis (Altschul et al., 1997) performed using default settings when aligning each locus against its respective reference contig. A comparison of the data from the selection analysis with those obtained from the Bellucci et al. (2014a) study was then carried out. Moreover, the mapping of the 49 loci on the reference genome (Schmutz et al., 2014) allowed a further comparison with genomic regions (windows)/genes identified as affected by domestication in both of the two gene pools by Schmutz et al. (2014).

The AN-Pv loci were from the study of Goretti et al. (2014), where their sequence data were used to design primers for the amplification of SNP markers for the KASPar genotyping platform (LGC Genomics, Teddington, Middlesex, UK). In particular, 35 SNPs were from 22 of the 34 AN-Pv loci used in the present study (see Supplementary Materials from Goretti et al., 2014). A set of 131 SNPs from Goretti et al. (2014) augmented with SNPs developed by Cortés et al. (2011) was used to genotype 417 Mesoamerican and Andean wild and 160 domesticated *P. vulgaris* accessions by Rodriguez et al. (2016). These data from a wide sample of accessions were used to test SNPs for signatures of selection during domestication in the two gene pools of the species. We applied the F_ST_-outlier detection method developed by Beaumont and Nichols (1996), which uses the available data to derive a distribution of F_ST_ and expected heterozygosity, and was implemented in the LOSITAN workbench (Antao et al., 2008). The infinite alleles model was used and 50,000 simulations were performed. We used a conservative false discovery rate (0.01), a 99.0% confidence interval, and options for neutral and forced mean F_ST_. Deviations of loci from the expected distribution of neutral markers were identified based on excessively high or low F_ST_. Outliers suggest directional selection when F_ST_ is greater than expected, or balancing selection when F_ST_ is lower than expected.

## Results

A set of 49 gene fragments were sequenced in a sample of 45 accessions of *P. vulgaris*, 39 from the MW and MD forms of the Mesoamerican gene pool. High quality sequence data were obtained for all of the accessions for 37 out of the 49 loci, while for the remaining 12 loci, data from one (AN-Pv8, AN-Pv68, Leg044) to seven (AN-PvCO) accessions were not of sufficient quality to be retained in the analyses (Table S4). For 44 and 42 out of the 49 gene fragments, we obtained high quality sequences for *P. dumosus* and *P. coccineus* accessions, respectively.

Forty-seven loci were located on the reference genome (Schmutz et al., 2014) through a BLASTN analysis (Table S2 and Figure S1), and were found on all chromosomes except chromosome 3. The sequenced region for each locus encompassed between 133 bp (AN-Pv41) and 889 bp (PvSHP1), with a mean of ~440 bp per locus. Overall, ~21.5 kb per accession were available (Table S4).

### Genetic diversity analysis

The genetic diversity statistics for each locus were computed for the whole sample, the Mesoamerican, and for the MW and MD populations; furthermore this was done for the whole sequence, the coding regions, introns, 3′UTRs, and 5′UTRs, and considering all of the noncoding regions together (Tables S4–S7). A summary of the genetic diversity estimates computed considering the whole sample of *P. vulgaris* and the Mesoamerican accessions is given in Table S8. Two loci (AN-Pv9, AN-Pv55) were monomorphic in the whole sample, while two other loci (AN-Pv32, gssE20) were monomorphic only in the Mesoamerican accessions (Tables S4, S8). A total of 465 and 425 SNPs were identified, with a mean of 9.5 and 8.7 SNPs per locus, for the whole sample and the Mesoamerican population, respectively.

All genetic diversity estimates (*V, H, Hd*, π, and θ) were higher in MW compared to MD (Table 1) for the whole sequence, and the coding and noncoding regions. In particular, the π estimate was 2.0-, 1.95- and 2.22-fold higher in MW over MD for the whole sequence and the coding and noncoding regions, respectively; the same trend was also observed for θ (Table 1). Differences in genetic diversity estimates between the wild and domesticated populations were significant using the Wilcoxon–Kruskal–Wallis nonparametric test (whole sequence, *P* <0.001; and coding, *P* <0.02; noncoding, *P* < 0.005, regions). The loss of diversity estimates, which were computed both by considering the mean estimates of diversity (*L*_π_, *L*_θ_) and by averaging the loss of diversity values for each locus (*L*_π_^1^, *L*_θ_^1^), clearly highlighted a strong reduction in genetic diversity (~50%, considering the whole sequence) of the domesticated as compared to the wild forms. Moreover, the loss of genetic diversity was slightly higher in noncoding (*L*_π_ = 0.55, *L*_θ_ = 0.57) than coding (*L*_π_ = 0.49, *L*_θ_ = 0.43) regions (Table 2). The SNP frequencies were obtained by dividing the overall number of SNPs by the total length of sequences, and in MW these were 0.019, 0.012, and 0.026 for the whole sequence, and the coding and noncoding regions, respectively. These values are higher than those for MD (0.010, 0.007, and 0.012, respectively) (Table 3).

**Table 1:** **Summary of the genetic diversity estimates computed for Mesoamerican wild and domesticated *P. vulgaris* accessions, considering the whole sequences and the coding (exons) and noncoding (introns, 3′UTR, 5′UTR) regions separately**.

**Genetic diversity estimates**	**Whole sequence**		**Coding regions**		**Noncoding regions**							
					**Introns**		**5′-UTR**		**3′-UTR**		**Overall**	
	**MW**	**MD**	**MW**	**MD**	**MW**	**MD**	**MW**	**MD**	**MW**	**MD**	**MW**	**MD**
N. loci	49	49	42	42	32	32	2	2	7	7	37	37
*N*^1^	18.6	19.7	18.7	19.8	18.7	19.8	18.0	20.0	19.0	20.0	18.7	19.8
*V*^2^	411	218	133	78	218	103	8	0	16	11	242	114
η^2^	418	219	134	78	224	104	8	0	16	11	248	115
*S*^2^	123	67	52	28	60	35	1	0	5	1	66	36
*Pi*^2^	288	151	81	50	158	68	7	0	11	10	176	78
*Syn*^2^	/	/	82	45	/	/	/	/	/	/	/	/
*Nonsyn*^2^	/	/	50	32	/	/	/	/	/	/	/	/
*H*^1^	4.8	2.4	3.1	2.0	4.0	1.9	5.0	1.0	2.4	1.9	4.0	1.9
*Hd*^1^	0.58	0.27	0.38	0.20	0.46	0.20	0.61	0.00	0.35	0.16	0.48	0.20
^1^π × 10^−3^	5.39	2.70	3.29	1.69	7.63	3.29	4.49	0.00	5.54	3.51	7.66	3.45
^1^θ × 10^−3^	5.26	2.64	3.09	1.77	7.61	3.11	4.04	0.00	5.11	3.46	7.47	3.24

1 *Average estimate among loci: N, sample size; H, number of haplotypes; Hd, haplotype diversity; π × 10^−3^ and θ × 10^−3^, two measures of nucleotide diversity from Tajima (1983) and Watterson (1975), respectively*.

2 *Sum of the single locus estimates: V, variable sites; η, total number of mutations; S, singleton variable sites; Pi, parsimony informative variable sites; Syn, total number of synonymous changes; Nonsyn, total number of nonsynonymous changes*.

*MW, Mesoamerican wild; MD, Mesoamerican domesticated*.

**Table 2:** **Loss of nucleotide diversity in domesticated compared to wild Mesoamerican accessions of *P. vulgaris*, computed considering the whole sequences and the coding (exons) and noncoding (introns, 3′UTR, 5′UTR) regions separately**.

**Regions**	***L*****_π_**	**$L_{\pi }^{1}$**	***L*****_θ_**	***L*****_θ_^1^**
Whole sequence	0.50	0.57	0.50	0.53
Coding	0.49	0.55	0.43	0.42
Noncoding	0.55	0.57	0.57	0.60

*Monomorphic loci were excluded, as their loss of diversity cannot be calculated; loss of nucleotide diversity computed from the average estimates of π and θ (L_π_ and L_θ_, respectively) and averaging the L_π_ and L_θ_ of each locus ($L_{\pi }^{1}$ and $L_{\theta }^{1}$) for the MW/MD comparison*.

**Table 3:** **SNP frequency computed considering the different populations (whole sample, *P. vulgaris* sample, Mesoamerican accessions, MW, MD) and sequence regions (whole sequence, coding, and noncoding regions), separately**.

**Regions**	**Whole sample**	***P. vulgaris***	**Mesoamerican**	**MW**	**MD**
Whole sequence	0.031	0.021	0.019	0.019	0.010
Coding	0.021	0.013	0.013	0.012	0.007
Noncoding	0.040	0.029	0.026	0.026	0.012

**Figure 2:**
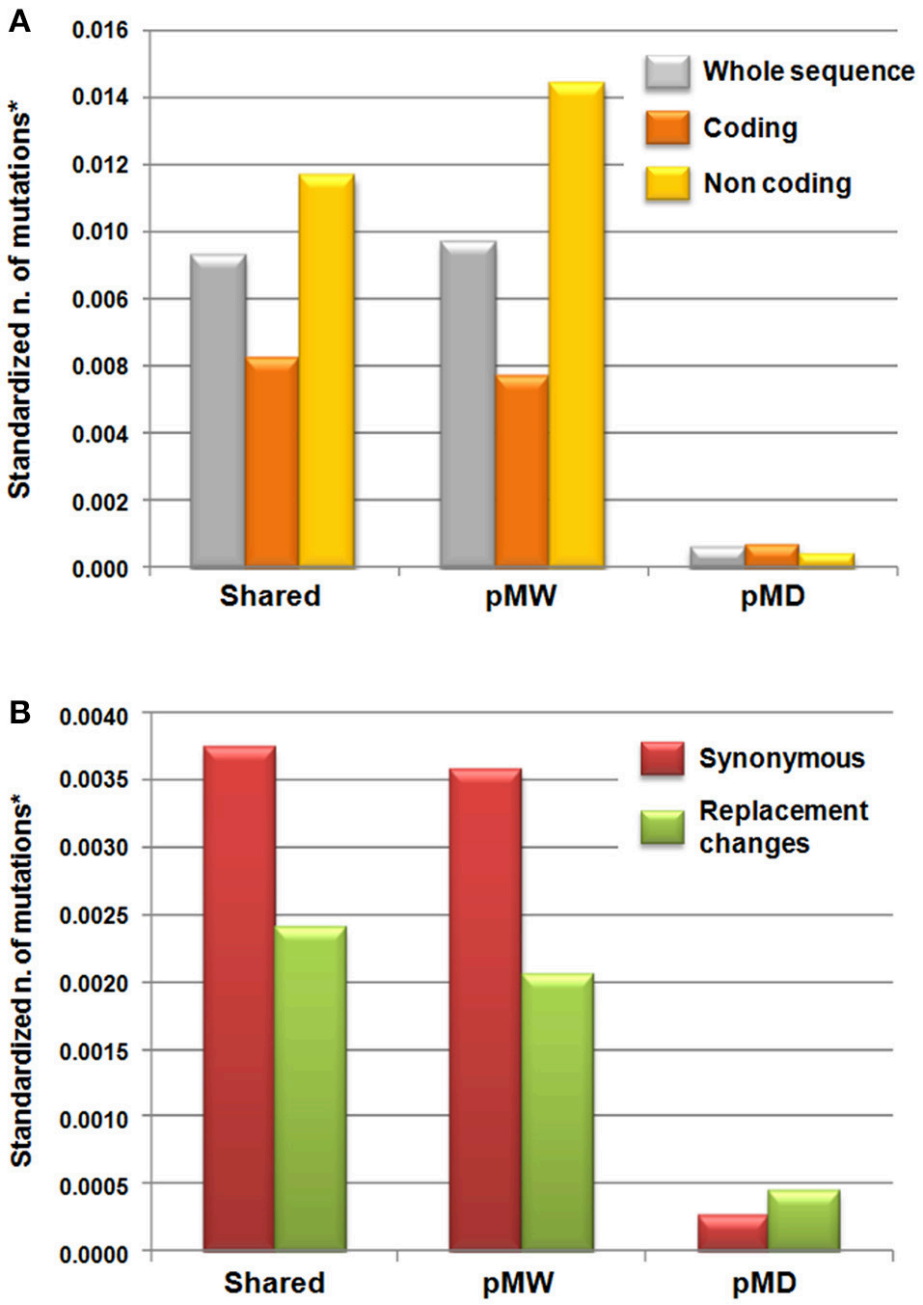
**Mutation patterns across the wild and domesticated Mesoamerican accessions of common bean (*P. vulgaris*); shared, unique in Mesoamerican wild (pMW) and unique in domesticated (pMD) populations (A)** for the whole sequence (gray), coding (orange), and noncoding (yellow) regions. **(B)** For the coding regions, considering subdivision in synonymous (violet) and replacement (green) changes. ^*^The number of mutations was standardized for the total length of the different sequence regions.

The F_ST_ estimates and the patterns of shared and unique mutations in the MW and MD populations are reported in Table 4. On a total of 45 polymorphic loci, 26 (58%) showed F_ST_ estimates significantly different from zero, and the mean F_ST_ was 0.16 (Table 4). A significant genetic differentiation between MW and MD (*P* < 0.0001) resulted also from AMOVA analysis (average F_ST_ = 0.16, Table S9). The total number of shared mutations between the MW and MD populations was 205. The number of polymorphic mutations in the MW population that were monomorphic mutations in the MD population was 213, while the MD population showed only 14 polymorphic mutations that were monomorphic in the MW accessions (Figure 2A). As expected, in the coding regions, the number of mutations was lower compared to that in the noncoding regions, for both shared mutations and those polymorphic uniquely in MW. However, the opposite was observed for unique mutations in MD. Indeed, in MD, the number of mutations in the coding regions was higher than that in the noncoding regions (*P* = 0.02, binomial test; null hypothesis: number of mutations in coding regions private in MD equal to the observed fraction of mutations in coding regions private in MW). In particular, eight sites that were polymorphic in MD but monomorphic in MW were identified in the coding regions, while only four were found in the noncoding regions (Figure 2A). The eight polymorphic sites in the MD but not in the MW coding regions implied an amino-acid replacement in five cases. This trend was different from that observation for the shared and unique mutations in MW; indeed, 42 (49.4%) synonymous changes were shared, 40 (47.1%) were unique for MW, and 3 (3.5%) were unique for MD. In contrast, considering replacement changes, 27 (49.1%) were shared, while 23 (41.8%) were unique MW polymorphic mutations, and 5 (9.1%) were unique for MD (Figure 2B).

**Table 4:** **F_ST_ estimates and mutation patterns across the wild and domesticated Mesoamerican accessions of common bean (*P. vulgaris*) for each locus and for the whole sequences and the coding (exons) and noncoding (introns, 3′UTR, 5′UTR) regions separately**.

	**Locus**	**Variable**											
		**Whole sequence**				**Coding**				**Noncoding**			
		***F_ST_***	***SM***	***P_MW_***	***P_MD_***	***F_ST_***	***SM***	***P_MW_***	***P_MD_***	***F_ST_***	***SM***	***P_MW_***	***P_MD_***
1	AN-Pv1	0.12^*^	0	6	0	0.06^*^	0	5	0	0.22^*^	0	1	0
2	AN-Pv2	0.01	4	1	0	0.02	1	0	0	0	3	1	0
3	AN-Pv3	0.06^*^	1	6	0	0.08	1	3	0	0	0	3	0
4	AN-Pv4	0.16^*^	1	2	0	na	na	na	na	0.16^*^	1	2	0
5	AN-Pv5	0.04	0	3	0	na	na	na	na	0.04	0	3	0
6	AN-Pv8	0.21^***^	2	4	1	0.21^**^	2	4	1	/	/	/	/
7	AN-Pv9	na	na	na	na	na	na	na	na	na	na	na	na
8	AN-Pv10	0.02	5	3	0	0.01	4	0	0	0.05	1	3	0
9	AN-Pv16	0.33^***^	0	7	0	/	/	/	/	0.33^**^	0	7	0
10	AN-Pv17	0.11	0	1	0	na	na	na	na	0.11	0	1	0
11	AN-Pv18	0.01	0	9	1	0.01	0	9	1	/	/	/	/
12	AN-Pv22	0.46^***^	4	7	0	0.44^***^	3	4	0	0.50^***^	1	3	0
13	AN-Pv26.1	0.65^***^	9	7	0	0.57^***^	3	4	0	0.68^***^	6	3	0
14	AN-Pv28	0.01^*^	5	1	0	0.01	1	0	0	0.01^*^	4	1	0
15	AN-Pv29	0.09	3	1	1	0.11	1	0	0	0.08	2	1	1
16	AN-Pv30	0.19^***^	3	1	0	0.19^**^	3	1	0	/	/	/	/
17	AN-Pv32	na	na	na	na	na	na	na	na	na	na	na	na
18	AN-Pv33	0.37^***^	0	4	0	0.37^***^	0	4	0	/	/	/	/
19	AN-Pv35	0.06	0	2	0	na	na	na	na	0.06	0	2	0
20	AN-Pv41	0.00	1	2	0	/	/	/	/	0	1	2	0
21	AN-Pv42	0.02	3	2	0	/	/	/	/	0.02	3	2	0
22	AN-Pv44	−0.01	3	0	1	−0.01	3	0	1	na	na	na	na
23	AN-Pv46	0.25^**^	3	1	0	0.25^*^	3	1	0	na	na	na	na
24	AN-Pv47	0.23^*^	8	3	0	0.18^*^	4	2	0	0.28^*^	4	1	0
25	AN-Pv48	0.01	17	2	2	/	/	/	/	/	/	/	/
26	AN-Pv51	0.08^***^	12	7	0	0.08^***^	5	3	0	0.07^*^	7	4	0
27	AN-Pv54	0.03	5	1	2	0.03^*^	5	1	2	/	/	/	/
28	AN-Pv55	na	na	na	na	na	na	na	na	na	na	na	na
29	AN-Pv57	−0.03	11	2	0	−0.04	3	2	0	−0.03	8	0	0
30	AN-Pv63	0.19^***^	5	3	0	0.19^***^	5	3	0	/	/	/	/
31	AN-Pv64	0.25^***^	0	8	0	na	na	na	na	0.25^***^	0	8	0
32	AN-Pv66	0.02^*^	0	4	0	0.02	0	4	0	/	/	/	/
33	AN-Pv68	−0.03	8	9	1	−0.02	4	5	0	−0.03	4	4	1
34	AN-Pv69	0.59^***^	0	2	0	0.22^*^	0	1	0	0.72^***^	0	1	0
35	gssE18	0.20^***^	4	3	1	0.19^*^	2	0	0	0.21^***^	2	3	1
36	gssE19	0.08	0	2	0	/	/	/	/	/	/	/	/
37	gssE20	na	na	na	na	na	na	na	na	na	na	na	na
38	gssE28	0.00	4	6	0	/	/	/	/	/	/	/	/
39	AN-PvCO	0.05	11	5	1	0.05	6	1	1	0,05	5	4	0
40	AN-TGA	0.23^***^	3	11	0	0.00	0	1	0	0.23^***^	3	10	0
41	AN-DNAJ	0.41^***^	3	3	1	0.41^***^	3	3	1	/	/	/	/
42	g510	0.26^***^	8	2	0	0.29^**^	7	1	0	−0.04	1	1	0
43	g523	0.41^***^	2	0	1	0.31^**^	1	0	1	0.42^**^	1	0	0
44	Leg044	−0.02	10	12	0	na	na	na	na	−0.02	10	12	0
45	Leg100	0.08^***^	22	18	1	na	na	na	na	0.08^***^	22	18	1
46	Leg133	0.35^***^	1	12	0	0.06	0	1	0	0.36^***^	1	11	0
47	Leg223	0.33^***^	0	6	0	na	na	na	na	0.33^***^	0	6	0
48	Leg443	0.00	3	2	0	/	/	/	/	/	/	/	/
49	PvSHP1	0.22^***^	21	20	0	0.00	0	1	0	0.22^***^	21	19	0
	Overall	/	205	213	14	/	70	64	8	/	56	71	4
	Mean	0.16	/	/	/	0.14	/	/	/	0.17	/	/	/

*SM, shared mutations between MW and MD; P_MW_, mutations polymorphic in MW and monomorphic in MD; P_MD_, mutations polymorphic in MD and monomorphic in MW; F_ST_ probability*:

* *P < 0.05*;

** *P < 0.01; P < 0.001*;

*** *P < 0.0001; na, not applicable, as the loci monomorphic in all Mesoamerican accessions; /, loci with no structure identified or with absence of coding or noncoding regions in their sequence*.

We compared the MW and MD populations for differences between *dN* and *dS* computed for each locus (see Table S10) using nonparametric Wilcoxon signed-ranks tests for two groups (Sokal and Rohlf, 1995). The *dn*/*dS* estimates in MD were significantly higher than those in MW (*P* = 0.03).

The five nonsynonymous mutations unique to MD were from five different loci (i.e., AN-Pv8, AN-Pv18, AN-PvCO, AN-DNAJ, g523) and involved five MD accessions (Table 5). All of these gene fragments were re-sequenced for the five accessions involved, to confirm the presence of the mutations. The accessions from the Andean wild and domesticated populations and the North Peru and Ecuador population carried the same amino acids as MW and almost all of the MD accessions, as well as the closely related species *P. dumosus* and *P. coccineus*, this suggests that the five nonsynonymous mutations represent recent mutations that occurred after domestication in the MD germplasm.

**Table 5:** **Replacement changes unique to the MD population: locus, codon, corresponding amino acids, and number of accessions for the different populations and for the *P. dumosus* (Pd) and *P. coccineus* (Pc) controls for each of the five replacement changes unique to the MD population**.

**Locus**	**Codon**	**Aminoacid^a^**	**MW**	**MD^b^**	**AW**	**AD**	**PhI**	**Pd**	**Pc**
AN-Pv8	AAG	K	6	/	/	/	2	/	/
	AAA	K	13	19	2	2	/	1	1
	ACA	T	/	**1** (102)	/	/	/	/	/
AN-Pv18	ATG	M	19	18	2	2	2	1	1
	ACG	T	/	**2** (102,107)	/	/	/	/	/
AN-PvCO	CCA	P	15	14	2	2	2	1	1
	CTA	L		**3** (102,107,166)	/	/	/	/	/
AN-DNAJ	TCT	S	19	19	2	2	2	1	1
	TTT	F	/	**1** (89)	/	/	/	/	/
g523	TAT	Y	19	19	2	2	2	1	1
	TGT	C	/	**1** (104)	/	/	/	/	/

*In brackets: the code of the MD accessions carrying the specific nonsynonymous change*.

a *C, cysteine, F; phenylalanine; K, lysine; L, leucine; M, methionine; P, proline; S, serine; T, threonine; Y, tyrosine*.

b *Accession codes (see Table S1): **089**, PI151017; **102**, PI165440; **104**, PI196933; **107**, PI201349; **166**, PI313301*.

### Divergence and positive selection

The *P*-values for each gene are reported in Table S11. When Model 1 was used to generate the null distribution of the selection index, seven (17.9%; AN-Pv 22, AN-Pv26.1, AN-Pv33, AN-DNAJ, g523, Leg133, Leg223) out of the 39 genes analyzed had at least one exonic or intronic fragment identified as putatively selected during domestication in Mesoamerica. Three genes had selection signals only in exons, three only in introns, and one in both. The number of genes under putative selection increases to 11 (28.2%) under Model 2, as four with selection signals only in exons, five only in introns, and two in both. The set of genes identified under Model 1 is entirely included in the set of genes identified under Model 2. The four additional loci are AN-Pv64, AN-Pv69, AN-TGA, and PvSHP1.

**Figure 3:**
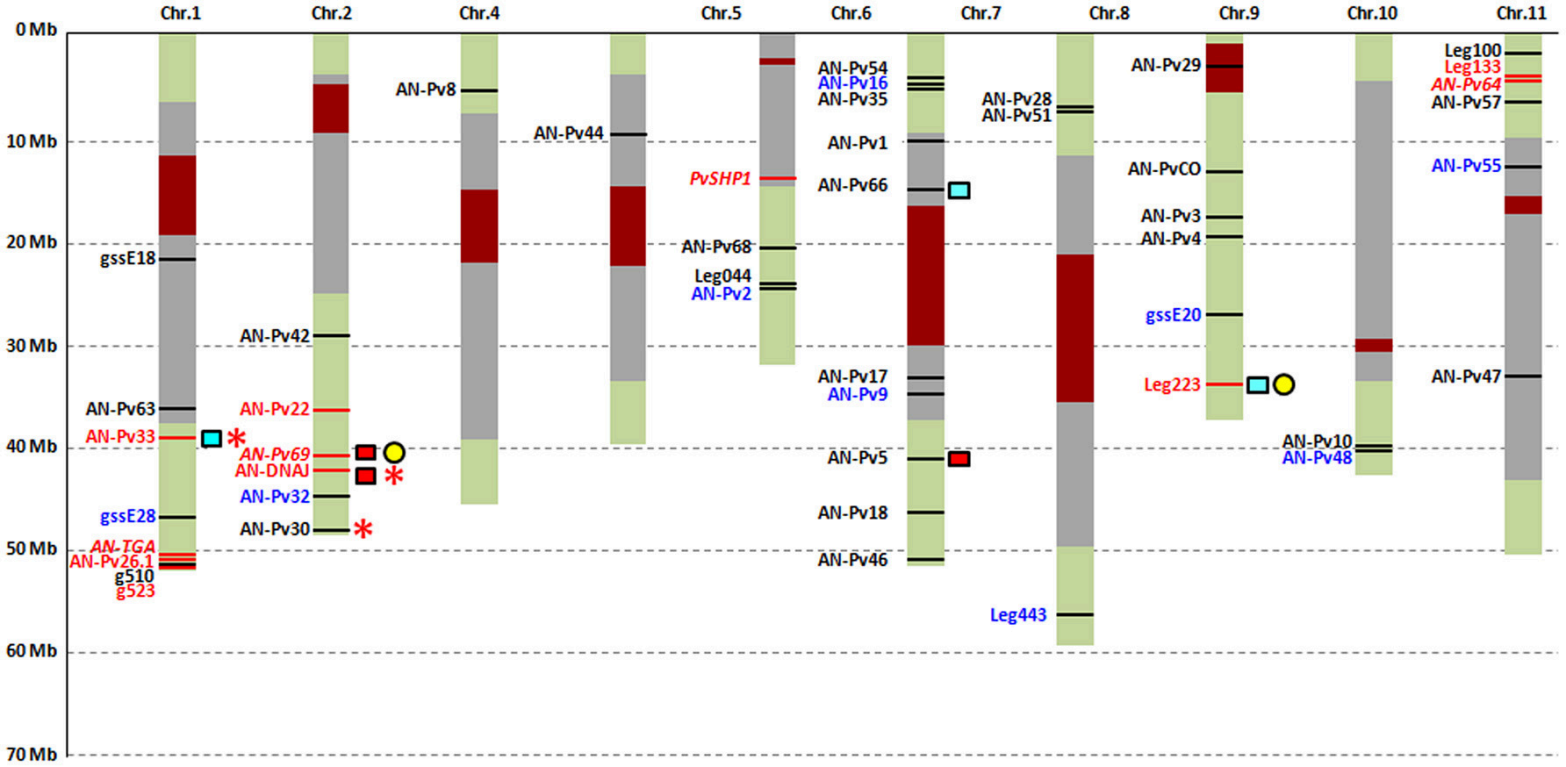
**Graphical representation of the results of the neutrality tests and comparison with other studies**. Blue, monomorphic (gssE20, AN-Pv9, AN-Pv32, AN-Pv55), or without structure information (gssE28, Leg443, AN-Pv48), or containing only 5′UTR (AN-Pv16) or 3′UTR (AN-Pv42) regions loci; red, loci detected as putatively under selection with Model 1; red and italic, the further four loci detected as putatively under selection with Model 2; black, putatively neutral loci; yellow circles, loci detected as putatively under selection in the Mesoamerican gene pool in Bellucci et al. (2014a); red and light blue squares, loci detected as putatively under selection in the Andean and Mesoamerican gene pool, respectively, by Schmutz et al. (2014); ^*^loci, including SNPs, detected as putatively under selection in the Mesoamerican gene pool using data from Rodriguez et al. (2016). Dark red and gray boxes, centromeric and pericentromeric regions, respectively.

To obtain supporting evidence for our findings of putatively selected loci that have been affected by selection during domestication (directly or due to hitchhiking), we compared our data with those of other studies for the same genomic regions. These comparisons are reported in Figure 3 and Table S12. For 25 out of the 42 loci (59.5%), we found a correspondence in the reference transcriptome (Table S13). In particular, the AN-Pv69 and Leg223 loci that were both putatively selected in our analyses matched with the Ref_259_comp14324 and Ref_25_comp4672 contigs, respectively (Bellucci et al., 2014a). These two contigs were also detected as under selection during common bean domestication in Mesoamerica in Bellucci et al. (2014a). The remaining 23 contigs that matched our loci did not show evidence of selection in Bellucci et al. (2014a).

Schmutz et al. (2014) investigated the domestication history of common bean by resequencing eight pools that comprised 160 wild and domesticated genotypes from both the Mesoamerican and Andean gene pools. They identified 10-kb windows with a 2-kb slide (10-kb/2-kb windows) and gene sequences as putatively under selection during common bean domestication. Eight loci found by Schmutz et al. (2014) in the 10-kb/2-kb windows showed putative selection during domestication in the Andean gene pool, one of which was identified as putatively selected in our study (AN-Pv22). Ten loci were present in 10-kb/2-kb windows detected as under selection in the Mesoamerican gene pool by Schmutz et al. (2014), four of which were identified as outliers (AN-Pv26.1, AN-Pv33, Leg133, and Leg223). Comparisons with candidate loci detected by Schmutz et al. (2014) that represent the same gene regions of the loci appeared to be more reliable than considering windows that encompass a large portion of the genome. We found six genes detected as outliers by Schmutz et al. (2014) that overlapped with those found in the present study. Three genes were detected as candidate genes in Mesoamerica, Pvul.001G143100, Pvul.007G113700, and Pvul.009G231900, which correspond to the AN-Pv33, AN-Pv66, and Leg223 loci, respectively, where AN-Pv33 and Leg223 were detected as putatively selected loci in our analysis. Similarly, in the Andean gene pool, three genes were detected as candidate genes, Phvul.007G177200, Phvul.002G242000, and Phvul.002G257300, which correspond to the AN-Pv5, AN-Pv69, and AN-DNAJ loci, respectively, with two of these detected as outliers in our study (AN-Pv69, AN-DNAJ); this is of interest, as this study was focused on domestication only in the Mesoamerican gene pool.

Finally, SNP data from a wider sample of 517 individuals (Rodriguez et al., 2016) have been investigated. Forty of the 131 SNPs used by Rodriguez et al. (2016) were located within 23 AN-Pv loci used in this study. We separated the selection analysis for the Andean and Mesoamerican gene pools. In each case, we excluded individuals considered to be potentially admixed between the two gene pools (Rodriguez et al., 2016).

For the Mesoamerican gene pool, we retained 408 out of the 417 genotyped individuals. Ninety-eight were domesticated, while 310 were wild. SNPs with more than 5% missing data and those with a frequency of minor allele ≤ 0.01 were not considered, which resulted in the loss of 19 SNPs and a final dataset of 112 SNPs. The filters allowed us to retain 31 SNPs in the analysis that were present in 18 of our AN-Pv loci (AN-Pv2, AN-Pv5, AN-Pv8, AN-Pv30, AN-Pv33, AN-Pv35, AN-Pv44, AN-Pv47, AN-Pv51, AN-Pv54, AN-Pv63, AN-Pv64, AN-Pv69, gssE18, AN-TGA, AN-PvCO, AN-DNAJ, and g510). Neutrality tests were performed by applying the Beaumont and Nichols (1996) approach, and these identified six SNPs putatively under selection during domestication of the common bean in Mesoamerica, five of which were designed by Goretti et al. (2014) and are present in the set of loci used in this study. In particular, the AN-DNAJ_105 and AN-DNAJ_246 SNPs were from the AN-DNAJ locus, the AN-Pv30_158 SNP from the AN-Pv30 locus, and the AN-Pv33_68 and AN-Pv33_136 SNPs from the AN-Pv33 locus. The SNP primers and the relative information are in Goretti et al. (2014). Interestingly, all five SNPs were in loci detected as putatively selected in the present study (Figure 3 and Table S12).

The same approach was used for the data of individuals from the Andean gene pool. There was a total of 133 Andean individuals, as 51 wild and 62 domesticated. From these, 61 SNPs were retained for the analysis, 13 of which were from 11 loci present in this study. In this analysis, 7 SNPs were detected as outliers; however, none of these SNPs were shared with the present study.

## Discussion

Overall our outcomes suggest that selection during domestication resulted in an increase in functional diversity at target loci, which appears to control traits related to expansion and adaptation to new agro-ecological growing conditions.

Consistent with the findings of Bellucci et al. (2014a), who analyzed the transcriptome (~27,000 genes) of a smaller set of MW and MD accessions, there was a severe reduction in genetic diversity (~57%) of the MD compared to the MW populations. However, an intriguing result was obtained by looking at the genomic changes at coding and noncoding regions separately, and considering both shared and unique polymorphisms between the wild and domesticated populations. Coding regions are expected to accumulate mutations slower than noncoding regions, because purifying selection acts on mutations that negatively alter gene function. In contrast, mutations in noncoding regions are less functionally constrained, and so they should accumulate more quickly (Li, 1997). In general, this is what we observed, in both the wild and domesticated populations. However, when we considered separately the polymorphisms in the shared and private substitutions in the wild and domesticated populations, we found a statistically supported exception: polymorphisms observed only in the domesticated plants occurred more frequently in the coding regions and implied several nonsynonymous changes. Furthermore, a significant increase in the difference between the average rate of nonsynonymous substitutions and synonymous substitutions in the domesticated compared to the wild forms was found.

How can the excess of nonsynonymous mutations within the domesticated germplasm be explained? This pattern of variation can result from neutral forces. The targeted gene fragments might be pseudogene sequences, for which mutations are expected to accumulate at a higher rate than coding sequences due to the loss of selective pressure (Li et al., 1981; Petrov and Hartl, 2000). Alternatively, a higher nonsynonymous mutation rate in the domesticated materials might be the result of genetic drift related to a reduction in the population size imposed by the domestication bottleneck that limited the purifying effect of deleterious mutations due to recombination (e.g., the cost of domestication). This can be enhanced in genomic regions that surround selected loci, due to genetic sweep. These phenomena might also have increased after the initial development of the domesticated pool, due to subsequent bottleneck episodes associated with crop expansion and breeding.

However, some results suggest that the cost of domestication hypothesis appears insufficient to fully explain these observations. Indeed, the inefficiency of purifying selection predicts an increase in the fraction of polymorphisms in coding regions, up to the level found in the noncoding regions. However, this cannot explain our observation that two thirds of the private polymorphisms that occurred in the domesticated gene pool are located within genes. Moreover, those mutations leading to changes in the amino-acid sequence appear in the domesticated populations at very low frequencies, as derived from five gene fragments. They are present only in a few of the MD genotypes (rare alleles), while *P. dumosus* and *P. coccineus* accessions, the wild population from North Peru and Ecuador, and the wild and domesticated populations from the Andes carry the same and more frequent alleles that are present in MW and in the majority of MD (Table 5). This outcome supports a scenario where new functional mutations that are probably not from standing variation were selected for adaptation during domestication. However, we cannot exclude that the development of local varieties also determined a “secondary domestication” cost, even if this would not explain the larger diversity that we observed for private mutations in the domesticated population for substitutions in coding regions. The gene-function investigation of the genes carrying the nonsynonymous changes and the genotypes carrying the mutated allele might also suggest that due to local adaptation, these mutations probably arose during expansion and diversification into new cultivated environments that were characterized by unexpected biotic and abiotic stresses. In particular, the AN-Pv8 gene is a β-glucan binding protein, and these proteins are known to be involved in pathogen recognition and the subsequent activation of disease resistance responses in plants (Fliegmann et al., 2005). This is seen, for example, in the β-glucan binding protein that recognizes the β-glucan elicitor that is released from the cell walls of the phytopathogenic oomycete *Phytophthora megasperma*, which results in defense reactions in soybean (Umemoto et al., 1997). AN-Pv18 and AN-DNAJ are known to be involved in plant tolerance/resistance to abiotic stress. In particular, AN-Pv18 is a late embryogenesis abundant protein, and high levels of these proteins accumulate in plants under environmental stress. In several studies, AN-Pv18 and AN-DNAJ have been shown to have roles in cellular protection during abiotic stress tolerance, and especially during drought (for review see Amara et al., 2014). AN-DNAJ belongs to the DNAJ heat-shock family of proteins that regulate the folding, localization, accumulation, and degradation of proteins, stabilize proteins and membranes, and can assist in protein refolding under stress conditions. Synthesis of these proteins was demonstrated to be induced in different plant species as a defense against several environmental stresses, such as heat, salinity, light, and heavy metals (Wang et al., 2004; Zhichang et al., 2010; Sun et al., 2012; Bekh-Ochir et al., 2013; Liu and Whitham, 2013). The AN-PvCO gene is homologous to the CONSTANS (CO) gene in *Arabidopsis* that is involved in flowering time (Putterill et al., 1995; Samach et al., 2000; Hayama and Coupland, 2004), which is a crucial trait for adaptation and crop expansion. Among the MD accessions that carried the functional mutations, PI 165440 carried three of the five nonsynonymous mutations that involve the AN-PV8, AN-Pv18, and AN-PvCO genes. Interestingly, this accession was collected in Mexico, Puebla State, at an altitude of 2430 m a.s.l., which is well above the altitudinal range of distribution of the MW common bean germplasm (Toro et al., 1990).

Our findings suggest that domestication not only had a major effect on the reduction of the genetic diversity at the genome level and at loci controlling useful features for human needs, but also acted to increase functional diversity at target loci that are probably involved in adaptation to different agro-ecological contexts. In this regard, our study supports the results of Bellucci et al. (2014a), who detected a small fraction (2.8%) of genes as under selection during common bean domestication in Mesoamerica. These are variable in the domesticated, while they are monomorphic in the wild population. One of these genes is a homolog of K^+^ uptake transporter6 (KUP6), which is involved in plant responses to water stress (Osakabe et al., 2013). This inference is consistent with the complex dynamics of the domestication process. This involves not only human selection acting on favorable traits, such as the shape and size of the edible parts, which have undergone the most impressive changes in distinguishing between the wild and domesticated forms, but also functional changes that are related to the spread and adaptation to different agro-ecological environments, which are sometimes referred to as diversification traits (for review see Meyer and Purugganan, 2013).

### Divergence and positive selection

By comparing the data obtained for the same loci/genes in different studies of varying sizes, data types, and methodologies (including incomplete overlap among gene fragments), we obtained independent evidence that some genes were targets of directional selection during common bean domestication. In particular, four (AN-Pv33, AN-Pv69, AN-DNAJ, and Leg223) out of the 11 loci that showed signatures of selection in the present study were detected as outliers in other studies (Bellucci et al., 2014a; Schmutz et al., 2014) or in re-analysis of the SNP data over a wide sample of Mesoamerican accessions (Rodriguez et al., 2016; Figure 3). These genes were found to be involved in plant resistance/tolerance to abiotic stresses, such as heat, drought, and salinity. In particular, AN-Pv33 encodes a late embryogenesis abundant protein and its involvement in plant reactions to abiotic stresses was described previously, as well as for the AN-DNAJ function. In general, both have a common function that is related to protection and stability of molecules and cellular structures during stress. Similarly, Leg223 appears to act to maintain cellular machinery processes during exposure of plants to abiotic stress conditions. This gene encodes a eukaryotic translation initiation factor SUI1 family protein that functions to ensure that there is a start codon, AUG, at the beginning of the protein, which helps to stabilize the pre-initiation complex, thus favoring protein translation and synthesis (Sonenberg and Hinnebusch, 2009). This gene is a homolog of elF-1, which has been demonstrated to increase salt tolerance in different plants, such as *Arabidopsis thaliana* (Rausell et al., 2003), rice (Diédhiou et al., 2008) and its salt-tolerant wild relative, *Porteresia coarctata* (Latha et al., 2004). Multiple and independent evidence for being under selection during domestication are available for AN-Pv69, which encodes a heat-shock transcription factor (HSF). By binding heat-stress elements in the target promoters of stress-inducible genes, the HSFs activate transcription of these genes, and thus have crucial roles in the mechanisms of plant responses to abiotic stress (Guo et al., 2016).

### Methodological issues

The data obtained in the present study and the possibility to compare these data with the outcomes of similar studies (Bellucci et al., 2014a; Schmutz et al., 2014; Rodriguez et al., 2016) has also given rise to several methodological considerations.

#### How important is the choice of the evolutionary model to be used when applying approaches based on coalescent simulations to detect signals of selection?

This question arises from the observed differences in the percentages of loci detected as under selection during domestication using the two different evolutionary models (Figure 1), with the percentage of outlier loci being 17.9 and 28.2% for Model I and Model II, respectively. The risk when applying coalescent approaches is to erroneously attribute to selection a poor fit to the true demographic history of a species (Siol et al., 2010). However, in the present study, the models were derived from the rich literature on evolution of the common bean, most of which focuses on domestication. Importantly, the two models did not give contrasting results; i.e., the genes identified as outliers in Model I were completely included in the set of outliers of Model II. This occurred also with Bellucci et al. (2014a), who applied the same approach to ~27,000 genes using three different evolutionary models, for which they obtained three nested sets of outliers. Working with a species for which there is a wealth of previous information makes it possible to build evolutionary models that can depart slightly from the true demographic history of the species. In this case, a better question to consider is how conservative do we want to be to reduce the false-positive rate of calling a locus an outlier when it is not? Not relying on a unique model, but performing the analysis using multiple models that mimic subtle differences in previous studies might be an empirical conservative solution. This was the strategy adopted by Bellucci et al. (2014a), who identified as outliers only genes that were detected under selection in all of the three models they considered.

#### Why is there such a small overlap for the fraction of genes studied here with the transcriptome (~27,000) analyzed in Bellucci et al. (2014a)?

Indeed, a relatively high fraction of genes sequenced in this study (41.5%) were not present in the reference transcriptome from Bellucci et al. (2014a). One explanation is related to the primer pairs used in the present study. These were designed to amplify a set of gene fragments homologous to soybean gene fragments (Zhu et al., 2003; Hyten et al., 2006) that were identified from gene sequences in GeneBank that were functionally annotated. Moreover, with the exception of the Leg markers (Hougaard et al., 2008; Bitocchi et al., 2012, 2013), the other 10 loci were selected as being near to domestication quantitative trait loci or were defined as putatively under selection during domestication in previous studies (Papa et al., 2005, 2007; Goretti et al., 2014). Thus, our set of loci cannot be completely considered as randomly chosen. Moreover, a rapid survey on the level of expression of most of the genes used in soybean and other legumes indicates that they are mostly expressed in roots and seeds; this could thus explain why most of them were not present in the leaf transcriptome used by Bellucci et al. (2014a).

#### Did domestication act in the same genomic regions or on completely different genes to obtain functionally convergent phenotypes?

One of the major controversies in evolutionary genomics is whether the effects of domestication at the genomic level were the same within and between species. The major goal is to determine whether convergent evolution was active not only at the phenotypic level (domestication syndrome), but also at the genomic level, with the selection of the same set of genes to obtain the same phenotypes in different species or gene pools. *P. vulgaris* represents an interesting model to answer this question (e.g., Bitocchi et al., 2013), because of the parallel and independent domestication that occurred in two different and geographically distinct gene pools. A first attempt to investigate convergent evolution in common bean was reported by Schmutz et al. (2014). They investigated the domestication history of common bean by resequencing pools representing genomes of 160 wild and cultivated genotypes from the two gene pools. They computed genetic diversity (π) and population differentiation (F_ST_) statistics using data averaged over 10-kb windows with a 2-kb slide (10-/2-kb windows), and using gene sequences. To indicate a 10-/2-kb window or a gene as a selection window or domestication candidate gene they considered whether it was in the upper 90% of the empirical distribution of the pool for the πwild/πlandrace ratio and F_ST_ statistics. Schmutz et al. (2014) found that <10% of the 74 Mb of sequence putatively involved in domestication, as well as only 3% of the Mesoamerican and 8% of the Andean candidates, were shared by the two domestication events. Their conclusion was that the Mesoamerican and Andean lineages underwent independent selection upon distinct sets of genes, while they evolved similar morphologies and life cycles. What we see from the present study is that two (AN-Pv69, AN-DNAJ) out of the four strong candidates detected under selection during Mesoamerican domestication were identified by Schmutz et al. (2014) as under selection only during Andean domestication. This suggests that more studies and evidence are needed to either support or refute the lack of correlations between the two gene pools found by Schmutz et al. (2014). A potential drawback of the analysis carried out by Schmutz et al. (2014) is that they did not use an explicit demographic model to generate an expectation of the number of potential false-positive regions, and thus they potentially identified a high level of false positives (i.e., regions of the genome with reduced diversity due to stochastic effects of domestication bottlenecks). Moreover, when approaching such an issue, it has to be taken into account that the two gene pools have different demographic histories, with two sequential bottlenecks that occurred in the Andes, one prior to domestication and the other during domestication. This strongly impoverished the genetic diversity present in the Andean gene pool compared to the Mesoamerican gene pool.

## Conclusions

We have shown independent evidence of positive selection due to domestication on four genes, along with an increase in the functional diversity at five genes in domesticated germplasm. The gene function surveys for these genes suggest that they are involved in adaptation outside the geographic distribution of the wild germplasm, and in particular, in plant responses to biotic and abiotic stresses.

The present study also opens a debate on the fascinating issue of convergent evolution. In particular, we found some discrepancies between our data and those of Schmutz et al. (2014), a situation that suggests that more studies are needed to obtain a more detailed picture as to how evolution acted in promoting the same phenotypic changes in these domesticated plants.

## Author contributions

RP and EBit conceived and designed the research. DG, EBel, and EBia performed the DNA extraction and sequencing of gene fragments. EBit, DG, and MR performed sequence alignment and editing of the sequences. GB, AB, and AP performed coalescence simulations and GB, AB, AP, and EBit carried out selection detection. EBit, DR, TG, GA, and GL performed population genetics analysis. PM, RL, SJ, and EBia mapped the gene fragments on reference genome and performed BLAST analysis to search for matches with sequences from other studies; EBia and DR performed gene function investigation. RP and EBit wrote the manuscript and DR and SJ contributed to the drafting and writing of the article. All authors read and approved the article.

## Funding

This work was supported by grants from the Italian Government (MIUR; Grant number RBFR13IDFM_001, FIRB Project 2013) and by Università Politecnica delle Marche (years 2014-2015).

### Conflict of interest statement

The authors declare that the research was conducted in the absence of any commercial or financial relationships that could be construed as a potential conflict of interest.
