# Supplementary material for: High Level of Nonsynonymous Changes in Common Bean Suggests That Selection under Domestication Increased Functional Diversity at Target Traits
Source: Front Plant Sci. 2017 Jan 6;7:2005. doi: 10.3389/fpls.2016.02005 (PMC5216878; doi:10.3389/fpls.2016.02005)
Supplement: Supplementary file 3 [file Table3.PDF]

Table S3. Demographic parameters for Models 1 and 2.

Model	Parameter	Description	Distribution	Mean	S.D.	Min	Max
Model 1	T_{ANC}	Divergence time between Andean and Mesoamerican gene pools	Normal	111,000	40,000	67,330	192,835
	T_{BAB}	Time of the beginning of the Andean founder bottleneck	Normal	98,845	3,000	94,051	104,027
	T_{EAB}	Time of the ending of the Andean founder bottleneck	Normal	67,858	1,500	64,655	70,865
	T_{BMD}	Time of the beginning of the Mesoamerican domestication	Normal	8,160	133	7,922	8,426
	T_{BAD}	Time of the beginning of the Andean domestication	Normal	8,500	8	8,495	8,517
	T_{EMD}	Time of the ending of the Mesoamerican domestication	Normal	6,260	150	5,971	6,567
	T_{EAD}	Time of the ending of the Andean domestication	Normal	7,012	35	6,945	7,075
	N_{ANC}	Ancestral effective population size	Normal	418,000	105,000	266,000	628,000
	N_{BA}	Andean effective population size during the founder bottleneck	Normal	105,000	20,000	65,000	142,000
	N_{MD}	Effective population size of the domesticated Mesoamerican population	Uniform			100,000	100,000
	N_{MW}	Effective population size of the wild Mesoamerican population	Normal	292,000	240,000	125,000	773,000
	N_{AW}	Effective population size of the wild Andean population	Normal	137,000	182,000	70,000	502,000
	N_{AD}	Effective population size of the domesticated Andean population	Uniform			100,000	100,000
	I_{MD}	Intensity of the domestication bottleneck in Mesoamerica (in percentage)	Normal	47.65	3	41.66	52.13
	I_{AD}	Intensity of the domestication bottleneck in the Andes (in percentage)	Normal	47.26	0.5	46.25	48.59
	M_{WD}	Migration rate from wild to domesticated population	Uniform			0.000001	0.00001
	XM	Asymmetric migration factor	Uniform			2	6
	M_{DW}	Migration rate from domesticated to wild population	XM*MWD				
	MU	Mutation rate (per site per generation)	Normal	$1E^{-9}$	$5E^{-9}$	$1E^{-10}$	$1E^{-8}$
	L	Length of the simulated contig	Normal	1,300	1,500	250	5,000
Model 2	N_{MW}	Effective population size of the wild Mesoamerican population	Normal	561,000	50,000	463,300	658,300
	N_{MD}	Effective population size of the domesticated Mesoamerican population	Uniform			100,000	100,000
	N_{AW}	Effective population size of the wild Andean population	Normal	219,000	25,000	188,500	271,300
	N_{AD}	Effective population size of the domesticated Andean population	Uniform			100000	100000
	M_{DW}	Migration rate from domesticated to wild population	XM*MWD				
	M_{WD}	Migration rate from wild to domesticated population	Uniform			0,000001	0,00001
	M_{MWAW}	Migration rate from wild Mesoamerican to Andean wild population	Normal	0,0000004	0,000000026	0,000000357	0,000000452

M_{AWMW}	Migration rate from wild Andean to Mesoamerican wild population	Normal	0,00000026	0,000000023	0,000000214	0,000000297
T_{EMD}	Time of the ending of the Mesoamerican domestication	Normal	6,260	150	5,971	6,567
T_{EAD}	Time of the ending of the Andean domestication	Normal	7,012	35	6,945	7,075
T_{BMD}	Time of the beginning of the Mesoamerican domestication	Normal	8,160	133	7,922	8,426
T_{BAD}	Time of the beginning of the Andean domestication	Normal	8,500	8	8,495	8,517
T_{EAB}	Time of the ending of the Andean founder bottleneck	TANC-DURBOTWA				
T_{ANC}	Divergence time between Andean and Mesoamerican gene pools	Normal	165,000	10,000	146,200	183,700
MU	Mutation rate (per site per generation) (exon)	Lognorm	$1E^{-9}$	$2,5E^{-9}$	$5E^{-10}$	$5E^{-9}$
MU	Mutation rate (per site per generation) (intron)	Lognorm	$1E^{-8}$	$2,5E^{-8}$	$5E^{-9}$	$5E^{-8}$
I_{MD}	Intensity of the domestication bottleneck in Mesoamerica (in percentage)	Normal	47.65	3	41.66	52.13
I_{AD}	Intensity of the domestication bottleneck in the Andes (in percentage)	Normal	47.26	0.5	46.25	48.59
XM	Asymmetric migration factor	Uniform			2	6
N_{ANC}	Ancestral effective population size	Normal	168,000	5,000	158,900	176,200
N_{MWANC}	Ancestral wild Mesoamerican effective population size	Normal	155,000	25,000	124,900	205,800
N_{AWANC}	Ancestral Andean mesoamerican effective population size	Normal	3,590	2,750	2,304	8,978
LAB	Length of the Andean founder bottleneck	Normal	75,900	12,000	60,370	99,470
