# Supplementary material for: High Level of Nonsynonymous Changes in Common Bean Suggests That Selection under Domestication Increased Functional Diversity at Target Traits
Source: Front Plant Sci. 2017 Jan 6;7:2005. doi: 10.3389/fpls.2016.02005 (PMC5216878; doi:10.3389/fpls.2016.02005)
Supplement: Supplementary file 4 [file Table4.PDF]

Table S4. Genetic diversity estimates for each of the 49 loci (whole sequence length), computed considering the **a)** *P. vulgaris* sample, **b)** Mesoamerican accessions of *P. vulgaris*, **c)** Mesoamerican wild (MW) and **d)** domesticated (MD) accessions.

		**Locus**	**N**	**Range bp**	**V**	**η**	**S**	**Pi**	**H**	**Hd**	**$\pi \times 10^{-3}$**	**$\Theta \times 10^{-3}$**
P. vulgaris sample	1	AN-Pv1	45	240 - 242	6	6	3	3	4	0.28	2.88	5.72
	2	AN-Pv2	45	418	5	5	0	5	5	0.55	4.54	2.74
	3	AN-Pv3	45	373 - 379	7	7	5	2	4	0.41	1.68	4.29
	4	AN-Pv4	45	421 - 452	5	5	2	3	4	0.45	1.47	2.73
	5	AN-Pv5	45	486	3	3	1	2	3	0.28	1.06	1.41
	6	AN-Pv8	44	386	7	7	1	6	7	0.60	4.07	4.17
	7	AN-Pv9	45	411 - 415	0	0	0	0	1	0.00	0.00	0.00
	8	AN-Pv10	45	343 - 344	8	8	1	7	6	0.69	6.52	5.33
	9	AN-Pv16	43	296 - 297	8	8	1	7	9	0.60	9.85	6.25
	10	AN-Pv17	45	334 - 336	2	2	1	1	3	0.17	0.51	1.37
	11	AN-Pv18	45	408	10	10	0	10	3	0.21	3.02	5.61
	12	AN-Pv22	43	444	12	12	5	7	12	0.85	6.73	6.25
	13	AN-Pv26.1	45	678 - 683	15	16	3	12	12	0.78	7.31	5.06
	14	AN-Pv28	45	323 - 331	7	7	1	6	6	0.69	8.93	4.97
	15	AN-Pv29	45	245	7	7	0	7	6	0.46	4.61	6.53
	16	AN-Pv30	45	215	4	4	0	4	4	0.67	7.65	4.25
	17	AN-Pv32	45	282	5	5	1	4	3	0.21	2.51	4.05
	18	AN-Pv33	45	224	4	4	0	4	4	0.64	4.99	4.08
	19	AN-Pv35	45	209	2	2	0	2	2	0.09	0.83	2.19
	20	AN-Pv41	45	133 - 150	3	3	1	2	4	0.28	2.23	5.16
	21	AN-Pv42	45	185 - 186	5	5	1	4	4	0.47	6.87	6.21
	22	AN-Pv44	45	451	5	5	0	5	5	0.39	2.62	2.54
	23	AN-Pv46	45	498 - 511	4	4	1	3	6	0.73	2.86	1.84
	24	AN-Pv47	45	537 - 538	11	11	1	10	8	0.77	7.30	4.68
	25	AN-Pv48	39	482 - 496	21	21	1	20	10	0.86	14.53	10.37

	26	AN-Pv51	43	700	20	20	4	16	8	0.76	5.60	6.60
	27	AN-Pv54	42	398	9	9	3	6	8	0.81	5.92	5.26
	28	AN-Pv55	45	601	0	0	0	0	1	0.00	0.00	0.00
	29	AN-Pv57	45	457	12	13	2	10	6	0.58	7.48	6.00
	30	AN-Pv63	45	600	8	8	1	7	8	0.81	4.72	3.05
	31	AN-Pv64	45	537 - 589	10	10	0	10	7	0.52	2.96	4.26
	32	AN-Pv66	45	265	4	4	0	4	3	0.21	1.77	3.45
	33	AN-Pv68	44	717 - 728	24	25	7	17	10	0.72	6.71	7.69
	34	AN-Pv69	45	669 - 670	3	3	0	3	4	0.61	1.40	1.03
	35	gssE18	45	257	9	9	3	6	7	0.70	9.20	8.01
	36	gssE19	45	251	3	3	1	2	4	0.25	1.46	2.73
	37	gssE20	45	277 - 280	1	1	0	1	2	0.09	0.31	0.83
	38	gssE28	45	261 - 262	10	11	0	10	5	0.36	5.78	8.76
	39	AN-*PvCO*	38	651 - 655	18	18	2	16	9	0.76	7.17	6.60
	40	AN-*TGA*	42	580 - 607	15	15	3	12	8	0.80	5.10	6.01
	41	AN-*DNAJ*	45	597 - 597	8	8	3	5	6	0.61	3.79	3.06
	42	g510	45	559 - 565	10	10	1	9	7	0.70	5.66	4.11
	43	g523	45	482	3	3	1	2	3	0.53	2.20	1.42
	44	Leg044	44	866 - 867	24	24	1	23	9	0.79	6.65	6.70
	45	Leg100	45	599 - 713	42	44	2	40	9	0.83	24.07	17.03
	46	Leg133	45	584 - 590	12	13	0	12	5	0.67	5.56	4.70
	47	Leg223	41	471 - 505	7	7	1	6	8	0.71	2.90	3.51
	48	Leg443	40	238	5	5	2	3	6	0.55	3.99	4.94
	49	PvSHP1	45	875 - 889	42	45	9	33	17	0.79	10.17	11.19
		**Mean**	**44.2**	**439.1-446.8**	**9.5**	**9.7**	**1.6**	**7.9**	**6.0**	**0.54**	**5.02**	**4.79**
		**Overall**	**/**	**21,514-21,894**	**465**	**475**	**76**	**389**	**/**	**/**	**/**	**/**
Mesoamerican accessions of *P. vulgaris*	1	AN-Pv1	39	240 - 242	6	6	3	3	4	0.24	2.62	5.91
	2	AN-Pv2	39	418	5	5	0	5	5	0.44	3.52	2.83
	3	AN-Pv3	39	379	7	7	5	2	4	0.46	1.89	4.37
	4	AN-Pv4	39	421 - 452	3	3	2	1	3	0.43	1.18	1.69
	5	AN-Pv5	39	486	3	3	1	2	3	0.15	0.52	1.46
	6	AN-Pv8	38	386	7	7	1	6	6	0.61	4.24	4.32
	7	AN-Pv9	39	411 - 415	0	0	0	0	1	0.00	0.00	0.00
	8	AN-Pv10	39	343 - 344	8	8	1	7	6	0.68	6.55	5.52

9	AN-Pv16	37	296 - 297	7	7	1	6	8	0.47	4.36	5.66
10	AN-Pv17	39	336	1	1	0	1	2	0.15	0.43	0.70
11	AN-Pv18	39	408	10	10	9	1	3	0.15	1.38	5.80
12	AN-Pv22	37	444	11	11	4	7	10	0.80	6.78	5.93
13	AN-Pv26.1	39	678 - 683	15	16	3	12	11	0.73	7.13	5.23
14	AN-Pv28	39	323 - 331	6	6	0	6	5	0.63	8.46	4.41
15	AN-Pv29	39	245	5	5	0	5	5	0.44	4.14	4.83
16	AN-Pv30	39	215	4	4	0	4	4	0.65	7.85	4.40
17	AN-Pv32	39	282	0	0	0	0	1	0.00	0.00	0.00
18	AN-Pv33	39	224	4	4	1	3	4	0.55	3.58	4.22
19	AN-Pv35	39	209	2	2	0	2	2	0.10	0.96	2.26
20	AN-Pv41	39	133 - 150	3	3	1	2	4	0.24	1.89	5.34
21	AN-Pv42	39	185 - 186	5	5	1	4	4	0.52	7.60	6.43
22	AN-Pv44	39	451	4	4	1	3	4	0.20	1.18	2.10
23	AN-Pv46	39	498 - 511	4	4	1	3	6	0.69	2.69	1.91
24	AN-Pv47	39	537 - 538	11	11	1	10	7	0.74	7.70	4.84
25	AN-Pv48	34	482 - 496	21	21	1	20	10	0.87	15.64	10.72
26	AN-Pv51	37	700	19	19	3	16	7	0.71	5.92	6.50
27	AN-Pv54	36	398	8	8	3	5	7	0.77	6.00	4.85
28	AN-Pv55	39	601	0	0	0	0	1	0.00	0.00	0.00
29	AN-Pv57	39	457	12	13	2	10	6	0.52	6.44	6.21
30	AN-Pv63	39	600	8	8	3	5	7	0.76	4.27	3.15
31	AN-Pv64	39	537 - 589	8	8	4	4	5	0.40	2.10	3.52
32	AN-Pv66	39	265	4	4	3	1	3	0.15	0.96	3.57
33	AN-Pv68	39	727 - 728	18	18	8	10	8	0.65	3.82	5.86
34	AN-Pv69	39	669 - 670	2	2	0	2	3	0.53	1.05	0.71
35	gssE18	39	257	8	8	2	6	6	0.66	8.06	7.36
36	gssE19	39	251	2	2	1	1	3	0.15	0.78	1.88
37	gssE20	39	277 - 280	0	0	0	0	1	0.00	0.00	0.00
38	gssE28	39	261 - 262	10	10	0	10	4	0.28	5.19	9.06
39	AN-*PvCO*	32	651 - 655	17	17	1	16	8	0.70	6.83	6.50
40	AN-*TGA*	36	580 - 581	14	14	3	11	7	0.77	5.17	5.82
41	AN-*DNAJ*	39	597	7	7	2	5	5	0.53	3.38	2.77
42	g510	39	559 - 565	10	10	1	9	6	0.65	4.86	4.23

	43	g523	39	482	3	3	1	2	3	0.53	2.17	1.47
	44	Leg044	38	866 - 867	22	22	12	10	7	0.73	5.09	6.05
	45	Leg100	39	599 - 713	39	41	2	37	8	0.81	21.82	16.33
	46	Leg133	39	584 - 590	12	13	0	12	5	0.60	5.09	4.86
	47	Leg223	36	471 - 505	6	6	1	5	7	0.64	2.33	3.10
	48	Leg443	36	238	5	5	2	3	5	0.47	3.21	5.07
	49	PvSHP1	39	875 - 886	39	41	9	30	15	0.73	9.82	10.66
		**Mean**	**38.3**	**439.4-446.2**	**8.7**	**8.8**	**2.0**	**6.6**	**5.3**	**0.48**	**4.42**	**4.50**
		**Overall**	**/**	**21,532-21,865**	**425**	**432**	**100**	**325**	**/**	**/**	**/**	**/**
Mesoamerican wild (MW) accessions of *P. vulgaris*	1	AN-Pv1	19	240 - 242	6	6	3	3	4	0.45	5.02	7.15
	2	AN-Pv2	19	418	5	5	1	4	4	0.51	4.14	3.42
	3	AN-Pv3	19	379	7	7	5	2	4	0.61	2.99	5.28
	4	AN-Pv4	19	421 - 452	3	3	2	1	3	0.57	1.73	2.05
	5	AN-Pv5	19	486	3	3	1	2	3	0.29	1.03	1.77
	6	AN-Pv8	19	386	6	6	1	5	5	0.76	6.18	4.45
	7	AN-Pv9	19	411 - 415	0	0	0	0	1	0.00	0.00	0.00
	8	AN-Pv10	19	343 - 344	8	8	2	6	6	0.74	6.89	6.67
	9	AN-Pv16	17	296 - 297	7	7	1	6	8	0.82	7.45	7.00
	10	AN-Pv17	19	336	1	1	0	1	2	0.28	0.84	0.85
	11	AN-Pv18	19	408	9	9	9	0	2	0.11	2.32	6.31
	12	AN-Pv22	18	444	11	11	4	7	8	0.70	6.05	7.20
	13	AN-Pv26.1	19	678 - 683	15	16	6	9	11	0.88	5.70	6.33
	14	AN-Pv28	19	323 - 331	6	6	0	6	5	0.74	9.04	5.33
	15	AN-Pv29	19	245	4	4	0	4	4	0.46	5.35	4.67
	16	AN-Pv30	19	215	4	4	0	4	4	0.68	7.83	5.32
	17	AN-Pv32	19	282	0	0	0	0	1	0.00	0.00	0.00
	18	AN-Pv33	19	224	4	4	1	3	4	0.63	5.59	5.11
	19	AN-Pv35	19	209	2	2	0	2	2	0.20	1.90	2.74
	20	AN-Pv41	19	133 - 150	3	3	2	1	4	0.38	3.08	6.45
	21	AN-Pv42	19	185 - 186	5	5	1	4	4	0.66	9.66	7.77
	22	AN-Pv44	19	451	3	3	0	3	3	0.29	1.50	1.90
	23	AN-Pv46	19	498 - 511	4	4	1	3	6	0.80	2.74	2.31
	24	AN-Pv47	19	537 - 538	11	11	1	10	6	0.83	6.66	5.86
	25	AN-Pv48	17	482 - 496	19	19	2	17	8	0.90	13.72	11.73

	26	AN-Pv51	17	700	19	19	12	7	7	0.87	4.89	8.03
	27	AN-Pv54	16	398	6	6	1	5	4	0.73	4.84	4.54
	28	AN-Pv55	19	601	0	0	0	0	1	0.00	0.00	0.00
	29	AN-Pv57	19	457	12	13	6	6	6	0.54	6.14	7.51
	30	AN-Pv63	19	600	8	8	3	5	7	0.78	4.37	3.81
	31	AN-Pv64	19	537 - 589	8	8	4	4	5	0.70	3.66	4.26
	32	AN-Pv66	19	265	4	4	3	1	3	0.29	1.94	4.32
	33	AN-Pv68	19	727 - 728	17	17	9	8	6	0.71	4.68	6.69
	34	AN-Pv69	19	669 - 670	2	2	0	2	3	0.67	1.22	0.86
	35	gssE18	19	257	7	7	1	6	5	0.77	11.10	7.79
	36	gssE19	19	251	2	2	1	1	3	0.29	1.54	2.28
	37	gssE20	19	277 - 280	0	0	0	0	1	0.00	0.00	0.00
	38	gssE28	19	261 - 262	10	10	0	10	3	0.37	7.62	10.96
	39	AN-*PvCO*	15	651 - 655	16	16	4	12	7	0.78	8.72	7.58
	40	AN-*TGA*	19	580 - 581	14	14	4	10	6	0.78	8.03	6.91
	41	AN-*DNAJ*	19	597	6	6	1	5	4	0.71	4.41	2.88
	42	g510	19	559 - 565	10	10	3	7	6	0.80	6.13	5.12
	43	g523	19	482	2	2	0	2	2	0.46	1.89	1.19
	44	Leg044	18	866 - 867	22	22	13	9	6	0.81	6.18	7.39
	45	Leg100	19	599 - 713	38	40	2	36	7	0.88	27.53	19.24
	46	Leg133	19	584 - 590	12	13	0	12	5	0.81	7.83	5.88
	47	Leg223	17	471 - 505	6	6	1	5	7	0.87	3.91	3.81
	48	Leg443	17	238	5	5	2	3	5	0.64	4.88	6.21
	49	PvSHP1	19	875 - 886	39	41	10	29	14	0.97	15.06	12.91
		**Mean**	**18.6**	**439.4-446.2**	**8.4**	**8.5**	**2.5**	**5.9**	**4.8**	**0.58**	**5.39**	**5.26**
		**Overall**	**/**	**21,532-21,865**	**411**	**418**	**123**	**288**	**/**	**/**	**/**	**/**
Mesoamerican domesticated (MD) accessions of *P. vulgaris*	1	AN-Pv1	20	240 - 242	0	0	0	0	1	0.00	0.00	0.00
	2	AN-Pv2	20	418	4	4	0	4	3	0.35	2.90	2.70
	3	AN-Pv3	20	379	1	1	0	1	2	0.27	0.71	0.74
	4	AN-Pv4	20	421 - 452	1	1	0	1	2	0.19	0.45	0.67
	5	AN-Pv5	20	486	0	0	0	0	1	0.00	0.00	0.00
	6	AN-Pv8	19	386	3	3	1	2	3	0.29	1.30	2.22
	7	AN-Pv9	20	411 - 415	0	0	0	0	1	0.00	0.00	0.00
	8	AN-Pv10	20	343 - 344	5	5	0	5	3	0.62	6.12	4.11

9	AN-Pv16	20	296 - 297	0	0	0	0	1	0.00	0.00	0.00
10	AN-Pv17	20	336	0	0	0	0	1	0.00	0.00	0.00
11	AN-Pv18	20	408	1	1	0	1	2	0.19	0.46	0.69
12	AN-Pv22	19	444	4	4	0	4	4	0.58	3.40	2.58
13	AN-Pv26.1	20	678 - 683	9	9	6	3	3	0.20	1.72	3.73
14	AN-Pv28	20	323 - 331	5	5	0	5	2	0.51	7.85	4.38
15	AN-Pv29	20	245	4	4	3	1	3	0.42	2.60	4.60
16	AN-Pv30	20	215	3	3	0	3	2	0.44	6.17	3.93
17	AN-Pv32	20	282	0	0	0	0	1	0.00	0.00	0.00
18	AN-Pv33	20	224	0	0	0	0	1	0.00	0.00	0.00
19	AN-Pv35	20	209	0	0	0	0	1	0.00	0.00	0.00
20	AN-Pv41	20	133 - 150	1	1	1	0	2	0.10	0.75	2.12
21	AN-Pv42	20	185 - 186	3	3	0	3	2	0.34	5.49	4.60
22	AN-Pv44	20	451	4	4	4	0	2	0.10	0.89	2.50
23	AN-Pv46	20	498 - 511	3	3	1	2	3	0.47	1.89	1.70
24	AN-Pv47	20	537 - 538	8	8	0	8	4	0.55	6.65	4.20
25	AN-Pv48	17	482 - 496	19	19	1	18	6	0.83	17.38	11.73
26	AN-Pv51	20	700	12	12	1	11	3	0.47	6.35	4.83
27	AN-Pv54	20	398	7	7	2	5	6	0.76	6.76	4.96
28	AN-Pv55	20	601	0	0	0	0	1	0.00	0.00	0.00
29	AN-Pv57	20	457	10	11	4	6	3	0.51	6.91	6.17
30	AN-Pv63	20	600	5	5	0	5	2	0.40	3.29	2.35
31	AN-Pv64	20	537 - 589	0	0	0	0	1	0.00	0.00	0.00
32	AN-Pv66	20	265	0	0	0	0	1	0.00	0.00	0.00
33	AN-Pv68	20	727 - 728	9	9	2	7	4	0.60	3.10	3.49
34	AN-Pv69	20	669 - 670	0	0	0	0	1	0.00	0.00	0.00
35	gssE18	20	257	5	5	1	4	3	0.20	3.34	5.48
36	gssE19	20	251	0	0	0	0	1	0.00	0.00	0.00
37	gssE20	20	277 - 280	0	0	0	0	1	0.00	0.00	0.00
38	gssE28	20	261 - 262	4	4	0	4	2	0.19	2.90	4.32
39	AN-*PvCO*	17	651 - 655	12	12	3	9	4	0.63	4.83	5.47
40	AN-*TGA*	17	580 - 581	3	3	3	0	3	0.23	0.61	1.53
41	AN-*DNAJ*	20	597	4	4	4	0	3	0.20	0.67	1.89
42	g510	20	559 - 565	8	8	5	3	3	0.28	2.19	4.03

43	g523	20	482	3	3	1	2	3	0.35	1.32	1.75
44	Leg044	20	866 - 867	10	10	1	9	4	0.64	4.22	3.25
45	Leg100	20	599 - 713	23	23	0	23	3	0.63	14.57	11.47
46	Leg133	20	584 - 590	1	1	0	1	2	0.19	0.32	0.48
47	Leg223	19	471 - 505	0	0	0	0	1	0.00	0.00	0.00
48	Leg443	19	238	3	3	2	1	4	0.29	1.72	3.61
49	PvSHP1	20	875 - 886	21	21	21	0	3	0.20	2.43	6.85
	**Mean**	**19.7**	**439.4-446.2**	**4.4**	**4.5**	**1.4**	**3.1**	**2.4**	**0.27**	**2.70**	**2.64**
	**Overall**	**/**	**21,532-21,865**	**218**	**219**	**67**	**151**	**/**	**/**	**/**	**/**

N, sample size; Range bp, sequence length (base pairs); *V*, variable sites; *η*, total number of mutations; *S*, singleton variable sites; *Pi*, parsimony informative variable sites; *H*, number of haplotypes; *Hd*, haplotype diversity; $\pi \times 10^{-3}$ and $\Theta \times 10^{-3}$, two measure of nucleotide diversity from Tajima (1983) and Watterson (1975), respectively; na, not applicable
