# Supplementary material for: High Level of Nonsynonymous Changes in Common Bean Suggests That Selection under Domestication Increased Functional Diversity at Target Traits
Source: Front Plant Sci. 2017 Jan 6;7:2005. doi: 10.3389/fpls.2016.02005 (PMC5216878; doi:10.3389/fpls.2016.02005)
Supplement: Supplementary file 5 [file Table5.PDF]

Table S5. Genetic diversity estimates computed considering the coding regions of the 42 loci characterized and including exons for the **a)** *P. vulgaris* sample, **b)** Mesoamerican accessions of *P. vulgaris*, **c)** Mesoamerican wild (MW) and **d)** domesticated (MD) accessions.

		Locus	N	Range bp	V	η	S	Pi	Syn	Nonsyn	H	Hd	π x 10^{-3}	Θ x 10^{-3}
P. vulgaris sample	1	AN-Pv1	45	229	5	5	3	2	2	3	4	0.28	1.84	4.99
	2	AN-Pv2	45	351	1	1	0	1	1	0	2	0.46	1.30	0.65
	3	AN-Pv3	45	233	4	4	2	2	1	3	4	0.41	2.12	3.93
	4	AN-Pv4	45	258	0	0	0	0	0	0	1	0.00	0.00	0.00
	5	AN-Pv5	45	258	0	0	0	0	0	0	1	0.00	0.00	0.00
	6	AN-Pv8	44	386	7	7	1	6	5	2	7	0.60	4.07	4.17
	7	AN-Pv9	45	232	0	0	0	0	0	0	1	0.00	0.00	0.00
	8	AN-Pv10	45	213	4	4	0	4	2	2	3	0.63	8.18	4.29
	9	AN-Pv17	45	114	1	1	1	0	1	0	2	0.04	0.39	2.01
	10	AN-Pv18	45	408	10	10	0	10	4	6	3	0.21	3.02	5.61
	11	AN-Pv22	43	339	8	8	4	4	6	2	11	0.84	5.70	5.45
	12	AN-Pv26.1	45	204	6	7	2	4	4	3	9	0.67	8.83	6.73
	13	AN-Pv28	45	87	1	1	0	1	1	0	2	0.51	5.81	2.63
	14	AN-Pv29	45	78	2	2	0	2	0	2	3	0.31	4.95	5.86
	15	AN-Pv30	45	215	4	4	0	4	3	1	4	0.67	7.65	4.25
	16	AN-Pv32	45	137	0	0	0	0	0	0	1	0.00	0.00	0.00
	17	AN-Pv33	45	224	4	4	0	4	3	1	4	0.64	4.99	4.08
	18	AN-Pv35	45	45	0	0	0	0	0	0	1	0.00	0.00	0.00
	19	AN-Pv44	45	343	5	5	0	5	4	1	5	0.39	3.45	3.33
	20	AN-Pv46	45	380	4	4	1	3	0	4	6	0.73	3.74	2.41
	21	AN-Pv47	45	438	6	6	1	5	4	2	7	0.76	4.36	3.13
	22	AN-Pv51	43	318	8	8	2	6	8	0	5	0.55	5.34	5.81
	23	AN-Pv54	42	398	9	9	3	6	9	0	8	0.81	5.92	5.26
	24	AN-Pv55	45	390	0	0	0	0	0	0	1	0.00	0.00	0.00
	25	AN-Pv57	45	241	5	5	2	3	5	0	4	0.31	3.31	4.74
	26	AN-Pv63	45	600	8	8	1	7	4	4	8	0.81	4.72	3.05
	27	AN-Pv64	45	229	1	1	0	1	1	0	2	0.13	0.56	1.00
	28	AN-Pv66	45	265	4	4	0	4	0	3	3	0.21	1.77	3.45
	29	AN-Pv68	44	522	10	11	1	9	6	5	9	0.71	4.46	4.40

	30	AN-Pv69	45	273	1	1	0	1	1	0	2	0.35	1.30	0.84
	31	gssE18	45	70	3	3	1	2	1	2	3	0.48	14.03	9.80
	32	gssE20	45	80	0	0	0	0	0	0	1	0.00	0.00	0.00
	33	AN-PvCO	38	538	9	9	2	7	4	4	6	0.72	4.50	3.98
	34	AN-TGA	42	172	1	1	1	0	1	0	2	0.05	0.28	1.35
	35	AN-DNAJ	45	597	8	8	3	5	7	1	6	0.61	3.79	3.06
	36	g510	45	341	8	8	0	8	3	5	7	0.70	8.07	5.37
	37	g523	45	362	2	2	1	1	1	1	3	0.53	1.53	1.26
	38	Leg044	44	102	0	0	0	0	0	0	1	0.00	0.00	0.00
	39	Leg100	45	47	0	0	0	0	0	0	1	0.00	0.00	0.00
	40	Leg133	45	240	1	1	0	1	1	0	2	0.17	0.69	0.95
	41	Leg223	41	145	0	0	0	0	0	0	1	0.00	0.00	0.00
	42	PvSHP1	45	96	1	1	1	0	0	1	2	0.04	0.46	2.38
		**Mean**	**44.4**	**266.6**	**3.6**	**3.6**	**0.8**	**2.8**	**2.2**	**1.4**	**3.8**	**0.37**	**3.12**	**2.86**
		**Overall**	**/**	**11,198**	**151**	**153**	**33**	**118**	**93**	**58**	**/**	**/**	**/**	**/**
Mesoamerican accessions of *P. vulgaris*	1	AN-Pv1	39	229	5	5	3	2	2	3	4	0.24	1.74	5.16
	2	AN-Pv2	39	351	1	1	0	1	1	0	2	0.36	1.04	0.67
	3	AN-Pv3	39	233	4	4	2	2	1	3	4	0.46	2.41	4.06
	4	AN-Pv4	39	258	0	0	0	0	0	0	1	0.00	0.00	0.00
	5	AN-Pv5	39	258	0	0	0	0	0	0	1	0.00	0.00	0.00
	6	AN-Pv8	38	386	7	7	1	6	5	2	6	0.61	4.24	4.32
	7	AN-Pv9	39	232	0	0	0	0	0	0	1	0.00	0.00	0.00
	8	AN-Pv10	39	213	4	4	0	4	2	2	3	0.62	8.26	4.44
	9	AN-Pv17	39	114	0	0	0	0	0	0	1	0.00	0.00	0.00
	10	AN-Pv18	39	408	10	10	9	1	4	6	3	0.15	1.38	5.80
	11	AN-Pv22	37	339	7	7	3	4	5	2	9	0.80	5.56	4.95
	12	AN-Pv26.1	39	204	6	7	2	4	4	3	8	0.63	8.23	6.96
	13	AN-Pv28	39	87	1	1	0	1	1	0	2	0.51	5.89	2.72
	14	AN-Pv29	39	78	1	1	0	1	0	1	2	0.27	3.43	3.03
	15	AN-Pv30	39	215	4	4	0	4	3	1	4	0.65	7.85	4.40
	16	AN-Pv32	39	137	0	0	0	0	0	0	1	0.00	0.00	0.00
	17	AN-Pv33	39	224	4	4	1	3	3	1	4	0.55	3.58	4.22
	18	AN-Pv35	39	45	0	0	0	0	0	0	1	0.00	0.00	0.00
	19	AN-Pv44	39	343	4	4	1	3	3	1	4	0.20	1.55	2.76

	20	AN-Pv46	39	380	4	4	1	3	0	4	6	0.69	3.51	2.49
	21	AN-Pv47	39	438	6	6	1	5	4	2	7	0.74	4.60	3.24
	22	AN-Pv51	37	318	8	8	2	6	8	0	5	0.61	5.96	6.03
	23	AN-Pv54	36	398	8	8	3	5	8	0	7	0.77	6.00	4.85
	24	AN-Pv55	39	390	0	0	0	0	0	0	1	0.00	0.00	0.00
	25	AN-Pv57	39	241	5	5	2	3	5	0	4	0.20	1.67	4.91
	26	AN-Pv63	39	600	8	8	3	5	4	4	7	0.76	4.27	3.15
	27	AN-Pv64	39	229	0	0	0	0	0	0	1	0.00	0.00	0.00
	28	AN-Pv66	39	265	4	4	3	1	0	3	3	0.15	0.96	3.57
	29	AN-Pv68	39	522	9	9	3	6	4	5	7	0.64	2.82	4.08
	30	AN-Pv69	39	273	1	1	0	1	1	0	2	0.23	0.84	0.87
	31	gssE18	39	70	2	2	0	2	1	1	2	0.39	11.18	6.76
	32	gssE20	39	80	0	0	0	0	0	0	1	0.00	0.00	0.00
	33	AN-PvCO	32	538	8	8	1	7	4	3	5	0.65	3.99	3.69
	34	AN-TGA	36	172	1	1	1	0	1	1	2	0.06	0.32	1.40
	35	AN-DNAJ	39	597	7	7	2	5	6	1	5	0.53	3.38	2.77
	36	g510	39	341	8	8	0	8	3	5	6	0.65	7.03	5.55
	37	g523	39	362	2	2	1	1	1	1	3	0.53	1.51	1.31
	38	Leg044	38	102	0	0	0	0	0	0	1	0.00	0.00	0.00
	39	Leg100	39	47	0	0	0	0	0	0	1	0.00	0.00	0.00
	40	Leg133	39	240	1	1	0	1	1	0	2	0.10	0.42	0.99
	41	Leg223	36	145	0	0	0	0	0	0	1	0.00	0.00	0.00
	42	PvSHP1	39	96	1	1	1	0	0	1	2	0.05	0.53	2.46
		**Mean**	**38.5**	**266.6**	**3.4**	**3.4**	**1.1**	**2.3**	**2.0**	**1.3**	**3.4**	**0.33**	**2.72**	**2.66**
		**Overall**	**/**	**11,198**	**141**	**142**	**46**	**95**	**85**	**56**	**/**	**/**	**/**	**/**
Mesoamerican wild (MW) accessions of *P. vulgaris*	1	AN-Pv1	19	229	5	5	3	2	2	3	4	0.45	3.47	6.25
	2	AN-Pv2	19	351	1	1	0	1	1	0	2	0.46	1.30	0.82
	3	AN-Pv3	19	233	4	4	2	2	1	3	4	0.61	3.51	4.91
	4	AN-Pv4	19	258	0	0	0	0	0	0	1	0.00	0.00	0.00
	5	AN-Pv5	19	258	0	0	0	0	0	0	1	0.00	0.00	0.00
	6	AN-Pv8	19	386	6	6	1	5	5	1	5	0.76	6.18	4.45
	7	AN-Pv9	19	232	0	0	0	0	0	0	1	0.00	0.00	0.00
	8	AN-Pv10	19	213	4	4	0	4	2	2	3	0.61	7.85	5.37
	9	AN-Pv17	19	114	0	0	0	0	0	0	1	0.00	0.00	0.00

10	AN-Pv18	19	408	9	9	9	0	4	5	2	0.11	2.32	6.31
11	AN-Pv22	18	339	7	7	3	4	5	2	7	0.69	4.57	6.00
12	AN-Pv26.1	19	204	6	7	2	4	4	3	8	0.67	8.40	8.42
13	AN-Pv28	19	87	1	1	0	1	1	0	2	0.52	5.92	3.29
14	AN-Pv29	19	78	1	1	0	1	0	1	2	0.41	5.25	3.67
15	AN-Pv30	19	215	4	4	0	4	3	1	4	0.68	7.83	5.32
16	AN-Pv32	19	137	0	0	0	0	0	0	1	0.00	0.00	0.00
17	AN-Pv33	19	224	4	4	1	3	3	1	4	0.63	5.59	5.11
18	AN-Pv35	19	45	0	0	0	0	0	0	1	0.00	0.00	0.00
19	AN-Pv44	19	343	3	3	0	3	2	1	3	0.29	1.98	2.50
20	AN-Pv46	19	380	4	4	1	3	0	4	6	0.80	3.57	3.01
21	AN-Pv47	19	438	6	6	1	5	4	2	6	0.83	4.51	3.92
22	AN-Pv51	17	318	8	8	5	3	8	0	5	0.73	5.13	7.44
23	AN-Pv54	16	398	6	6	1	5	6	0	4	0.73	4.84	4.54
24	AN-Pv55	19	390	0	0	0	0	0	0	1	0.00	0.00	0.00
25	AN-Pv57	19	241	5	5	5	0	5	0	4	0.30	2.18	5.94
26	AN-Pv63	19	600	8	8	3	5	4	4	7	0.78	4.37	3.81
27	AN-Pv64	19	229	0	0	0	0	0	0	1	0.00	0.00	0.00
28	AN-Pv66	19	265	4	4	3	1	0	3	3	0.29	1.94	4.32
29	AN-Pv68	19	522	9	9	4	5	4	5	6	0.71	3.52	4.93
30	AN-Pv69	19	273	1	1	0	1	1	0	2	0.41	1.50	1.05
31	gssE18	19	70	2	2	0	2	1	1	2	0.52	14.70	8.17
32	gssE20	19	80	0	0	0	0	0	0	1	0.00	0.00	0.00
33	AN-PvCO	15	538	7	7	3	4	4	2	4	0.66	4.57	4.00
34	AN-TGA	19	172	1	1	1	0	1	0	2	0.11	0.61	1.66
35	AN-DNAJ	19	597	6	6	1	5	6	0	4	0.71	4.41	2.88
36	g510	19	341	8	8	2	6	3	5	6	0.80	8.92	6.71
37	g523	19	362	1	1	0	1	1	0	2	0.46	7.27	0.79
38	Leg044	18	102	0	0	0	0	0	0	1	0.00	0.00	0.00
39	Leg100	19	47	0	0	0	0	0	0	1	0.00	0.00	0.00
40	Leg133	19	240	1	1	0	1	1	0	2	0.20	0.83	1.19
41	Leg223	17	145	0	0	0	0	0	0	1	0.00	0.00	0.00
42	PvSHP1	19	96	1	1	1	0	0	1	2	0.11	1.10	2.98
	**Mean**	**18.7**	**266.6**	**3.2**	**3.2**	**1.2**	**1.9**	**2.0**	**1.2**	**3.1**	**0.38**	**3.29**	**3.09**

		**Overall**	**/**	**11,198**	**133**	**134**	**52**	**81**	**82**	**50**	**/**	**/**	**/**	**/**
Mesoamerican	1	AN-Pv1	20	229	0	0	0	0	0	0	1	0.00	0.00	0.00
domesticated (MD)	2	AN-Pv2	20	351	1	1	0	1	1	0	2	0.27	0.76	0.80
accessions of *P. vulgaris*	3	AN-Pv3	20	233	1	1	0	1	0	1	2	0.27	1.15	1.21
	4	AN-Pv4	20	258	0	0	0	0	0	0	1	0.00	0.00	0.00
	5	AN-Pv5	20	258	0	0	0	0	0	0	1	0.00	0.00	0.00
	6	AN-Pv8	19	386	3	3	1	2	1	2	3	0.29	1.30	2.22
	7	AN-Pv9	20	232	0	0	0	0	0	0	1	0.00	0.00	0.00
	8	AN-Pv10	20	213	4	4	0	4	2	2	3	0.62	8.60	5.29
	9	AN-Pv17	20	114	0	0	0	0	0	0	1	0.00	0.00	0.00
	10	AN-Pv18	20	408	1	1	0	1	0	1	2	0.19	0.46	0.69
	11	AN-Pv22	19	339	3	3	0	3	2	1	4	0.58	3.42	2.53
	12	AN-Pv26.1	20	204	3	3	3	0	1	2	2	0.10	1.47	4.15
	13	AN-Pv28	20	87	1	1	0	1	1	0	2	0.51	5.81	3.24
	14	AN-Pv29	20	78	1	1	1	0	0	1	2	0.10	1.28	3.61
	15	AN-Pv30	20	215	3	3	0	3	2	1	2	0.44	6.17	3.93
	16	AN-Pv32	20	137	0	0	0	0	0	0	1	0.00	0.00	0.00
	17	AN-Pv33	20	224	0	0	0	0	0	0	1	0.00	0.00	0.00
	18	AN-Pv35	20	45	0	0	0	0	0	0	1	0.00	0.00	0.00
	19	AN-Pv44	20	343	4	4	4	0	3	1	2	0.10	1.17	3.29
	20	AN-Pv46	20	380	3	3	1	2	0	3	3	0.47	2.47	2.23
	21	AN-Pv47	20	438	4	4	0	4	3	1	4	0.55	3.77	2.57
	22	AN-Pv51	20	318	5	5	0	5	5	0	2	0.40	6.21	4.43
	23	AN-Pv54	20	398	7	7	2	5	7	0	6	0.76	6.76	4.96
	24	AN-Pv55	20	390	0	0	0	0	0	0	1	0.00	0.00	0.00
	25	AN-Pv57	20	241	3	3	3	0	3	0	2	0.10	1.24	3.51
	26	AN-Pv63	20	600	5	5	0	5	2	3	2	0.40	3.29	2.35
	27	AN-Pv64	20	229	0	0	0	0	0	0	1	0.00	0.00	0.00
	28	AN-Pv66	20	265	0	0	0	0	0	0	1	0.00	0.00	0.00
	29	AN-Pv68	20	522	4	4	1	3	1	3	4	0.60	2.22	2.16
	30	AN-Pv69	20	273	0	0	0	0	0	0	1	0.00	0.00	0.00
	31	gssE18	20	70	2	2	0	2	1	1	2	0.19	5.41	8.05
	32	gssE20	20	80	0	0	0	0	0	0	1	0.00	0.00	0.00
	33	AN-PvCO	17	538	7	7	2	5	4	2	4	0.63	3.31	3.85

34	AN-TGA	17	172	0	0	0	0	0	0	1	0.00	0.00	0.00
35	AN-DNAJ	20	597	4	4	4	0	3	1	3	0.20	0.67	1.89
36	g510	20	341	7	7	5	2	2	5	3	0.28	2.81	5.79
37	g523	20	362	2	2	1	1	1	1	3	0.35	1.02	1.56
38	Leg044	20	102	0	0	0	0	0	0	1	0.00	0.00	0.00
39	Leg100	20	47	0	0	0	0	0	0	1	0.00	0.00	0.00
40	Leg133	20	240	0	0	0	0	0	0	1	0.00	0.00	0.00
41	Leg223	19	145	0	0	0	0	0	0	1	0.00	0.00	0.00
42	PvSHP1	20	96	0	0	0	0	0	0	1	0.00	0.00	0.00
	**Mean**	**19.8**	**266.6**	**1.9**	**1.9**	**0.7**	**1.2**	**1.1**	**0.8**	**2.0**	**0.20**	**1.69**	**1.77**
	**Overall**	**/**	**11,198**	**78**	**78**	**28**	**50**	**45**	**32**	**/**	**/**	**/**	**/**

N, sample size; Range bp, sequence length (base pairs); V, variable sites; η, total number of mutations; S, singleton variable sites; Pi, parsimony informative variable sites; Syn, total number of synonymous changes; Nonsyn, total number of replacement changes; H, number of haplotypes; Hd, haplotype diversity; $\pi \times 10^{-3}$ and $\Theta \times 10^{-3}$, two measure of nucleotide diversity from Tajima (1983) and Watterson (1975), respectively.
