# Supplementary material for: High Level of Nonsynonymous Changes in Common Bean Suggests That Selection under Domestication Increased Functional Diversity at Target Traits
Source: Front Plant Sci. 2017 Jan 6;7:2005. doi: 10.3389/fpls.2016.02005 (PMC5216878; doi:10.3389/fpls.2016.02005)
Supplement: Supplementary file 6 [file Table6.PDF]

Table S6. Genetic diversity estimates computed considering the non coding regions (introns, 3’ and 5’-UTRs) of the 37 loci characterized and including introns and/ or UTR regions for the **a)** *P. vulgaris* sample, **b)** Mesoamerican accessions of *P. vulgaris*, **c)** Mesoamerican wild (MW) and **d)** domesticated (MD) accessions.

			**Locus**	**N**	**Range bp**	**V**	**η**	**S**	**Pi**	**H**	**Hd**	**π x 10^{-3}**	**Ө x 10^{-3}**
P. vulgaris sample	*Introns*	1	AN-Pv1	45	11 - 13	1	1	0	1	2	0.27	24.43	20.79
		2	AN-Pv3	45	140 - 146	3	3	3	0	2	0.04	0.95	4.90
		3	AN-Pv4	45	163 - 194	5	5	2	3	4	0.45	3.83	7.10
		4	AN-Pv5	45	228	3	3	1	2	3	0.28	2.27	3.01
		5	AN-Pv9	45	179 - 183	0	0	0	0	1	0.00	0.00	0.00
		6	AN-Pv10	45	130 - 131	4	4	1	3	5	0.38	3.81	7.04
		7	AN-Pv17	45	220 - 222	1	1	0	1	2	0.13	0.58	1.04
		8	AN-Pv22	43	105	4	4	1	3	5	0.69	10.04	8.80
		9	AN-Pv26.1	45	233-237	4	4	1	3	5	0.58	4.94	3.93
		10	AN-Pv28	45	236 - 244	6	6	1	5	5	0.67	10.09	5.84
		11	AN-Pv29	45	167	5	5	0	5	5	0.42	4.45	6.85
		12	AN-Pv32	45	145	5	5	1	4	3	0.21	4.88	7.89
		13	AN-Pv35	45	54	2	3	1	1	3	0.13	3.22	8.47
		14	AN-Pv41	45	133 - 150	3	3	1	2	4	0.28	2.23	5.16
		15	AN-Pv44	45	108	0	0	0	0	1	0.00	0.00	0.00
		16	AN-Pv47	45	99 - 100	5	5	0	5	3	0.53	20.30	11.55
		17	AN-Pv51	43	382	12	12	2	10	7	0.69	5.82	7.26
		18	AN-Pv55	45	211	0	0	0	0	1	0.00	0.00	0.00
		19	AN-Pv57	45	216	7	8	0	7	4	0.54	12.14	7.41
		20	AN-Pv64	45	308 - 360	9	9	0	9	6	0.43	4.76	6.68
		21	AN-Pv68	44	195 - 206	14	14	6	8	7	0.50	12.74	16.50
		22	AN-Pv69	45	128 - 129	0	0	0	0	1	0.00	0.00	0.00
		23	gssE18	45	201	6	6	2	4	6	0.69	6.87	6.83
		24	gssE20	45	197 - 200	1	1	0	1	2	0.09	0.44	1.16
		25	AN-PvCO	38	113 - 117	9	9	0	9	6	0.65	20.11	19.30
		26	AN-TGA	42	408 - 435	14	14	2	12	8	0.80	7.13	7.97
		27	g510	45	180 - 186	2	2	1	1	3	0.39	2.21	2.54
		28	Leg044	44	764 - 765	22	22	1	21	8	0.79	6.73	6.64
		29	Leg100	45	552 - 666	42	44	2	40	9	0.83	26.26	18.58
		30	Leg133	45	344 - 350	11	12	0	11	5	0.67	8.96	7.31

		31	Leg223	41	326 - 360	7	7	1	6	8	0.71	4.21	5.10
		32	PvSHP1	45	779 - 793	39	42	8	31	17	0.79	10.84	11.88
			**Mean**	**44.4**	**239.2-250.1**	**7.7**	**7.9**	**1.2**	**6.5**	**4.7**	**0.43**	**7.04**	**7.11**
			**Overall**	**/**	**7,655-8,004**	**246**	**254**	**38**	**208**	**/**	**/**	**/**	**/**
	5'UTR	1	AN-Pv16	43	296 - 297	8	8	1	7	9	0.60	5.85	6.25
		2	AN-Pv69	45	268	2	2	0	2	3	0.53	2.19	1.71
			**Mean**	**44.0**	**282.0 - 282.5**	**5.0**	**5.0**	**0.5**	**4.5**	**6.0**	**0.56**	**4.02**	**3.98**
			**Overall**	**/**	**564 - 565**	**10**	**10**	**1**	**9**	**/**	**/**	**/**	**/**
	3'UTR	1	AN-Pv2	45	67	4	4	0	4	4	0.52	21.56	13.65
		2	AN-Pv26.1	45	241 - 244	5	5	0	5	4	0.70	8.31	4.74
		3	AN-Pv35	45	110	1	1	0	1	2	0.09	0.79	2.08
		4	AN-Pv42	45	185 - 186	5	5	1	4	4	0.47	0.87	6.21
		5	AN-Pv46	45	118 - 131	0	0	0	0	1	0.00	0.00	0.00
		6	g510	45	35 - 38	0	0	0	0	1	0.00	0.00	0.00
		7	g523	45	120	1	1	0	1	2	0.51	4.24	1.91
			**Mean**	**45.0**	**125.1 - 128.0**	**2.3**	**2.3**	**0.1**	**2.1**	**2.6**	**0.33**	**5.11**	**4.08**
			**Overall**	**/**	**876 - 896**	**16**	**16**	**1**	**15**	**/**	**/**	**/**	**/**
Mesoamerican accessions of *P. vulgaris*	*Introns*	1	AN-Pv1	39	11-13	1	1	0	1	2	0.23	20.86	21.50
		2	AN-Pv3	39	146	3	3	3	0	2	0.05	1.05	4.86
		3	AN-Pv4	39	163 - 194	3	3	2	1	3	0.43	3.07	4.41
		4	AN-Pv5	39	228	3	3	1	2	3	0.15	1.10	3.11
		5	AN-Pv9	39	179 -183	0	0	0	0	1	0.00	0.00	0.00
		6	AN-Pv10	39	130 - 131	4	4	1	3	5	0.36	3.74	7.28
		7	AN-Pv17	39	222	1	1	0	1	2	0.15	0.66	1.07
		8	AN-Pv22	37	105	4	4	1	3	5	0.70	10.70	9.13
		9	AN-Pv26.1	39	233 - 237	4	4	1	3	5	0.58	4.87	4.06
		10	AN-Pv28	39	236 - 244	5	5	0	5	4	0.60	9.41	5.03
		11	AN-Pv29	39	167	4	4	0	4	4	0.40	4.48	5.67
		12	AN-Pv32	39	145	0	0	0	0	1	0.00	0.00	0.00
		13	AN-Pv35	39	54	1	1	0	1	2	0.10	1.85	4.38
		14	AN-Pv41	39	133 - 150	3	3	1	2	4	0.24	1.89	5.34
		15	AN-Pv44	39	108	0	0	0	0	1	0.00	0.00	0.00
		16	AN-Pv47	39	99 - 100	5	5	0	5	3	0.56	21.40	11.95

		17	AN-Pv51	37	382	11	11	1	10	6	0.62	5.88	6.90
		18	AN-Pv55	39	211	0	0	0	0	1	0.00	0.00	0.00
		19	AN-Pv57	39	216	7	8	0	7	4	0.48	11.76	7.67
		20	AN-Pv64	39	308	8	8	4	4	5	0.40	3.66	6.14
		21	AN-Pv68	39	205 - 206	9	9	5	4	6	0.37	6.37	10.38
		22	AN-Pv69	39	128 - 129	0	0	0	0	1	0.00	0.00	0.00
		23	gssE18	39	201	6	6	2	4	6	0.66	6.41	7.06
		24	gssE20	39	197 - 200	0	0	0	0	1	0.00	0.00	0.00
		25	AN-PvCO	32	113 - 117	9	9	0	9	6	0.56	20.54	20.13
		26	AN-TGA	36	408 - 409	13	13	2	11	7	0.77	7.22	7.68
		27	g510	39	180 - 186	2	2	1	1	3	0.31	1.77	2.63
		28	Leg044	38	764 - 765	22	22	12	10	7	0.73	5.77	6.85
		29	Leg100	39	552 -666	39	41	2	37	8	0.81	23.80	17.81
		30	Leg133	39	344 - 350	11	12	0	11	5	0.60	8.36	7.56
		31	Leg223	36	326 - 360	6	6	1	5	7	0.64	3.39	4.51
		32	PvSHP1	39	779 - 790	38	40	8	30	15	0.73	10.98	11.69
			**Mean**	**38.4**	**239.8 - 247.6**	**6.9**	**7.1**	**1.5**	**5.4**	**4.2**	**0.38**	**6.28**	**6.40**
			**Overall**	**/**	**7,673 – 7,923**	**222**	**228**	**48**	**174**	**/**	**/**	**/**	**/**
	5'UTR	1	AN-Pv16	37	296 - 297	7	7	1	6	8	0.47	4.36	5.66
		2	AN-Pv69	39	268	1	1	0	1	2	0.47	1.76	0.88
			**Mean**	**38.0**	**282.0 - 282.5**	**4.0**	**4.0**	**0.5**	**3.5**	**5.0**	**0.47**	**3.06**	**3.27**
			**Overall**	**/**	**564 - 565**	**8**	**8**	**1**	**7**	**/**	**/**	**/**	**/**
	3'UTR	1	AN-Pv2	39	67	4	4	0	4	4	0.40	16.52	14.12
		2	AN-Pv26.1	39	241 - 244	5	5	0	5	4	0.68	8.38	4.91
		3	AN-Pv35	39	110	1	1	0	1	2	0.10	0.91	2.15
		4	AN-Pv42	39	185 - 186	5	5	1	4	4	0.52	7.60	6.43
		5	AN-Pv46	39	118 - 131	0	0	0	0	1	0.00	0.00	0.00
		6	g510	39	38	0	0	0	0	1	0.00	0.00	0.00
		7	g523	39	120	1	1	0	1	2	0.50	4.14	1.97
			**Mean**	**39.0**	**125.6 - 128.0**	**2.3**	**2.3**	**0.1**	**2.1**	**2.6**	**0.31**	**5.36**	**4.23**
			**Overall**	**/**	**879 -896**	**16**	**16**	**1**	**15**	**/**	**/**	**/**	**/**
Mesoamerican wild (MW)	*Introns*	1	AN-Pv1	19	11-13	1	1	0	1	2	0.41	37.21	26.01
		2	AN-Pv3	19	146	3	3	3	0	2	0.11	2.16	5.88

accessions of *P. vulgaris*	3	AN-Pv4	19	163 - 194	3	3	2	1	3	0.57	4.50	5.33
	4	AN-Pv5	19	228	3	3	1	2	3	0.29	2.21	3.76
	5	AN-Pv9	19	179 -183	0	0	0	0	1	0.00	0.00	0.00
	6	AN-Pv10	19	130 - 131	4	4	2	2	5	0.46	5.31	8.80
	7	AN-Pv17	19	222	1	1	0	1	2	0.28	1.26	1.29
	8	AN-Pv22	18	105	4	4	1	3	5	0.67	10.83	11.08
	9	AN-Pv26.1	19	233 - 237	4	4	1	3	5	0.46	4.57	4.91
	10	AN-Pv28	19	236 - 244	5	5	0	5	4	0.70	10.20	6.09
	11	AN-Pv29	19	167	3	3	0	3	3	0.37	5.39	5.14
	12	AN-Pv32	19	145	0	0	0	0	1	0.00	0.00	0.00
	13	AN-Pv35	19	54	1	1	0	1	2	0.20	3.68	5.30
	14	AN-Pv41	19	133 - 150	3	3	2	1	4	0.38	3.08	6.45
	15	AN-Pv44	19	108	0	0	0	0	1	0.00	0.00	0.00
	16	AN-Pv47	19	99 - 100	5	5	0	5	3	0.50	16.19	14.45
	17	AN-Pv51	17	382	11	11	7	4	6	0.76	4.70	8.52
	18	AN-Pv55	19	211	0	0	0	0	1	0.00	0.00	0.00
	19	AN-Pv57	19	216	7	8	1	6	4	0.45	10.56	9.27
	20	AN-Pv64	19	308	8	8	4	4	5	0.70	6.38	7.43
	21	AN-Pv68	19	205 - 206	8	8	5	3	4	0.46	7.65	11.17
	22	AN-Pv69	19	128 - 129	0	0	0	0	1	0.00	0.00	0.00
	23	gssE18	19	201	5	5	1	4	5	0.77	9.08	7.12
	24	gssE20	19	197 - 200	0	0	0	0	1	0.00	0.00	0.00
	25	AN-PvCO	15	113 - 117	9	9	1	8	6	0.71	28.83	24.94
	26	AN-TGA	19	408 - 409	13	13	3	10	6	0.78	11.15	9.12
	27	g510	19	180 - 186	2	2	1	1	3	0.37	2.14	3.18
	28	Leg044	18	764 - 765	22	22	13	9	6	0.81	7.01	8.37
	29	Leg100	19	552 -666	38	40	2	36	7	0.88	30.03	20.99
	30	Leg133	19	344 - 350	11	12	0	11	5	0.81	12.72	9.15
	31	Leg223	17	326 - 360	6	6	1	5	7	0.87	5.68	5.53
	32	PvSHP1	19	779 - 790	38	40	9	29	14	0.97	1.68	14.14
		**Mean**	**18.7**	**239.8 - 247.6**	**6.8**	**7.0**	**1.9**	**4.9**	**4.0**	**0.46**	**7.63**	**7.61**
		**Overall**	**/**	**7,673 – 7,923**	**218**	**224**	**60**	**158**	**/**	**/**	**/**	**/**
5'UTR	1	AN-Pv16	17	296 - 297	7	7	1	6	8	0.82	7.45	7.00
	2	AN-Pv69	19	268	1	1	0	1	2	0.41	1.53	1.07

			**Mean**	**18.0**	**282.0 - 282.5**	**4.0**	**4.0**	**0.5**	**3.5**	**5.0**	**0.61**	**4.49**	**4.04**
			**Overall**	**/**	**564 - 565**	**8**	**8**	**1**	**7**	**/**	**/**	**/**	**/**
	3'UTR	1	AN-Pv2	19	67	4	4	1	3	3	0.43	19.03	17.08
		2	AN-Pv26.1	19	241 - 244	5	5	3	2	4	0.70	4.51	5.94
		3	AN-Pv35	19	110	1	1	0	1	2	0.20	1.81	2.60
		4	AN-Pv42	19	185 - 186	5	5	1	4	4	0.66	9.66	7.77
		5	AN-Pv46	19	118 - 131	0	0	0	0	1	0.00	0.00	0.00
		6	g510	19	38	0	0	0	0	1	0.00	0.00	0.00
		7	g523	19	120	1	1	0	1	2	0.46	3.80	2.38
			**Mean**	**19.0**	**125.6 - 128.0**	**2.3**	**2.3**	**0.7**	**1.6**	**2.4**	**0.35**	**5.54**	**5.11**
			**Overall**	**/**	**879 -896**	**16**	**16**	**5**	**11**	**/**	**/**	**/**	**/**
Mesoamerican domesticated (MD) accessions of *P. vulgaris*	*Introns*	1	AN-Pv1	20	11-13	0	0	0	0	1	0.00	0.00	0.00
		2	AN-Pv3	20	146	0	0	0	0	1	0.00	0.00	0.00
		3	AN-Pv4	20	163 - 194	1	1	0	1	2	0.19	1.18	1.75
		4	AN-Pv5	20	228	0	0	0	0	1	0.00	0.00	0.00
		5	AN-Pv9	20	179 -183	0	0	0	0	1	0.00	0.00	0.00
		6	AN-Pv10	20	130 - 131	1	1	0	1	2	0.27	2.06	2.17
		7	AN-Pv17	20	222	0	0	0	0	1	0.00	0.00	0.00
		8	AN-Pv22	19	105	1	1	0	1	2	0.35	3.34	2.72
		9	AN-Pv26.1	20	233 - 237	2	2	2	0	2	0.10	0.86	2.42
		10	AN-Pv28	20	236 - 244	4	4	0	4	2	0.51	8.60	4.80
		11	AN-Pv29	20	167	3	3	2	1	3	0.42	3.21	5.06
		12	AN-Pv32	20	145	0	0	0	0	1	0.00	0.00	0.00
		13	AN-Pv35	20	54	0	0	0	0	1	0.00	0.00	0.00
		14	AN-Pv41	20	133 - 150	1	1	1	0	2	0.10	0.75	2.12
		15	AN-Pv44	20	108	0	0	0	0	1	0.00	0.00	0.00
		16	AN-Pv47	20	99 - 100	4	4	0	4	2	0.48	19.35	11.39
		17	AN-Pv51	20	382	7	7	1	6	3	0.47	6.46	5.17
		18	AN-Pv55	20	211	0	0	0	0	1	0.00	0.00	0.00
		19	AN-Pv57	20	216	7	8	1	6	3	0.51	13.23	9.13
		20	AN-Pv64	20	308	0	0	0	0	1	0.00	0.00	0.00
		21	AN-Pv68	20	205 - 206	5	5	1	4	3	0.28	5.34	6.87
		22	AN-Pv69	20	128 - 129	0	0	0	0	1	0.00	0.00	0.00
		23	gssE18	20	201	3	3	1	2	3	0.20	2.38	4.21

	24	gssE20	20	197 - 200	0	0	0	0	1	0.00	0.00	0.00
	25	AN-PvCO	17	113 - 117	5	5	1	4	3	0.40	12.19	13.32
	26	AN-TGA	17	408 - 409	3	3	3	0	3	0.23	0.87	2.17
	27	g510	20	180 - 186	1	1	0	1	2	0.27	1.49	1.57
	28	Leg044	20	764 - 765	10	10	1	9	4	0.64	4.78	3.69
	29	Leg100	20	552 -666	23	23	0	23	3	0.63	15.89	12.52
	30	Leg133	20	344 - 350	1	1	0	1	2	0.19	0.55	0.82
	31	Leg223	19	326 - 360	0	0	0	0	1	0.00	0.00	0.00
	32	PvSHP1	20	779 - 790	21	21	21	0	3	0.20	2.73	7.70
		**Mean**	**19.8**	**239.8 - 247.6**	**3.2**	**3.3**	**1.1**	**2.1**	**1.9**	**0.20**	**3.29**	**3.11**
		**Overall**	**/**	**7,673 – 7,923**	**103**	**104**	**35**	**68**	**/**	**/**	**/**	**/**
5'UTR	1	AN-Pv16	20	296 - 297	0	0	0	0	1	0.00	0.00	0.00
	2	AN-Pv69	20	268	0	0	0	0	1	0.00	0.00	0.00
		**Mean**	**20.0**	**282.0 - 282.5**	**0.0**	**0.0**	**0.0**	**0.0**	**1.0**	**0.0**	**0.0**	**0.0**
		**Overall**	**/**	**564 - 565**	**0**	**0**	**0**	**0**	**/**	**/**	**/**	**/**
3'UTR	1	AN-Pv2	20	67	3	3	0	3	3	0.35	14.06	12.62
	2	AN-Pv26.1	20	241 - 244	4	4	1	3	3	0.20	2.77	4.68
	3	AN-Pv35	20	110	0	0	0	0	1	0.00	0.00	0.00
	4	AN-Pv42	20	185 - 186	3	3	0	3	2	0.34	5.49	4.60
	5	AN-Pv46	20	118 - 131	0	0	0	0	1	0.00	0.00	0.00
	6	g510	20	38	0	0	0	0	1	0.00	0.00	0.00
	7	g523	20	120	1	1	0	1	2	0.27	2.24	2.35
		**Mean**	**20.0**	**125.6 - 128.0**	**1.6**	**1.6**	**0.1**	**1.4**	**1.9**	**0.16**	**3.51**	**3.46**
		**Overall**	**/**	**879 -896**	**11**	**11**	**1**	**10**	**/**	**/**	**/**	**/**

N, sample size; bp. sequence length (base pairs); V, variable sites; η, total number of mutations; S, singleton variable sites; Pi. parsimony informative variable sites; H, number of haplotypes; Hd, haplotype diversity; $\pi \times 10^{-3}$ and $\Theta \times 10^{-3}$, two measure of nucleotide diversity from Tajima (1983) and Watterson (1975), respectively.
