# Supplementary material for: High Level of Nonsynonymous Changes in Common Bean Suggests That Selection under Domestication Increased Functional Diversity at Target Traits
Source: Front Plant Sci. 2017 Jan 6;7:2005. doi: 10.3389/fpls.2016.02005 (PMC5216878; doi:10.3389/fpls.2016.02005)
Supplement: Supplementary file 7 [file Table7.PDF]

Table S7. Genetic diversity estimates computed considering the non coding regions of the 37 loci characterized and including introns and/ or UTR regions for the **a)** *P. vulgaris* sample, **b)** Mesoamerican accessions of *P. vulgaris*, **c)** Mesoamerican wild (MW) and **d)** domesticated (MD) accessions.

		**Locus**	**N**	**Range bp**	**V**	**η**	**S**	**Pi**	**H**	**Hd**	**π x 10^{-3}**	**Θ x 10^{-3}**
P. vulgaris sample	**1**	**AN-Pv1**	45	11 - 13	1	1	0	1	2	0.27	24.43	20.79
	**2**	**AN-*Pv* 2**	45	67	4	4	0	4	4	0.52	21.56	13.65
	**3**	**AN-Pv3**	45	140 - 146	3	3	3	0	2	0.04	0.95	4.90
	**4**	**AN-Pv4**	45	163 - 194	5	5	2	3	4	0.45	3.83	7.10
	**5**	**AN-Pv5**	45	228	3	3	1	2	3	0.28	2.27	3.01
	**6**	**AN-Pv9**	45	179 - 183	0	0	0	0	1	0.00	0.00	0.00
	**7**	**AN-Pv10**	45	130 - 131	4	4	1	3	5	0.38	3.81	7.04
	**8**	**AN-*Pv* 16**	43	296 - 297	8	8	1	7	9	0.60	5.85	6.25
	**9**	**AN-Pv17**	45	220 - 222	1	1	0	1	2	0.13	0.58	1.04
	**10**	**AN-Pv22**	43	105	4	4	1	3	5	0.69	10.04	8.80
	**11**	**AN-Pv26.1**	45	474 - 479	9	9	1	8	8	0.74	6.65	4.34
	**12**	**AN-Pv28**	45	236 - 244	6	6	1	5	5	0.67	10.09	5.84
	**13**	**AN-Pv29**	45	167	5	5	0	5	5	0.42	4.45	6.85
	**14**	**AN-Pv32**	45	145	5	5	1	4	3	0.21	4.88	7.89
	**15**	**AN-Pv35**	45	164	3	4	1	2	3	0.13	1.59	4.18
	**16**	**AN-Pv41**	45	133 - 150	3	3	1	2	4	0.28	2.23	5.16
	**17**	**AN-*Pv* 42**	45	185 - 186	5	5	1	4	4	0.47	0.87	6.21
	**18**	**AN-Pv44**	45	108	0	0	0	0	1	0.00	0.00	0.00
	**19**	**AN-*Pv* 46**	45	118 - 131	0	0	0	0	1	0.00	0.00	0.00
	**20**	**AN-Pv47**	45	99 - 100	5	5	0	5	3	0.53	20.30	11.55
	**21**	**AN-Pv51**	43	382	12	12	2	10	7	0.69	5.82	7.26
	**22**	**AN-Pv55**	45	211	0	0	0	0	1	0.00	0.00	0.00
	**23**	**AN-Pv57**	45	216	7	8	0	7	4	0.54	12.14	7.41
	**24**	**AN-Pv64**	45	308 - 360	9	9	0	9	6	0.43	4.76	6.68
	**25**	**AN-Pv68**	44	195 - 206	14	14	6	8	7	0.50	12.74	16.50
	**26**	**AN-Pv69**	45	396 - 397	2	2	0	2	3	0.53	1.48	1.16
	**27**	**gssE18**	45	201	6	6	2	4	6	0.69	6.87	6.83
	**28**	**gssE20**	45	197 - 200	1	1	0	1	2	0.09	0.44	1.16
	**29**	**AN-*PvCO***	38	113 - 117	9	9	0	9	6	0.65	20.11	19.30

	**30**	**AN-*TGA***	42	408 - 435	14	14	2	12	8	0.80	7.13	7.97
	**31**	**g510**	45	216 - 224	2	2	1	1	3	0.39	1.85	2.13
	**32**	**g523**	45	120	1	1	0	1	2	0.51	4.24	1.91
	**33**	**Leg044**	44	764 - 765	22	22	1	21	8	0.79	6.73	6.64
	**34**	**Leg100**	45	552 - 666	42	44	2	40	9	0.83	26.26	18.58
	**35**	**Leg133**	45	344 - 350	11	12	0	11	5	0.67	8.96	7.31
	**36**	**Leg223**	41	326 - 360	7	7	1	6	8	0.71	4.21	5.10
	**37**	**PvSHP1**	45	779 - 793	39	42	8	31	17	0.79	10.84	11.88
		**Mean**	**44.4**	**245.8 - 255.8**	**7.4**	**7.6**	**1.1**	**6.3**	**4.8**	**0.44**	**7.00**	**6.82**
		**Overall**	**/**	**9,096 - 9,463**	**272**	**280**	**40**	**232**	**/**	**/**	**/**	**/**
Mesoamerican accessions of *P. vulgaris*	**1**	**AN-Pv1**	39	11 - 13	1	1	0	1	2	0.23	20.86	21.50
	**2**	**AN-Pv2**	39	67	4	4	0	4	4	0.40	16.52	14.12
	**3**	**AN-Pv3**	39	146	3	3	3	0	2	0.05	1.05	4.86
	**4**	**AN-Pv4**	39	163 - 194	3	3	2	1	3	0.43	3.07	4.41
	**5**	**AN-Pv5**	39	228	3	3	1	2	3	0.15	1.10	3.11
	**6**	**AN-Pv9**	39	179 - 183	0	0	0	0	1	0.00	0.00	0.00
	**7**	**AN-Pv10**	39	130 - 131	4	4	1	3	5	0.36	3.74	7.28
	**8**	**AN-Pv16**	37	296 - 297	7	7	1	6	8	0.47	4.36	5.66
	**9**	**AN-Pv17**	39	222	1	1	0	1	2	0.15	0.66	1.07
	**10**	**AN-Pv22**	37	105	4	4	1	3	5	0.70	10.70	9.13
	**11**	**AN-Pv26.1**	39	474 - 479	9	9	1	8	8	0.72	6.65	4.49
	**12**	**AN-Pv28**	39	236 - 244	5	5	0	5	4	0.60	9.41	5.03
	**13**	**AN-Pv29**	39	167	4	4	0	4	4	0.40	4.48	5.67
	**14**	**AN-Pv32**	39	145	0	0	0	0	1	0.00	0.00	0.00
	**15**	**AN-Pv35**	39	164	2	2	0	2	2	0.10	1.22	2.88
	**16**	**AN-Pv41**	39	133 - 150	3	3	1	2	4	0.24	1.89	5.34
	**17**	**AN-Pv42**	39	185 - 186	5	5	1	4	4	0.52	7.60	6.43
	**18**	**AN-Pv44**	39	108	0	0	0	0	1	0.00	0.00	0.00
	**19**	**AN-Pv46**	39	118 - 131	0	0	0	0	1	0.00	0.00	0.00
	**20**	**AN-Pv47**	39	99 - 100	5	5	0	5	3	0.56	21.40	11.95
	**21**	**AN-Pv51**	37	382	11	11	1	10	6	0.62	5.88	6.90
	**22**	**AN-Pv55**	39	211	0	0	0	0	1	0.00	0.00	0.00
	**23**	**AN-Pv57**	39	216	7	8	0	7	4	0.48	11.76	7.67

	**24**	**AN-Pv64**	39	308	8	8	4	4	5	0.40	3.66	6.14
	**25**	**AN-Pv68**	39	205 - 206	9	9	5	4	6	0.37	6.37	10.38
	**26**	**AN-Pv69**	39	396 - 397	1	1	0	1	2	0.47	1.19	0.60
	**27**	**gssE18**	39	201	6	6	2	4	6	0.66	6.41	7.06
	**28**	**gssE20**	39	197 - 200	0	0	0	0	1	0.00	0.00	0.00
	**29**	**AN-*PvCO***	32	113 - 117	9	9	0	9	6	0.56	20.54	20.13
	**30**	**AN-*TGA***	36	408 - 409	13	13	2	11	7	0.77	7.22	7.68
	**31**	**g510**	39	218 - 224	2	2	1	1	3	0.31	1.46	2.17
	**32**	**g523**	39	120	1	1	0	1	2	0.50	4.14	1.97
	**33**	**Leg044**	38	764 - 765	22	22	12	10	7	0.73	5.77	6.85
	**34**	**Leg100**	39	552 - 666	39	41	2	37	8	0.81	23.80	17.81
	**35**	**Leg133**	39	344 - 350	11	12	0	11	5	0.60	8.36	7.56
	**36**	**Leg223**	36	326 - 360	6	6	1	5	7	0.64	3.39	4.51
	**37**	**PvSHP1**	39	779 - 790	38	40	8	30	15	0.73	10.98	11.69
		**Mean**	**38.5**	**246.4 - 253.6**	**6.6**	**6.8**	**1.4**	**5.3**	**4.3**	**0.40**	**6.37**	**6.27**
		**Overall**	**/**	**9,116 - 9,382**	**246**	**252**	**50**	**196**	**/**	**/**	**/**	**/**
Mesoamerican wild (MW) accessions of *P. vulgaris*	**1**	**AN-Pv1**	19	11 - 13	1	1	0	1	2	0.41	37.21	26.01
	**2**	**AN-Pv2**	19	67	4	4	1	3	3	0.43	19.03	17.08
	**3**	**AN-Pv3**	19	146	3	3	3	0	2	0.11	2.16	5.88
	**4**	**AN-Pv4**	19	163 - 194	3	3	2	1	3	0.57	4.50	5.33
	**5**	**AN-Pv5**	19	228	3	3	1	2	3	0.29	2.21	3.76
	**6**	**AN-Pv9**	19	179 - 183	0	0	0	0	1	0.00	0.00	0.00
	**7**	**AN-Pv10**	19	130 - 131	4	4	2	2	5	0.46	5.31	8.80
	**8**	**AN-Pv16**	17	296 - 297	7	7	1	6	8	0.82	7.45	7.00
	**9**	**AN-Pv17**	19	222	1	1	0	1	2	0.28	1.26	1.29
	**10**	**AN-Pv22**	18	105	4	4	1	3	5	0.67	10.83	11.08
	**11**	**AN-Pv26.1**	19	474 - 479	9	9	4	5	8	0.82	4.54	5.43
	**12**	**AN-Pv28**	19	236 - 244	5	5	0	5	4	0.70	10.20	6.09
	**13**	**AN-Pv29**	19	167	3	3	0	3	3	0.37	5.39	5.14
	**14**	**AN-Pv32**	19	145	0	0	0	0	1	0.00	0.00	0.00
	**15**	**AN-Pv35**	19	164	2	2	0	2	2	0.20	2.42	3.49
	**16**	**AN-Pv41**	19	133 - 150	3	3	2	1	4	0.38	3.08	6.45
	**17**	**AN-Pv42**	19	185 - 186	5	5	1	4	4	0.66	9.66	7.77

	**18**	**AN-Pv44**	19	108	0	0	0	0	1	0.00	0.00	0.00
	**19**	**AN-Pv46**	19	118 - 131	0	0	0	0	1	0.00	0.00	0.00
	**20**	**AN-Pv47**	19	99 - 100	5	5	0	5	3	0.50	16.19	14.45
	**21**	**AN-Pv51**	17	382	11	11	7	4	6	0.76	4.70	8.52
	**22**	**AN-Pv55**	19	211	0	0	0	0	1	0.00	0.00	0.00
	**23**	**AN-Pv57**	19	216	7	8	1	6	4	0.45	10.56	9.27
	**24**	**AN-Pv64**	19	308	8	8	4	4	5	0.70	6.38	7.43
	**25**	**AN-Pv68**	19	205 - 206	8	8	5	3	4	0.46	7.65	11.17
	**26**	**AN-Pv69**	19	396 - 397	1	1	0	1	2	0.41	1.03	0.72
	**27**	**gssE18**	19	201	5	5	1	4	5	0.77	9.08	7.12
	**28**	**gssE20**	19	197 - 200	0	0	0	0	1	0.00	0.00	0.00
	**29**	**AN-*PvCO***	15	113 - 117	9	9	1	8	6	0.71	28.83	24.94
	**30**	**AN-*TGA***	19	408 - 409	13	13	3	10	6	0.78	11.15	9.12
	**31**	**g510**	19	218 - 224	2	2	1	1	3	0.37	1.77	2.62
	**32**	**g523**	19	120	1	1	0	1	2	0.46	3.80	2.38
	**33**	**Leg044**	18	764 - 765	22	22	13	9	6	0.81	7.01	8.37
	**34**	**Leg100**	19	552 - 666	38	40	2	36	7	0.88	30.03	20.99
	**35**	**Leg133**	19	344 - 350	11	12	0	11	5	0.81	12.72	9.15
	**36**	**Leg223**	17	326 - 360	6	6	1	5	7	0.87	5.68	5.53
	**37**	**PvSHP1**	19	779 - 790	38	40	9	29	14	0.97	1.68	14.14
		**Mean**	**18.7**	**246.4 - 253.6**	**6.5**	**6.7**	**1.8**	**4.8**	**4.0**	**0.48**	**7.66**	**7.47**
		**Overall**	/	**9,116 - 9,382**	**242**	**248**	**66**	**176**	/	/	/	/
Mesoamerican domesticated (MD) accessions of *P. vulgaris*	**1**	**AN-Pv1**	20	11 - 13	0	0	0	0	1	0.00	0.00	0.00
	**2**	**AN-Pv2**	20	67	3	3	0	3	3	0.35	14.06	12.62
	**3**	**AN-Pv3**	20	146	0	0	0	0	1	0.00	0.00	0.00
	**4**	**AN-Pv4**	20	163 - 194	1	1	0	1	2	0.19	1.18	1.75
	**5**	**AN-Pv5**	20	228	0	0	0	0	1	0.00	0.00	0.00
	**6**	**AN-Pv9**	20	179 - 183	0	0	0	0	1	0.00	0.00	0.00
	**7**	**AN-Pv10**	20	130 - 131	1	1	0	1	2	0.27	2.06	2.17
	**8**	**AN-Pv16**	20	296 - 297	0	0	0	0	1	0.00	0.00	0.00
	**9**	**AN-Pv17**	20	222	0	0	0	0	1	0.00	0.00	0.00
	**10**	**AN-Pv22**	19	105	1	1	0	1	2	0.35	3.34	2.72
	**11**	**AN-Pv26.1**	20	474 - 479	6	6	3	3	3	0.20	1.83	3.57

**12**	**AN-Pv28**	20	236 - 244	4	4	0	4	2	0.51	8.60	4.80
**13**	**AN-Pv29**	20	167	3	3	2	1	3	0.42	3.21	5.06
**14**	**AN-Pv32**	20	145	0	0	0	0	1	0.00	0.00	0.00
**15**	**AN-Pv35**	20	164	0	0	0	0	1	0.00	0.00	0.00
**16**	**AN-Pv41**	20	133 - 150	1	1	1	0	2	0.10	0.75	2.12
**17**	**AN-Pv42**	20	185 - 186	3	3	0	3	2	0.34	5.49	4.60
**18**	**AN-Pv44**	20	108	0	0	0	0	1	0.00	0.00	0.00
**19**	**AN-Pv46**	20	118 - 131	0	0	0	0	1	0.00	0.00	0.00
**20**	**AN-Pv47**	20	99 - 100	4	4	0	4	2	0.48	19.35	11.39
**21**	**AN-Pv51**	20	382	7	7	1	6	3	0.47	6.46	5.17
**22**	**AN-Pv55**	20	211	0	0	0	0	1	0.00	0.00	0.00
**23**	**AN-Pv57**	20	216	7	8	1	6	3	0.51	13.23	9.13
**24**	**AN-Pv64**	20	308	0	0	0	0	1	0.00	0.00	0.00
**25**	**AN-Pv68**	20	205 - 206	5	5	1	4	3	0.28	5.34	6.87
**26**	**AN-Pv69**	20	396 - 397	0	0	0	0	1	0.00	0.00	0.00
**27**	**gssE18**	20	201	3	3	1	2	3	0.20	2.38	4.21
**28**	**gssE20**	20	197 - 200	0	0	0	0	1	0.00	0.00	0.00
**29**	**AN-*PvCO***	17	113 - 117	5	5	1	4	3	0.40	12.19	13.32
**30**	**AN-*TGA***	17	408 - 409	3	3	3	0	3	0.23	0.87	2.17
**31**	**g510**	20	218 - 224	1	1	0	1	2	0.27	1.23	1.29
**32**	**g523**	20	120	1	1	0	1	2	0.27	2.24	2.35
**33**	**Leg044**	20	764 - 765	10	10	1	9	4	0.64	4.78	3.69
**34**	**Leg100**	20	552 - 666	23	23	0	23	3	0.63	15.89	12.52
**35**	**Leg133**	20	344 - 350	1	1	0	1	2	0.19	0.55	0.82
**36**	**Leg223**	19	326 - 360	0	0	0	0	1	0.00	0.00	0.00
**37**	**PvSHP1**	20	779 - 790	21	21	21	0	3	0.20	2.73	7.70
	**Mean**	**19.8**	**246.4 - 253.6**	**3.1**	**3.1**	**1.0**	**2.1**	**1.9**	**0.2**	**3.45**	**3.24**
	**Overall**	**/**	**9,116 - 9,382**	**114**	**115**	**36**	**78**	**/**	**/**	**/**	**/**

N, sample size; bp. sequence length (base pairs); V, variable sites; η, total number of mutations; S, singleton variable sites; Pi. parsimony informative variable sites; H, number of haplotypes; Hd, haplotype diversity; $\pi \times 10^{-3}$ and $\Theta \times 10^{-3}$, two measure of nucleotide diversity from Tajima (1983) and Watterson (1975), respectively.
