# Supplementary material for: High Level of Nonsynonymous Changes in Common Bean Suggests That Selection under Domestication Increased Functional Diversity at Target Traits
Source: Front Plant Sci. 2017 Jan 6;7:2005. doi: 10.3389/fpls.2016.02005 (PMC5216878; doi:10.3389/fpls.2016.02005)
Supplement: Supplementary file 8 [file Table8.PDF]

Table S8. Summary of the genetic diversity estimates computed for *P. vulgaris* samples and *P. vulgaris* Mesoamerican accessions, for the whole sequences and the coding (exons) and noncoding (introns, 3'UTR, 5'UTR) regions separately.

**Accessions**	**Sequence**	**N. loci**	**N[1]**	**Range bp[2]**	**V[2]**	**η[2]**	**S[2]**	**Pi[2]**	**Syn[2]**	**Nonsyn[2]**	**H[1]**	**Hd[1]**	**π[1] ×10^{-3}**	**Θ[1] ×10^{-3}**
P. vulgaris accessions	Whole sequence	49	44.2	21,514-21,894	465	475	76	389	/	/	6.0	0.54	5.02	4.79
	Coding regions	42	44.4	11,198	151	153	33	118	93	58	3.8	0.37	3.12	2.86
	Introns	32	44.4	7,655-8,004	246	254	38	208	/	/	4.7	0.43	7.04	7.11
	5'UTR	2	44.0	564-565	10	10	1	9	/	/	6.0	0.56	4.02	3.98
	3'UTR	7	45.0	876-896	16	16	1	15	/	/	2.6	0.33	5.11	4.08
	Overall (Introns+5'3'UTR)	37	44.4	9,096-9,463	272	280	40	232	/	/	4.8	0.44	7.00	6.82
Mesoamerican *P. vulgaris* accessions	Whole sequence	49	38.3	21,532-21,865	425	432	100	325	/	/	5.3	0.48	4.42	4.50
	Coding regions	42	38.5	11,198	141	142	46	95	85	56	3.4	0.33	2.72	2.66
	Introns	32	38.4	7,673-7,923	222	228	48	174	/	/	4.2	0.38	6.28	6.40
	5'UTR	2	38.0	564-565	8	8	1	7	/	/	5.0	0.47	3.06	3.27
	3'UTR	7	39.0	879-896	16	16	1	15	/	/	2.6	0.31	5.36	4.23
	Overall (Introns+5'3'UTR)	37	38.5	9,116-9,382	246	252	50	196	/	/	4.3	0.40	6.37	6.27

[1]Average estimate among loci: *N*, sample size; *H*, number of haplotypes; *Hd*, haplotype diversity; $\pi \times 10^{-3}$ and $\Theta \times 10^{-3}$, two measures of nucleotide diversity from Tajima (1983) and Watterson (1975), respectively.

[2]Sum of the single locus estimates: range bp, sequence length (base pairs); *V*, variable sites; *η*, total number of mutations; *S*, singleton variable sites; *Pi*, parsimony informative variable sites; *Syn*, total number of synonymous changes; *Nonsyn*, total number of replacement changes.
