# Supplementary material for: High Level of Nonsynonymous Changes in Common Bean Suggests That Selection under Domestication Increased Functional Diversity at Target Traits
Source: Front Plant Sci. 2017 Jan 6;7:2005. doi: 10.3389/fpls.2016.02005 (PMC5216878; doi:10.3389/fpls.2016.02005)
Supplement: Supplementary file 9 [file Table9.PDF]

**Table S9.** Analysis of molecular variance (AMOVA).

| **Sources of variation** | **df** | **SS** | **MS** | **Variance component** | **%Variation** | **$F_{ST}$** |
|---|---|---|---|---|---|---|
| Among populations | 1 | 246.06 | 246.06 | 9.89 | 16 | 0.16*** |
| Within populations | 37 | 1,975.56 | 53.39 | 53.39 | 84 | |
| Total | 38 | 2,221.62 | | 63.28 | 100 | |

df, degrees of freedom; SS, sum of squares; MS, mean sum of squares; ***P <0.0001.
