# Supplementary material for: High Level of Nonsynonymous Changes in Common Bean Suggests That Selection under Domestication Increased Functional Diversity at Target Traits
Source: Front Plant Sci. 2017 Jan 6;7:2005. doi: 10.3389/fpls.2016.02005 (PMC5216878; doi:10.3389/fpls.2016.02005)
Supplement: Supplementary file 10 [file Table10.PDF]

Table S10. Relative rates of non-synonymous (*dN*) and synonymous (*dS*) substitutions with standard errors (SE) calculated for each of the 42 loci, including exons for the MW and MD populations.

**Locus**	**Variable**											
	**MW**						**MD**					
	N	***dS***	***SE*(*dS*)**	***dN***	***SE*(*dN*)**	**(*dN*-*dS*)**	***N***	***dS***	***SE*(*dS*)**	***dN***	***SE*(*dN*)**	**(*dN*-*dS*)**
AN-Pv1	19	0.005	0.004	0.003	0.002	-0.002	20	/	/	/	/	/
AN-Pv2	19	0.005	0.005	0.000	0.000	-0.005	20	0.003	0.003	0.000	0.000	-0.003
AN-Pv3	19	0.003	0.002	0.004	0.003	0.001	20	0.000	0.000	0.002	0.002	0.002
AN-Pv4	19	/	/	/	/	/	20	/	/	/	/	/
AN-Pv5	19	/	/	/	/	/	20	/	/	/	/	/
AN-Pv8	19	0.021	0.009	0.000	0.000	-0.021	19	0.002	0.002	0.001	0.001	-0.001
AN-Pv9	19	/	/	/	/	/	20	/	/	/	/	/
AN-Pv10	19	0.017	0.012	0.005	0.003	-0.012	20	0.018	0.013	0.005	0.004	-0.013
AN-Pv17	19	/	/	/	/	/	20	/	/	/	/	/
AN-Pv18	19	0.004	0.002	0.002	0.001	-0.002	20	0.000	0.000	0.001	0.001	0.001
AN-Pv22	18	0.012	0.006	0.002	0.001	-0.010	19	0.007	0.005	0.002	0.002	-0.005
AN-Pv26.1	19	0.014	0.009	0.006	0.004	-0.008	20	0.002	0.002	0.001	0.001	-0.001
AN-Pv28	19	0.020	0.021	0.000	0.000	-0.020	20	0.020	0.023	0.000	0.000	-0.020
AN-Pv29	19	0.000	0.000	0.007	0.007	0.007	20	0.000	0.000	0.002	0.002	0.002
AN-Pv30	19	0.018	0.011	0.004	0.003	-0.014	20	0.014	0.010	0.003	0.003	-0.011
AN-Pv32	19	/	/	/	/	/	20	/	/	/	/	/
AN-Pv33	19	0.014	0.009	0.002	0.002	-0.012	20	/	/	/	/	/
AN-Pv35	19	/	/	/	/	/	20	/	/	/	/	/
AN-Pv44	19	0.005	0.004	0.001	0.001	-0.004	20	0.003	0.002	0.000	0.000	-0.003
AN-Pv46	19	0.000	0.000	0.005	0.003	0.005	20	0.000	0.000	0.003	0.002	0.003
AN-Pv47	19	0.012	0.007	0.002	0.001	-0.010	20	0.010	0.006	0.002	0.002	-0.008
AN-Pv51	17	0.019	0.008	0.000	0.000	-0.019	20	0.023	0.010	0.000	0.000	-0.023
AN-Pv54	16	0.017	0.008	0.000	0.000	-0.017	20	0.024	0.010	0.000	0.000	-0.024
AN-Pv55	19	/	/	/	/	/	20	/	/	/	/	/
AN-Pv57	19	0.008	0.003	0.000	0.000	-0.008	20	0.004	0.003	0.000	0.000	-0.004
AN-Pv63	19	0.005	0.003	0.004	0.003	-0.001	20	0.005	0.003	0.003	0.002	-0.002
AN-Pv64	19	/	/	/	/	/	20	/	/	/	/	/
AN-Pv66	19	0.000	0.000	0.002	0.001	0.002	20	/	/	/	/	/
AN-Pv68	19	0.005	0.003	0.003	0.001	-0.002	20	0.002	0.002	0.002	0.002	0.000
AN-Pv69	19	0.006	0.006	0.000	0.000	-0.006	20	/	/	/	/	/
gssE18	19	0.027	0.026	0.011	0.011	-0.016	20	0.010	0.010	0.004	0.004	-0.006
gssE20	19	/	/	/	/	/	20	/	/	/	/	/
AN-PvCO	15	0.011	0.006	0.002	0.001	-0.009	17	0.007	0.004	0.002	0.001	-0.005
AN-TGA	19	0.002	0.003	0.000	0.000	-0.002	17	/	/	/	/	/
AN-DNAJ	19	0.015	0.006	0.000	0.000	-0.015	20	0.002	0.001	0.000	0.000	-0.002
g510	19	0.013	0.008	0.007	0.004	-0.006	20	0.004	0.003	0.003	0.001	-0.001
g523	19	0.005	0.004	0.000	0.000	-0.005	20	0.003	0.003	0.000	0.000	-0.003
Leg044	18	/	/	/	/	/	20	/	/	/	/	/
Leg100	19	/	/	/	/	/	20	/	/	/	/	/
Leg133	19	0.003	0.003	0.000	0.000	-0.003	20	/	/	/	/	/

Leg223	17	/	/	/	/	/	19	/	/	/	/	/
PvSHP1	19	0.000	0.000	0.002	0.001	0.002	20	/	/	/	/	/
