# Supplementary material for: High Level of Nonsynonymous Changes in Common Bean Suggests That Selection under Domestication Increased Functional Diversity at Target Traits
Source: Front Plant Sci. 2017 Jan 6;7:2005. doi: 10.3389/fpls.2016.02005 (PMC5216878; doi:10.3389/fpls.2016.02005)
Supplement: Supplementary file 11 [file Table11.PDF]

Table S11. P-values for each locus analyzed. **Bold**, loci identified as putatively under selection.

**Gene**[1]	**Exon**[2]	**Intron**[2]	**Exon length**	**Intron length**	**Q-value**			
					**Model 1**		**Model 2**	
					**Exon**	**Intron**	**Exon**	**Intron**
AN-Pv1	X	X	230	12	0.35	0.17	0.37	0.15
AN-Pv2	X	-	352	-	0.60	-	0.66	-
AN-Pv3	X	X	234	147	0.51	0.29	0.63	0.37
AN-Pv4	X[3]	X	259	162	-	0.37	-	0.42
AN-Pv5	X[3]	X	259	229	-	0.16	-	0.15
AN-Pv8	X	-	387	-	0.37	-	0.39	-
AN-Pv9	X[3]	X[4]	233	177	-	-	-	-
AN-Pv10	X	X	214	131	0.60	0.42	0.66	0.50
AN-Pv17	X[3]	X	115	223	-	0.15	-	0.15
AN-Pv18	X	-	409	-	0.51	-	0.63	-
AN-Pv22	X	X	340	106	0.06	**0.03**	**0.03**	**0.02**
AN-Pv26_1	X	X	205	234	**0.01**	**0.00**	**0.01**	**0.00**
AN-Pv28	X	X	88	236	0.60	0.60	0.66	0.66
AN-Pv29	X	X	79	168	0.60	0.60	0.66	0.66
AN-Pv30	X	-	216	-	0.22	-	0.17	-
AN-Pv32	X[3]	X[3]	138	146	-	-	-	-
AN-Pv33	X	-	225	-	**0.03**	-	**0.03**	-
AN-Pv35	X[3]	X	46	55	-	0.29	-	0.35
AN-Pv41	-	X	-	134	-	0.58	-	0.66
AN-Pv44	X	X[3]	344	109	0.65	-	0.73	-
AN-Pv46	X	-	381	-	0.20	-	0.15	-
AN-Pv47	X	X	439	100	0.22	0.10	0.17	0.07
AN-Pv51	X	X	319	383	0.60	0.50	0.66	0.56
AN-Pv54	X	-	399	-	0.60	-	0.66	-
AN-Pv55	X[3]	X[3]	391	212	-	-	-	-
AN-Pv57	X	X	242	217	0.70	0.98	0.75	0.99
AN-Pv63	X	-	601	-	0.29	-	0.27	-
AN-Pv64	X[3]	X	230	309	-	0.07	-	**0.05**
AN-Pv66	X	-	266	-	0.37	-	0.42	-
AN-Pv68	X	X	523	206	0.60	0.65	0.68	0.75
AN-Pv69	X	X[4]	274	129	0.06	-	**0.04**	-
gssE18	X	X	71	202	0.15	0.11	0.11	0.08
gssE20	X[3]	X[4]	81	198	-	-	-	-
AN-PvCO	X	X	539	112	0.61	0.50	0.68	0.56
AN-TGA	X	X	173	409	0.53	0.06	0.66	**0.04**
AN-DNAJ	X	-	598	-	**0.03**	-	**0.02**	-
g510	X	X	342	181	0.10	0.61	0.07	0.73
g523	X	-	363	-	**0.03**	-	**0.02**	-
Leg044	X[3]	X	103	765	-	0.47	-	0.56

Leg100	X[3]	X	48	518	-	0.29	-	0.27
Leg133	X	X	241	345	0.35	**0.02**	0.37	**0.01**
Leg223	X[3]	X	146	322	-	**0.02**	-	**0.01**
PvSHP1	X	X	97	763	0.54	0.09	0.66	**0.05**

[1]Selection analysis was carried out for 39 loci, as those including exon and/or intron regions and polymorphic in these regions; [2]X and - indicate the presence and absence of exon and/or intron regions within the locus sequence, respectively; [3]Monomorphic exon/intron regions between Mesoamerican wild and domesticated forms; [4]Exon/intron regions monomorphic between Mesoamerican wild and domesticated forms after gap removal.
