# Supplementary material for: High Level of Nonsynonymous Changes in Common Bean Suggests That Selection under Domestication Increased Functional Diversity at Target Traits
Source: Front Plant Sci. 2017 Jan 6;7:2005. doi: 10.3389/fpls.2016.02005 (PMC5216878; doi:10.3389/fpls.2016.02005)
Supplement: Supplementary file 12 [file Table12.PDF]

Table S12. Comparisons of the neutrality test results from different studies (the present study, Bellucci et al. 2014, Schmutz et al. 2014).

	Locus	Chr.	MODEL 1: Loci identified as putatively neutral (PN) or under selection (PS) in the present study		MODEL 2: Loci identified as putatively neutral (PN) or under selection (PS) in the present study		Loci putatively under selection (PS) in Bellucci et al. (2014)	10-kb/2-kb selection windows and domestication candidate genes in both the Mesoamerican and Andean gene pools in Schmutz et al. (2014)			
			Exons[1]	Introns[1]	Exons[1]	Introns[1]	Mesoamerican candidate genes[2]	Andean selection block	Andean candidate genes	Masoamerican selection block	Masoamerican candidate genes
1	AN-Pv1	Chr07	PN	PN	PN	PN		**443**		**546**	**Phvul.007G099600**
2	AN-Pv2	Chr06	PN		PN		PN				
3	AN-Pv3	Chr09	PN	PN	PN	PN					
4	AN-Pv4	Chr09		PN		PN	PN				
5	AN-Pv5	Chr07		PN		PN	PN	**511**	**Phvul.007G177200**	**597**	
6	AN-Pv8	Chr04	PN		PN		PN			**341**	
7	AN-Pv9	Chr07						**491**			
8	AN-Pv10	Chr10	PN	PN	PN	PN					
9	AN-Pv16	Chr07						**431**		**534**	
10	AN-Pv17	Chr07		PN		PN	PN				
11	AN-Pv18	Chr07	PN		PN						
12	AN-Pv22	Chr02	PN	**PS**	**PS**	**PS**	PN	**232**			
13	AN-Pv26.1	Chr01	**PS**	**PS**	**PS**	**PS**	PN			**95**	
14	AN-Pv28	Chr08	PN	PN	PN	PN	PN				
15	AN-Pv29	Chr09	PN	PN	PN	PN					
16	AN-Pv30	Chr02	PN		PN		PN				
17	AN-Pv32	Chr02					PN	**251**			

18	AN-Pv33	Chr01	**PS**		**PS**					**62**	**Phvul.001G143100**
19	AN-Pv35	Chr07		PN		PN					
20	AN-Pv41	na		PN		PN				**/**	**/**
21	AN-Pv42	Chr02									
22	AN-Pv44	Chr05	PN		PN						
23	AN-Pv46	Chr07	PN		PN						
24	AN-Pv47	Chr11	PN	PN	PN	PN	PN				
25	AN-Pv48	Chr10									
26	AN-Pv51	Chr08	PN	PN	PN	PN	PN				
27	AN-Pv54	Chr07	PN		PN		PN				
28	AN-Pv55	Chr11					PN				
29	AN-Pv57	Chr11	PN	PN	PN	PN	PN				
30	AN-Pv63	Chr01	PN		PN		PN				
31	AN-Pv64	Chr11		PN		**PS**					
32	AN-Pv66	Chr07	PN		PN		PN				**Phvul.007G113700**
33	AN-Pv68	Chr06	PN	PN	PN	PN	PN				
34	AN-Pv69	Chr02	PN		**PS**		**Ref_259_comp14324**		**Phvul.002G242000**		
35	gssE18	Chr01	PN	PN	PN	PN		**41**			
36	gssE19	Chr10						**640**			
37	gssE20	Chr09								**790**	
38	gssE28	Chr01									
39	AN-PvCO	Chr09	PN	PN	PN	PN	PN				
40	AN-TGA	Chr01	PN	PN	PN	**PS**					
41	AN-DNAJ	Chr02	**PS**		**PS**		PN		**Phvul.002G257300**		
42	g510	Chr01	PN	PN	PN	PN	PN			**98**	
43	g523	Chr01	**PS**		**PS**						
44	Leg044	Chr06		PN		PN					

45	Leg100	Chr11		PN		PN	PN		
46	Leg133	Chr11	PN	**PS**	PN	**PS**	PN	**879**	
47	Leg223	Chr09		**PS**		**PS**	**Ref_25_comp4672**	**814**	**Phvul.009G231900**
48	Leg443	Chr08							
49	PvSHP1	Chr06	PN	PN	PN	**PS**			

na, not available; Chr., Chromosome; PN, putatively neutral loci; PS, putatively under selection loci.

[1]Empty cells indicate that the exon and/ or intron part of the gene fragment was not included in the analysis, because: (i) no structure was available (AN-Pv48, gssE19, gssE28, Leg443); (ii) loci are monomorphic in the entire sample (AN-Pv9, AN-Pv32, AN-Pv55, gssE20); (iii) only 5'UTR (AN-Pv16) or 3'UTR (AN-Pv42) regions characterize the gene fragment;

[2]Empty cells indicate gene fragments that gave no matches with the reference transcriptome from Bellucci et al. (2014).
