# Supplementary material for: High Level of Nonsynonymous Changes in Common Bean Suggests That Selection under Domestication Increased Functional Diversity at Target Traits
Source: Front Plant Sci. 2017 Jan 6;7:2005. doi: 10.3389/fpls.2016.02005 (PMC5216878; doi:10.3389/fpls.2016.02005)

Figure S1. Genomic distribution of 47 loci mapped on the reference genome (Schumtz et al., 2014). Dark red and gray boxes indicate centromeric and pericentromeric regions, respectively.

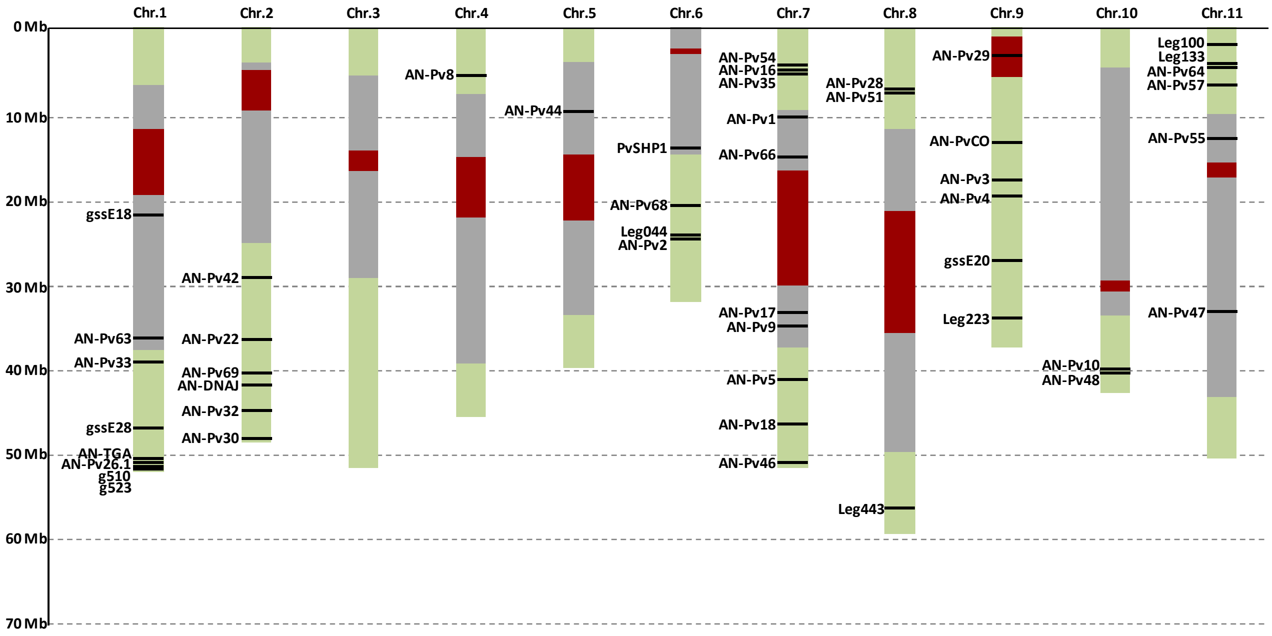

Supplement: Supplementary file 14 [file Image1.pdf]
