# Supplementary material for: High Level of Nonsynonymous Changes in Common Bean Suggests That Selection under Domestication Increased Functional Diversity at Target Traits
Source: Front Plant Sci. 2017 Jan 6;7:2005. doi: 10.3389/fpls.2016.02005 (PMC5216878; doi:10.3389/fpls.2016.02005)
Supplement: Supplementary file 15 [file Image2.pdf]

AN-Pv41

>001_W_M_mx_nt [G2771]

GAATGACTGTCAAATTGTTTGTCATAACAAAGTGCAAGAAGAGATGTAAGGTGAGGATTTGGT

TAAGTT---------------

TTGGATTTTTTTTTTATACATAGAGATAGTAGATAATTCAAACGCATGAGTTCTAATTTTTTTTTA

>003_L_M_vn_df [G5191]

GAATGACTGTCAAATTGTTTGTCATAACAAAGTGCAAGAAGAGATGTAAGGTGAGGATTTGGT

TAAGTTAAATTTTTATAAACCTTGGATTTTTTTTTTATACATAGAGATAGTAGATAATTCAAACGC

ATGAGTTCTAAATTTTTTTTA

>007_W_M_mx_ja [G9989]

GAATGACTGTCAAATTGTTTGTCATAACAAAGTGCAAGAAGAGATGTAAGGTGAGGATTTGGT

TAAGTTAAATTTTTATAAACCTTGGATTTTTTTTTTATACATAGAGATAGTAGATAATTCAAACGC

ATGAGTTCTAAATTTTTTTTA

>010_W_M_mx_mi [G11050]

GAATGACTGTCAAATTGTTTGTCATAACAAAGTGCAAGAAGAGATGTAAGGTGAGGATTTGGT

TAAGTT---------------TTGGA-

TTTTTTTTTATACATAGAGATAGTAGATAATTCAAACGCATGAGTTCTAAATTTTTTTTA

>011_W_M_mx_ja [G11051]

GAATGACTGTCAAATTGTTTGTCATAACAAAGTGCAAGAAGAGATGTAAGGTGAGGATTTGGT

TAAGTT---------------

TTGGATTTTTTTTTTATACATAGAGATAGTAGATAATTCAAACGCATGAGTTCTAAATTTTTTTTA

>012_W_M_mx_ja [G11056]

GAATGACTGTCAAATTGTTTGTCATAACAAAGTGCAAGAAGAGATGTAAGGTGAGGATTTGGT

TAAGTT---------------TTGGA--

TTTTTTTTATACATAGAGATAGTAGATAATTCAAACGCATGAGTTCTAAATTTTTTTTA

>025_W_M_mx_ja [G12979]

GAATGACTGTCAAATTGTTTGTCATAACAAAGTGCAAGAAGAGATGTAAGGTGAGGATTTGGT

TAAGTT---------------TTGGA--

TTTTTTTTATACATAGAGATAGTAGATAATTCAAACGCATGAGTTCTAAATTTTTTTTA

>054_W_PhI_pr_cj [G21245]

GAATGACTGTCAAATTGTTTGTCATAACAAAGTGCAAGAAGAGATGTAAGGTGAGGATTTGGT

TAAGTTAAATTTTTATAAACCTTGGA-

TTTTTTTTTATACATAGAGAGAGTAGATAATTCAAACGCATGAGTTCTAAATTTTTTTTA

>057_W_M_mx_ci [G22837]

GAATGACTGTCAAATTGTTTGTCATAACAAAGTGCAAGAAGAGATGTAAGGTGAGGATTTGGT

TAAGTT---------------

TTGGATTTTTTTTTTATACATAGAGATAGTAGATAATTCAAACGCATGAGTTCTAAATTTTTTTTA

>065_W_M_mx_pu [G23429]

GAATGACTGTCAAATTGTTTGTCATAACAAAGTGCAAGAAGAGATGTAAGGTGAGGATTTGGT

TAAGTT---------------TTGGA-

TTTTTTTTTATACATAGAGATAGTAGATAATTCAAACGCATGAGTTCTAAATTTTTTTTA

>073_W_PhI_ec_cm [G23582]

GAATGACTGTCAAATTGTTTGTCATAACAAAGTGCAAGAAGAGATGTAAGGTGAGGATTTGGT

TAAGTTAAATTTTTATAAACCTTGGA-

TTTTTTTTTATACATAGAGAGAGTAGATAATTCAAACGCATGAGTTCTAAATTTTTTTTA

>076_W_M_mx_pu [G23652]

GAATGACTGTCAAATTGTTTGTCATAACAAAGTGCAAGAAGAGATGTAAGGTGAGGATTTGGT

TAAGTT---------------TTGGA-

TTTTTTTTTATACATAGAGATAGTAGATAATTCAAACGCATGAGTTCTAAATTTTTTTTA

>080_W_M_mx_ox [G24387]

GAATGACTGTCAAATTGTTTGTCATAACAAAGTGCAAGAAGAGATGTAAGGTGAGGATTTGGT

TAAGTTAAATTTTTATAAACCTTGGA-

TTTTTTTTTATACATAGAGAGAGTAGATAATTCAAACGCATGAGTTCTAAATTTTTTTTA

>082_W_M_mx_ox [G24572]

GAATGACTGTCAAATTGTTTGTCATAACAAAGTGCAAGAAGAGATGTAAGGTGAGGATTTGGT

TAAGTT---------------

TTGGATTTTTTTTTTATACATAGAGATAGTAGATAATTCAAACGCATGAGTTCTAAATTTTTTTTA

>085_W_M_mx_du [G50899]

GAATGACTGTCAAATTGTTTGTCATAACAAAGTGCAAGAAGAGATGTAAGGTGAGGATTTGGT

TAAGTTAAATTTTTATAAACCTTGGA--

TTTTTTTTATACATAGAGAGAGTAGATAATTCAAACGCATGAGTTCTAAATTTTTTTTA

>089_L_M_cl_mt [PI151017]

GAATGACTGTCAAATTGTTTGTCATAACAAAGTGCAAGAAGAGATGTAAGGTGAGGATTTGGT

TAAGTT---------------

TTGGATTTTTTTTTTATACATAGAGATAGTAGATAATTCAAACGCATGAGTTCTAAATTTTTTTTA

>102_L_M_mx_pu_D [PI165440]

GAATGACTGTCAAATTGTTTGTCATAACAAAGTGCAAGAAGAGATGTAAGGTGAGGATTTGGT

TAAGTTAAATTTTTATAAACCTTGGATTTTTTTTTTATACATAGAGATAGTAGATAATTCAAACGC

ATGAGTTCTAAATTTTTTTTA

>104_L_M_mx_vr_D [PI196933]

GAATGACTGTCAAATTGTTTGTCATAACAAAGTGCAAGAAGAGATGTAAGGTGAGGATTTGGT

TAAGTT---------------

TTGGATTTTTTTTTTATACATAGAGATAGTAGATAATTCAAACGCATGAGTTCTAATTTTTTTTTA

>106_Pd_L_mx_pu

GAATGACTGTCAAATTGTTTGTCATAACAAAGTGCAAGAAGAGATGTAAGGTGAGGATTTGGT

TAAGTTAAATTTTTATAAACCTTGGA--

TTTTTTGTATACATAGATATAGTAGATAATTCAAACGCATGAGTTCTAAATTTTTTTTA

>107_L_M_mx_pu [PI201349]

GAATGACTGTCAAATTGTTTGTCATAACAAAGTGCAAGAAGAGATGTAAGGTGAGGATTTGGT

TAAGTTAAATTTTTATAAACCTTGGATTTTTTTTTTATACATAGAGATAGTAGATAATTCAAACGC

ATGAGTTCTAAATTTTTTTTA

>118_L_M_mx_ja [PI281981]

GAATGACTGTCAAATTGTTTGTCATAACAAAGTGCAAGAAGAGATGTAAGGTGAGGATTTGGT

TAAGTTAAATTTTTATAAACCTTGGATTTTTTTTTTATACATAGAGATAGTAGATAATTCAAACGC

ATGAGTTCTAAATTTTTTTTA

>136_L_M_cl [PI300668]

GAATGACTGTCAAATTGTTTGTCATAACAAAGTGCAAGAAGAGATGTAAGGTGAGGATTTGGT

TAAGTTAAATTTTTATAAACCTTGGATTTTTTTTTTATACATAGAGATAGTAGATAATTCAAACGC

ATGAGTTCTAAATTTTTTTTA

>145_L_M_mx [PI309755]

GAATGACTGTCAAATTGTTTGTCATAACAAAGTGCAAGAAGAGATGTAAGGTGAGGATTTGGT

TAAGTT---------------

TTGGATTTTTTTTTTATACATAGAGATAGTAGATAATTCAAACGCATGAGTTCTAAATTTTTTTTA

>146_L_M_mx_ja [PI309785]

GAATGACTGTCAAATTGTTTGTCATAACAAAGTGCAAGAAGAGATGTAAGGTGAGGATTTGGT

TAAGTT---------------

TTGGATTTTTTTTTTATACATAGAGATAGTAGATAATTCAAACGCATGAGTTCTAAATTTTTTTTA

>148_L_M_cr_ct [PI309831]

GAATGACTGTCAAATTGTTTGTCATAACAAAGTGCAAGAAGAGATGTAAGGTGAGGATTTGGT

TAAGTTAAATTTTTATAAACCTTGGATTTTTTTTTTATACATAGAGATAGTAGATAATTCAAACGC

ATGAGTTCTAAATTTTTTTTA

>156_L_M_gt_ca_G [PI310660]

GAATGACTGTCAAATTGTTTGTCATAACAAAGTGCAAGAAGAGATGTAAGGTGAGGATTTGGT

TAAGTT---------------

TTGGATTTTTTTTTTATACATAGAGATAGTAGATAATTCAAACGCATGAGTTCTAAATTTTTTTTA

>159_L_M_es_st [PI311794]

GAATGACTGTCAAATTGTTTGTCATAACAAAGTGCAAGAAGAGATGTAAGGTGAGGATTTGGT

TAAGTTAAATTTTTATAAACCTTGGATTTTTTTTTTATACATAGAGATAGTAGATAATTCAAACGC

ATGAGTTCTAAATTTTTTTTA

>162_L_M_mx_so_D [PI312092]

GAATGACTGTCAAATTGTTTGTCATAACAAAGTGCAAGAAGAGATGTAAGGTGAGGATTTGGT

TAAGTT---------------

TTGGATTTTTTTTTTATACATAGAGATAGTAGATAATTCAAACGCATGAGTTCTAAATTTTTTTTA

>165_L_M_mx_ag_D [PI313214]

GAATGACTGTCAAATTGTTTGTCATAACAAAGTGCAAGAAGAGATGTAAGGTGAGGATTTGGT

TAAGTT---------------

TTGGATTTTTTTTTTATACATAGAGATAGTAGATAATTCAAACGCATGAGTTCTAAATTTTTTTTA

>166_L_M_mx_du_D [PI313301]

GAATGACTGTCAAATTGTTTGTCATAACAAAGTGCAAGAAGAGATGTAAGGTGAGGATTTGGT

TAAGTT---------------

TTGGATTTTTTTTTTATACATAGAGATAGTAGATAATTCAAACGCATGAGTTCTAAATTTTTTTTA

>168_L_M_mx_mi_D [PI313469]

GAATGACTGTCAAATTGTTTGTCATAACAAAGTGCAAGAAGAGATGTAAGGTGAGGATTTGGT

TAAGTT---------------

TTGGATTTTTTTTTTATACATAGAGATAGTAGATAATTCAAACGCATGAGTTCTAAATTTTTTTTA

>175_L_M_mx_gj_D [PI313755]

GAATGACTGTCAAATTGTTTGTCATAACAAAGTGCAAGAAGAGATGTAAGGTGAGGATTTGGT

TAAGTTAAATTTTTATAAACCTTGGATTTTTTTTTTATACATAGAGATAGTAGATAATTCAAACGC

ATGAGTTCTAAATTTTTTTTA

>179_W_M_mx_ja [PI318696]

GAATGACTGTCAAATTGTTTGTCATAACAAAGTGCAAGAAGAGATGTAAGGTGAGGATTTGGT

TAAGTT---------------

TTGGATTTTTTTTTTATACATAGAGATAGTAGATAATTCAAACGCATGAGTTCTAAATTTTTTTTA

>187_W_M_mx_mo [PI325677]

GAATGACTGTCAAATTGTTTGTCATAACAAAGTGCAAGAAGAGATGTAAGGTGAGGATTTGGT

TAAGTT---------------TTGGA-

TTTTTTTTTATACATAGAGATAGTAGATAATTCAAACGCATGAGTTCTAAATTTTTTTTA

>202_Pc_W_mx_ja [PI417608]

GAATGACTGTCAAATTGTTTGTCATAACAAAGTGCAAGAAGAGATGTAAGGTGAGGATTTGGT

TAAGTTAAATTTTTATAAACCTTGGA-

TTTTTTTTTATACTTAGAGATAGTAGATAATTCAAACGCATGAGTTCTAAATTTTTTTTA

>204_L_M_mx_ja [PI417760]

GAATGACTGTCAAATTGTTTGTCATAACAAAGTGCAAGAAGAGATGTAAGGTGAGGATTTGGT

TAAGTT---------------

TTGGATTTTTTTTTTATACATAGAGATAGTAGATAATTCAAACGCATGAGTTCTAAATTTTTTTTA

>205_W_M_mx_ja [PI417775]

GAATGACTGTCAAATTGTTTGTCATAACAAAGTGCAAGAAGAGATGTAAGGTGAGGATTTGGT

TAAGTT---------------TTGGA-

TTTTTTTTTATACATAGAGATAGTAGATAATTCAAACGCATGAGTTCTAAATTTTTTTTA

>220_W_A_ar_sa [W617475]

GAATGACTGTCAAATTGTTTGTCATAACAAAGTGCAAGAAGAGATGTAAGGTGAGGATTTGGT

TAAGTT---------------TTGGA--

TTTTTTTTATACATAGAGATAGTAGATAATTCAAACGCATGAGTTCTAAATTTTTTTTA

>242_W_A_bl_cq [W618821]

GAATGACTGTCAAATTGTTTGTCATAACAAAGTGCAAGAAGAGATGTAAGGTGAGGATTTGGT

TAAGTT---------------TTGGA--

TTTTTTTTATACATAGAGATAGTAGATAATTCAAACGCATGAGTTCTAAATTTTTTTTA

>259_W_M_mx_mo [G12873]

GAATGACTGTCAAATTGTTTGTCATAACAAAGTGCAAGAAGAGATGTAAGGTGAGGATTTGGT

TAAGTT---------------TTGGA-

TTTTTTTTTATACATAGAGATAGTAGATAATTCAAACGCATGAGTTCTAAATTTTTTTTA

>260_C_M_co_M [BAT93]

GAATGACTGTCAAATTGTTTGTCATAACAAAGTGCAAGAAGAGATGTAAGGTGAGGATTTGGT

TAAGTTAAATTTTTATAAACCTTGGATTTTTTTTTTATACATAGAGATAGTAGATAATTCAAACGC

ATGAGTTCTAAATTTTTTTTA

>261_L_A_br_NG [JaloEEP558]

GAATGACTGTCAAATTGTTTGTCATAACAAAGTGCAAGAAGAGATGTAAGGTGAGGATTTGGT

TAAGTT---------------TTGGA--

TTTTTTTTATACATAGAGATAGTAGATAATTCAAACGCATGAGTTCTAAATTTTTTTTA

>262_L_A_ar [MIDAS]

GAATGACTGTCAAATTGTTTGTCATAACAAAGTGCAAGAAGAGATGTAAGGTGAGGATTTGGT

TAAGTTAAATTTTTATAAACCTTGGATTTTTTTTTTATACATAGAGATAGTAGATAATTCAAACGC

ATGAGTTCTAAATTTTTTTTA

>503_W_M_mx_nt [PI417770]

GAATGACTGTCAAATTGTTTGTCATAACAAAGTGCAAGAAGAGATGTAAGGTGAGGATTTGGT

TAAGTT---------------TTGGA-

TTTTTTTTTATACATAGAGATAGTAGATAATTCAAACGCATGAGTTCTAAATTTTTTTTA

>505_W_M_mx_ja [PI535409]

GAATGACTGTCAAATTGTTTGTCATAACAAAGTGCAAGAAGAGATGTAAGGTGAGGATTTGGT

TAAGTTAAATTTTTATAAACCTTGGA--

TTTTTTTTATACATAGAGATCGTAGATAATTCAAACGCATGAGTTCTAAATTTTTTTTA

>506_W_M_mx_mo [PI535450]

GAATGACTGTCAAATTGTTTGTCATAACAAAGTGCAAGAAGAGATGTAAGGTGAGGATTTGGT

TAAGTTCAATTTTTATAAACCTTGGA--

TTTTTTTTATACATAGAGATAGTAGATAATTCAAACGCATGAGTTCTAAATTTTTTTTA

>522_L_M_mx_ja_J [G1796]

GAATGACTGTCAAATTGTTTGTCATAACAAAGTGCAAGAAGAGATGTAAGGTGAGGATTTGGT

TAAGTT---------------

TTGGATTTTTTTTTTTATACATAGAGATAGTAGATAATTCAAACGCATGAGTTCTAAATTTTTTTTA

AN-Pv42

>001_W_M_mx_nt [G2771]

TCTTTGCACATTAAAGACATACACTAAGCTAAAGCTGCAAGACCACATCGGCTTGCACTA

TCAGAGCTGAGACGCTACCATTGGCCTTGTTTGTAGTGAAAGCTGCACCACGTGGAAAGG

GTATTTCAACTTCCTTTCCACTCTTCGCATGAGTGAAAAGTGTTGAAGTCAACTTGCCCG

ACCACC

>003_L_M_vn_df [G5191]

TTTTTGCACATTAAAGACATACACTAAGCTAAAGCTGCAAGACCACATCGGCTTGCACTA

TCAGAGCTGAGACGCTACCATTGGCCTTGTTTGTAGTGAAAGCTGCTCCACGTGGAAAGG

GTATTTCAACTTCCTTTCCACTCTTCGCATGAGTGAAAAGTGTTGAAGTCAACTTCCCCG

ACCACC

>007_W_M_mx_ja [G9989]

TCTTTGCACATTAAAGACATACACTAAGCTAAAGCTGCAAGACCACATCGGCTTGCACTA

TCAGAGCTGAGACGCTACCATTGGCCTTGTTTGTAGTGAAAGCTGCACCACGTGGAAAGG

GTATTTCAACTTCCTTTCCACTCTTCGCATGAGTGAAAAGTGTTGAAGTCAACTTGCCCG

ACCACC

>010_W_M_mx_mi [G11050]

TCTTTGCACATTAAAGACATACACTAAGCTAAAGCTGCAAGACCACATCGGCTTGCACTA

TCAGAGCTGAGACGCTACCATTGGCCTTGTTTGTAGTGAAAGCTGCACCACGTGGAAAGG

GTATTTCAACTTCCTTTCCACTCTTCGCATGAGTGAAAAGTGTTGAAGTCAACTTGCCCG

ACCACC

>011_W_M_mx_ja [G11051]

TCTTTGCACATTAAAGACATACACTAAGCTAAAGCTGCAAGACCACATCGGCTTGCACTA

TCAGAGCTGAGACGCTACCATTGGCCTTGTTTGTAGTGAAAGCTGCACCACGTGGAAAGG

GTATTTCAACTTCCTTTCCACTCTTCGCATGAGTGAAAAGTGTTGAAGTCAACTTGCCCG

ACCACC

>012_W_M_mx_ja [G11056]

TTTTTGCACATTAAAGACATACACTAAGCTAAAGCTGCAAGACCACATCGGCTTGCACTA

TCAGAGCTGAGACGCTACCATTGGCCTTGTTTGTAGTGAAAGCTGCTCCACGTGGAAAGG

GTATTTCAACTTCCTTTCCACTCTTCGCATGAGTGAAAAGTGTTGAAGTCAACTTCCCCG

ACCACC

>025_W_M_mx_ja [G12979]

TTTTTGCACATTAAAGACATACACTAAGCTAAAGCTGCAAGACCACATCGGCTTGCACTA

TCAGAGCTGAGACGCTACCATTGGCCTTGTTTGTAGTGAAAGCTGCTCCACGTGGAAAGG

GTATTTCAACTTCCTTTCCACTCTTCGCATGAGTGAAAAGTGTTGAAGTCAACTTCCCCG

ACCACC

>054_W_PhI_pr_cj [G21245]

TCTTTGCACATTAAAGACATACACTAAGCTAAAGCTGCAAGACCACATCGGCTTGCACTA

TCAGAGCTGAGACGCTACCATTGGCCTTGTTTGTAGTGAAAGCTGCACCACGTGGAAAGG

GTATTTCAACTTCCTTTCCACTCTTCGCATGAGTGAAAAGTGTTGAAGTCAACTTGCCCG

ACCACC

>057_W_M_mx_ci [G22837]

TCTTTGCACATTAAAGACATACACTAAGCTAAAGCTGCAAGACTACATCGGCTTGCACTA

TCAGAGCTGAGACGCTACCATTGGCCTTGTTTGTAGTGAAAGCTGCACCACGTGGAAAGG

GTATTTCAACTTCCTTTCCACTCTTCGCATGAGTGAAA-GTGTTGAAGTCAACTTGCCCG

ACCACC

>065_W_M_mx_pu [G23429]

TTTTTGCACATTAAAGACATACACTAAGCTAAAGCTGCAAGACCACATCGGCTTGCACTA

TCAGAGCTGAGACGCTACCATTGGCCTTGTTTGTAGTGAAAGCTGCTCCACGTGGAAAGG

GTATTTCAACTTCCTTTCCACTCTTCGCATGAGTGAAAAGTGTTGAAGTCAACTTCCCCG

ACCACC

>073_W_PhI_ec_cm [G23582]

TCTTTGCACATTAAAGACATACACTAAGCTAAAGCTGCAAGACCACATCGGCTTGCACTA

TCAGAGCTGAGACGCTACCATTGGCCTTGTTTGTAGTGAAAGCTGCACCACGTGGAAAGG

GTATTTCAACTTCCTTTCCACTCTTCGCATGAGTGAAAAGTGTTGAAGTCAACTTGCCCG

ACCACC

>076_W_M_mx_pu [G23652]

TTTTTGCACATTAAAGACATACACTAAGCTAAAGCTGCAAGACCACATCGGCTTGCACTA

TCAGAGCTGAGACGCTACCATTGGCCTTGTTTGTAGTGAAAGCTGCTCCACGTGGAAAGG

GTATTTCAACTTCCTTTCCACTCTTCGCATGAGTGAAAAGTGTTGAAGTCAACTTCCCCG

ACCACC

>080_W_M_mx_ox [G24378]

TCTTTGCACATTAAAGACATACACTAAGCTAAAGCTGCAAGACCACATCGGCTTGCACTA

TCAGAGCTGAGACGCTACCATTGG-CTTGTTTGTAGTGAAAGCTGCACCACGTGGAAGGG

GTATTTCAACTTCCTTTCCACTCTTCGCATGAGTGAAAAGTGTTGAAGTCAACTTGCCCG

ACCACC

>082_W_M_mx_ox [G24572]

TCTTTGCACATTAAAGACATACACTAAGCTAAAGCTGCAAGACCACATCGGCTTGCACTA

TCAGAGCTGAGACGCTACCATTGGCCTTGTTTGTAGTGAAAGCTGCACCACGTGGAAAGG

GTATTTCAACTTCCTTTCCACTCTTCGCATGAGTGAAAAGTGTTGAAGTCAACTTGCCCG

ACCACC

>085_W_M_mx_du [G50899]

TCTTTGCACATTAAAGACATACACTAAGCTAAAGCTGCAAGACTACATCGGCTTGCACTA

TCAGAGCTGAGACGCTACCATTGGCCTTGTTTGTAGTGAAAGCTGCACCACGTGGAAAGG

GTATTTCAACTTCCTTTCCACTCTTCGCATGAGTGAAAAGTGTTGAAGTCAACTTGCCCG

ACCACC

>089_L_M_cl_mt [PI151017]

TCTTTGCACATTAAAGACATACACTAAGCTAAAGCTGCAAGACCACATCGGCTTGCACTA

TCAGAGCTGAGACGCTACCATTGGCCTTGTTTGTAGTGAAAGCTGCACCACGTGGAAAGG

GTATTTCAACTTCCTTTCCACTCTTCGCATGAGTGAAAAGTGTTGAAGTCAACTTGCCCG

ACCACC

>102_L_M_mx_pu_D [PI165440]

TCTTTGCACATTAAAGACATACACTAAGCTAAAGCTGCAAGACCACATCGGCTTGCACTA

TCAGAGCTGAGACGCTACCATTGGCCTTGTTTGTAGTGAAAGCTGCACCACGTGGAAAGG

GTATTTCAACTTCCTTTCCACTCTTCGCATGAGTGAAAAGTGTTGAAGTCAACTTGCCCG

ACCACC

>104_L_M_mx_vr_D [PI196933]

TCTTTGCACATTAAAGACATACACTAAGCTAAAGCTGCAAGACCACATCGGCTTGCACTA

TCAGAGCTGAGACGCTACCATTGGCCTTGTTTGTAGTGAAAGCTGCACCACGTGGAAAGG

GTATTTCAACTTCCTTTCCACTCTTCGCATGAGTGAAAAGTGTTGAAGTCAACTTGCCCG

ACCACC

>106_Pd_L_mx_pu [PI201340]

TCTTTGCACATTAAAGACATACACTAAGCTAAAGCTGCAAGACCACATCGGCTTGCACTA

TCAGAGCTGAGACGCTACCATTGGCCTTGTTTGTAGTGAAAGCTGCACCACGTGGAAAGG

GTATTTCAACTTCCTTTCCACTCTTCGCATGAGTGAAAAGTGTTGAAGTCAACTTGCCCG

ACCACC

>107_L_M_mx_pu [PI201349]

TCTTTGCACATTAAAGACATACACTAAGCTAAAGCTGCAAGACCACATCGGCTTGCACTA

TCAGAGCTGAGACGCTACCATTGGCCTTGTTTGTAGTGAAAGCTGCACCACGTGGAAAGG

GTATTTCAACTTCCTTTCCACTCTTCGCATGAGTGAAAAGTGTTGAAGTCAACTTGCCCG

ACCACC

>118_L_M_mx_ja [PI281981]

TCTTTGCACATTAAAGACATACACTAAGCTAAAGCTGCAAGACCACATCGGCTTGCACTA

TCAGAGCTGAGACGCTACCATTGGCCTTGTTTGTAGTGAAAGCTGCACCACGTGGAAAGG

GTATTTCAACTTCCTTTCCACTCTTCGCATGAGTGAAAAGTGTTGAAGTCAACTTGCCCG

ACCACC

>136_L_M_cl [PI300668]

TTTTTGCACATTAAAGACATACACTAAGCTAAAGCTGCAAGACCACATCGGCTTGCACTA

TCAGAGCTGAGACGCTACCATTGGCCTTGTTTGTAGTGAAAGCTGCTCCACGTGGAAAGG

GTATTTCAACTTCCTTTCCACTCTTCGCATGAGTGAAAAGTGTTGAAGTCAACTTCCCCG

ACCACC

>145_L_M_mx [PI309755]

TCTTTGCACATTAAAGACATACACTAAGCTAAAGCTGCAAGACCACATCGGCTTGCACTA

TCAGAGCTGAGACGCTACCATTGGCCTTGTTTGTAGTGAAAGCTGCACCACGTGGAAAGG

GTATTTCAACTTCCTTTCCACTCTTCGCATGAGTGAAAAGTGTTGAAGTCAACTTGCCCG

ACCACC

>146_L_M_mx_ja [PI309785]

TCTTTGCACATTAAAGACATACACTAAGCTAAAGCTGCAAGACCACATCGGCTTGCACTA

TCAGAGCTGAGACGCTACCATTGGCCTTGTTTGTAGTGAAAGCTGCACCACGTGGAAAGG

GTATTTCAACTTCCTTTCCACTCTTCGCATGAGTGAAAAGTGTTGAAGTCAACTTGCCCG

ACCACC

>148_L_M_cr_ct [PI309831]

TTTTTGCACATTAAAGACATACACTAAGCTAAAGCTGCAAGACCACATCGGCTTGCACTA

TCAGAGCTGAGACGCTACCATTGGCCTTGTTTGTAGTGAAAGCTGCTCCACGTGGAAAGG

GTATTTCAACTTCCTTTCCACTCTTCGCATGAGTGAAAAGTGTTGAAGTCAACTTCCCCG

ACCACC

>156_L_M_gt_ca_G [PI310660]

TCTTTGCACATTAAAGACATACACTAAGCTAAAGCTGCAAGACCACATCGGCTTGCACTA

TCAGAGCTGAGACGCTACCATTGGCCTTGTTTGTAGTGAAAGCTGCACCACGTGGAAAGG

GTATTTCAACTTCCTTTCCACTCTTCGCATGAGTGAAAAGTGTTGAAGTCAACTTGCCCG

ACCACC

>159_L_M_es_st [PI311794]

TTTTTGCACATTAAAGACATACACTAAGCTAAAGCTGCAAGACCACATCGGCTTGCACTA

TCAGAGCTGAGACGCTACCATTGGCCTTGTTTGTAGTGAAAGCTGCTCCACGTGGAAAGG

GTATTTCAACTTCCTTTCCACTCTTCGCATGAGTGAAAAGTGTTGAAGTCAACTTCCCCG

ACCACC

>162_L_M_mx_so_D [PI312092]

TCTTTGCACATTAAAGACATACACTAAGCTAAAGCTGCAAGACCACATCGGCTTGCACTA

TCAGAGCTGAGACGCTACCATTGGCCTTGTTTGTAGTGAAAGCTGCACCACGTGGAAAGG

GTATTTCAACTTCCTTTCCACTCTTCGCATGAGTGAAA-GTGTTGAAGTCAACTTGCCCG

ACCACC

>165_L_M_mx_ag_D [PI313214]

TCTTTGCACATTAAAGACATACACTAAGCTAAAGCTGCAAGACCACATCGGCTTGCACTA

TCAGAGCTGAGACGCTACCATTGGCCTTGTTTGTAGTGAAAGCTGCACCACGTGGAAAGG

GTATTTCAACTTCCTTTCCACTCTTCGCATGAGTGAAAAGTGTTGAAGTCAACTTGCCCG

ACCACC

>166_L_M_mx_du_D [PI313301]

TCTTTGCACATTAAAGACATACACTAAGCTAAAGCTGCAAGACCACATCGGCTTGCACTA

TCAGAGCTGAGACGCTACCATTGGCCTTGTTTGTAGTGAAAGCTGCACCACGTGGAAAGG

GTATTTCAACTTCCTTTCCACTCTTCGCATGAGTGAAA-GTGTTGAAGTCAACTTGCCCG

ACCACC

>168_L_M_mx_mi_D [PI313469]

TCTTTGCACATTAAAGACATACACTAAGCTAAAGCTGCAAGACCACATCGGCTTGCACTA

TCAGAGCTGAGACGCTACCATTGGCCTTGTTTGTAGTGAAAGCTGCACCACGTGGAAAGG

GTATTTCAACTTCCTTTCCACTCTTCGCATGAGTGAAAAGTGTTGAAGTCAACTTGCCCG

ACCACC

>175_L_M_mx_gj_D [PI313755]

TCTTTGCACATTAAAGACATACACTAAGCTAAAGCTGCAAGACCACATCGGCTTGCACTA

TCAGAGCTGAGACGCTACCATTGGCCTTGTTTGTAGTGAAAGCTGCACCACGTGGAAAGG

GTATTTCAACTTCCTTTCCACTCTTCGCATGAGTGAAAAGTGTTGAAGTCAACTTGCCCG

ACCACC

>179_W_M_mx_ja [PI318696]

TCTTTGCACATTAAAGACATACACTAAGCTAAAGCTGCAAGACCACATCGGCTTGCACTA

TCAGAGCTGAGACGCTACCATTGGCCTTGTTTGTAGTGAAAGCTGCACCACGTGGAAAGG

GTATTTCAACTTCCTTTCCACTCTTCGCATGAGTGAAAAGTGTTGAAGTCAACTTGCCCG

ACCACC

>187_W_M_mx_mo [PI325677]

TCTTTGCACATTAAAGACATACACTAAGCTAAAGCTGCAAGACCACATCGGCTTGCACTA

TCAGAGCTGAGACGCTACCATTGGCCTTGTTTGTAGTGAAAGCTGCACCACGTGGAAAGG
GTATTTCAACTTCCTTTCCACTCTTCGCATGAGTGAAAAGTGTTGAAGTCAACTTGCCCG
ACCACC
>202_Pc_W_mx_ja [PI417608]
TCTTTGCACATTAAAGACATACACTAAGCTAAAGCTGCAAGACCACATCGGCTTGCATTA
TCAGAGCTGAGACGCTACCATTGGCCTTGTTTGTAGTGAAAGCTGCACCACGTGGAAAGG
GTGTTTCAACTTCCTTTCCACTCTTCGCATGAGTGAAAAGTGTTGAAGTCAACTTGCCCG
ACCACC
>204_L_M_mx_ja [PI417760]
TCTTTGCACATTAAAGACATACACTAAGCTAAAGCTGCAAGACCACATCGGCTTGCACTA
TCAGAGCTGAGACGCTACCATTGGCCTTGTTTGTAGTGAAAGCTGCACCACGTGGAAAGG
GTATTTCAACTTCCTTTCCACTCTTCGCATGAGTGAAAAGTGTTGAAGTCAACTTGCCCG
ACCACC
>205_W_M_mx_ja [PI417775]
TTTTTGCACATTAAAGACATACACTAAGCTAAAGCTGCAAGACCACATCGGCTTGCACTA
TCAGAGCTGAGACGCTACCATTGGCCTTGTTTGTAGTGAAAGCTGCTCCACGTGGAAAGG
GTATTTCAACTTCCTTTCCACTCTTCGCATGAGTGAAAAGTGTTGAAGTCAACTTCCCCG
ACCACC
>220_W_A_ar_sa [W617475]
TCTTTGCACATTAAAGACATACACTAAGCTAAAGCTGCAAGACCACATCGGCTTGCACTA
TCAGAGCTGAGACGCTACCATTGGCCTTGTTTGTAGTGAAAGCTGCACCACGTGGAAAGG
GTATTTCAACTTCCTTTCCACTCTTCGCATGAGTGAAAAGTGTTGAAGTCAACTTGCCCG
ACCACC
>242_W_A_bl_cq [W618821]
TCTTTGCACATTAAAGACATACACTAAGCTAAAGCTGCAAGACCACATCGGCTTGCACTA
TCAGAGCTGAGACGCTACCATTGG-CTTGTTTGTAGTGAAAGCTGCACCACGTGGAAAGG
GTATTTCAACTTCCTTTCCACTCTTCGCATGAGTGAAAAGTGTTGAAGTCAACTTGCCCG
ACCACC

>259_W_M_mx_mo [G12873]

TCTTTGCACATTAAAGACATACACTAAGCTAAAGCTGCAAGACCACATCGGCTTGCACTA

TCAGAGCTGAGACGCTACCATTGGCCTTGTTTGTAGTGAAAGCTGCACCACGTGGAAAGG

GTATTTCAACTTCCTTTCCACTCTTCGCATGAGTGAAAAGTGTTGAAGTCAACTTGCCCG

ACCACC

>260_C_M_co_M [BAT93]

TCTTTGCACATTAAAGACATACACTAAGCTAAAGCTGCAAGACCACATCGGCTTGCACTA

TCAGAGCTGAGACGCTACCATTGGCCTTGTTTGTAGTGAAAGCTGCACCACGTGGAAAGG

GTATTTCAACTTCCTTTCCACTCTTCGCATGAGTGAAAAGTGTTGAAGTCAACTTGCCCG

ACCACC

>261_L_A_br_NG [JaloEEP558]

TCTTTGCACATTAAAGACATACACTAAGCTAAAGCTGCAAGACCACATCGGCTTGCACTA

TCAGAGCTGAGACGCTACCATTGGCCTTGTTTGTAGTGAAAGCTGCACCACGTGGAAAGG

GTATTTCAACTTCCTTTCCACTCTTCGCATGAGTGAAAAGTGTTGAAGTCAACTTGCCCG

ACCACC

>262_L_A_ar [MIDAS]

TCTTTGCACATTAAAGACATACACTAAGCTAAAGCTGCAAGACCACATCGGCTTGCACTA

TCAGAGCTGAGACGCTACCATTGG-CTTGTTTGTAGTGAAAGCTGCACCACGTGGAAAGG

GTATTTCAACTTCCTTTCCACTCTTCGCATGAGTGAAAAGTGTTGAAGTCAACTTGCCCG

ACCACC

>503_W_Mmx_nt [PI417770]

TTTTTGCACATTAAAGACATACACTAAGCTAAAGCTGCAAGACCACATCGGCTTGCACTA

TCAGAGCTGAGACGCTACCATTGGCCTTGTTTGTAGTGAAAGCTGCTCCACGTGGAAAGG

GTATTTCAACTTCCTTTCCACTCTTCGCATGAGTGAAAAGTGTTGAAGTCAACTTCCCCG

ACCACC

>505_W_M_mx_ja [PI535409]

TTTTTGCACATTAAAGACATACACTAAGCTAAAGCTGCAAGACCACATCGGCTTGCACTA

TCAGAGCTGAGACGCTACCATTGGCCTTGTTTGTAGTGAAAGCTGCTCCACGTGGAAAGG

GTATTTCAACTTCCTTTCCACTCTTCGCATGAGTGAAAAGTGTTGAAGTCAACTTCCCCG

ACCACC

>506_W_M_mx_mo [PI535450]

TCTTTGCACATTAAAGACATACACTAAGCTAAAGCTGCAAGACCACATCGGCTTGCACTA

TCAGAGCTGAGACGCTACCATTGG-CTTGTTTGTAGTGAAAGCTGCACCACGTGGAAAGG

GTATTTCAACTTCCTTTCCACTCTTCGCATGAGTGAAAAGTGTTGAAGTCAACTTGCCCG

ACCACC

>522_L_M_mx_ja_J [G1796]

TCTTTGCACATTAAAGACATACACTAAGCTAAAGCTGCAAGACCACATCGGCTTGCACTA

TCAGAGCTGAGACGCTACCATTGGCCTTGTTTGTAGTGAAAGCTGCACCACGTGGAAAGG

GTATTTCAACTTCCTTTCCACTCTTCGCATGAGTGAAAAGTGTTGAAGTCAACTTGCCCG

ACCACC

AN-DNAJ: KY194860 - KY194884
AN-Pv1: KY194885 - KY194909
AN-Pv2: KY194910 - KY194934
AN-Pv3: KY194935 - KY194959
AN-PV4: KY194960 - KY194984
AN-Pv5: KY194985 - KY195009
AN-Pv8: KY195010 - KY195034
AN-Pv9: KY195035 - KY195059
AN-Pv10: KY195060 - KY195084
AN-Pv16: KY195085 - KY195108
AN-Pv17: KY195109 - KY195133
AN-Pv18: KY195134 - KY195158
AN-Pv22: KY195159 - KY195182
AN-Pv26.1: KY195183 - KY195228
AN-Pv28: KY195229 - KY195253
AN-Pv29: KY195254 - KY195278
AN-Pv30: KY195279 - KY195303
AN-Pv32: KY195304 - KY195328
AN-Pv33: KY195329 - KY195353
AN-Pv35: KY195354 - KY195378
AN-Pv44: KY195379 - KY195403
AN-Pv46: KY195404 - KY195427
AN-Pv47: KY195428-KY195452
AN-Pv48: KY195453-KY195474
AN-Pv51: KY195475-KY195497
AN-Pv54: KY195498-KY195520

AN-Pv55: KY195521-KY195545
AN-Pv57: KY195546 - KY195569
AN-Pv63: KY195570 - KY195594
AN-Pv64: KY195595 - KY195617
AN-Pv66: KY195618 - KY195642
AN-Pv68: KY195643 - KY195666
AN-Pv69: KY195667 - KY195690
AN-PvCO: KY195691 - KY195713
AN-TGA: KY195714 - KY195738
g510: KY195739 - KY195763
g523: KY195764 - KY195788
gssE18: KY195789 - KY195811
gssE19: KY195812 - KY195836
gssE20: KY195837 - KY195861
gssE28: KY195862 - KY195885
Leg443: KY195886 - KY195906
Leg044: KY195907; KY195911
Leg100: KY195908; KY195912
Leg133: KY195909; KY195913
Leg223: KY195910; KY195914
